# Efficacy and safety of 24 antibiotics for group A streptococcal pharyngitis: a network meta-analysis of 64 randomized controlled trials

**DOI:** 10.3389/fpubh.2026.1848949

**Published:** 2026-06-22

**Authors:** Xuemei Sun, Hongya Du, Yupeng Lei, Tiechao Ruan, Bixin Deng, Wenting Lu, Junjie Ying, Shiping Li, Ruixi Zhou, Dezhi Mu

**Affiliations:** 1 Department of Pediatrics, West China Second University Hospital, Sichuan University, Chengdu, China; 2 Key Laboratory of Birth Defects and Related Diseases of Women and Children (Sichuan University), Ministry of Education, Chengdu, China; 3 Department of Respiratory and Critical Care Medicine, Institute of Respiratory Health and Multimorbidity, Institute of Respiratory Health, Integrated Care Management Center, West China Hospital, Sichuan University, Chengdu, China

**Keywords:** adverse events, antibiotics, bacteriological eradication, clinical response, group A streptococcal pharyngitis, network meta-analysis

## Abstract

**Background and aims:**

Penicillin remains the first-line therapy for group A streptococcal (GAS) pharyngitis. However, broad-spectrum antibiotics continue to be widely prescribed in clinical practice. This study aimed to evaluate the relative efficacy and safety profiles of antibiotics for GAS pharyngitis.

**Methods:**

Literature published through March 2026 was searched. Efficacy outcomes included early and late bacterial eradication, clinical response, and bacteriological recurrence, whereas safety was assessed according to adverse events. Per-protocol data were extracted, and a Bayesian network meta-analysis was performed to estimate pooled odds ratios (ORs) with 95% confidence intervals (CIs).

**Results:**

We identified 64 RCTs (23,287 participants). For early bacterial eradication, cefdinir (OR: 3.09; 95% CI: 1.60–6.01) and cefpodoxime proxetil (OR: 2.58; 95% CI: 1.07–6.17) demonstrated greater efficacy compared with penicillin V, whereas azithromycin showed inferior eradication rates than penicillin V (OR: 0.53; 95% CI: 0.32–0.90). For late bacterial eradication, cefprozil demonstrated greater efficacy compared with penicillin V (OR: 3.12; 95% CI: 1.03–11.25), whereas azithromycin exhibited suboptimal performance (OR: 0.38; 95% CI: 0.25–0.62). For early clinical response, cefdinir (OR: 2.01; 95% CI: 1.28–3.25) and cefuroxime axetil (OR: 2.14; 95% CI: 1.19–3.60) demonstrated significantly greater efficacy compared with penicillin V. For late clinical response, spiramycin showed superior efficacy to penicillin V (OR: 0.17; 95% CI: 0.02–0.94), although evidence was limited to a single small trial. Azithromycin was associated with reduced efficacy, higher late recurrence rates, and increased adverse events.

**Conclusion:**

Standard penicillins should remain the preferred first-line therapy for GAS pharyngitis to support antimicrobial stewardship. Cefdinir represents an effective alternative. Conversely, azithromycin should be avoided due to inferior efficacy and increased adverse risks.

**Systematic review registration:**

http://www.crd.york.ac.uk/prospero, identifier CRD420251104703.

## 1 Introduction

Group A streptococcal (GAS) pharyngitis is an acute bacterial infection of the pharynx that commonly presents with a sore throat and fever. It affects approximately 5–15% of adult patients and 20–30% of pediatric patients presenting with pharyngitis (1, 2). The primary goals of treatment are symptom relief, prevention of rare but serious complications, and reduction of transmission to close contacts (3–5). Clinical guidelines recommend penicillin as first-line treatment for GAS pharyngitis, with cephalexin, cefadroxil, clindamycin, azithromycin, and clarithromycin reserved for patients with penicillin allergy (6, 7). Nonetheless, a broad range of antibiotics, including cephalosporins and macrolides, remain extensively prescribed in routine clinical practice. This discrepancy between guideline recommendations and real-world prescribing patterns may reflect the overestimation of penicillin allergy, time pressures in clinical decision-making, and concerns regarding adherence to longer treatment courses (8–11). Growing concerns about antibiotic resistance underscore the urgent need for rational antibiotic use and optimized treatment strategies (7, 12, 13).

Although numerous randomized controlled trials (RCTs) and conventional meta-analyses have evaluated antibiotic efficacy for GAS pharyngitis, most have focused on direct comparisons between two agents. Evidence comparing multiple antibiotics simultaneously remains limited (11, 14, 15). Conventional meta-analyses are unable to integrate direct and indirect evidence, limiting their clinical applicability. By contrast, network meta-analysis (NMA) combines both sources of evidence, providing more comprehensive and robust support for clinical decision-making (16, 17).

This study aimed to systematically review available RCTs and conduct an NMA to evaluate the comparative efficacy and safety of antibiotics for GAS pharyngitis. The findings provide evidence to support rational antibiotic selection and the optimization of treatment regimens.

## 2 Method

### 2.1 Search strategy, inclusion criteria, and study selection

The study protocol was registered in the PROSPERO database (CRD420251104703). The final report was prepared following PRISMA 2020 guidelines (Supplementary Table 1). Two reviewers independently conducted a comprehensive search of PubMed, Embase, Cochrane, and Web of Science databases for English-language RCTs published until March 30, 2026. The search strategy is shown in Supplementary Table 2. Eligible studies included patients with GAS pharyngitis receiving antibiotic monotherapy (e.g., penicillins, cephalosporins, macrolides) compared to placebo or active controls. Outcomes of interest were clinical resolution (early/late), bacterial eradication (early/late), recurrence, and adverse events. Exclusion criteria included non-randomized designs, combination therapies, and studies with non-extractable data. Disagreements were resolved through discussion with a third reviewer.

### 2.2 Outcome assessment

The primary outcomes included early and late clinical resolution, as well as early and late bacterial eradication, as defined in each original study. Secondary outcomes included bacteriological recurrence and adverse events. The definitions followed the original trials or standardized criteria, where available. For the clinical outcomes, we extracted data based on the definitions provided in the original trials. To ensure consistency, clinical effectiveness was uniformly defined as the composite of “cure” and “improved,” as reported by study authors. When the original study did not specify timing, early was defined as assessment within 21 days after treatment initiation, consistent with the most common follow-up period reported across the included trials (typically 10–21 days).

### 2.3 Data extraction

Two investigators independently extracted the data using Microsoft Excel. The extracted information included the study ID, year, country, diagnostic criteria, number of participants, sex, age, intervention and comparator antibiotics, dosage, and duration. For dichotomous variables (e.g., eradication), the number of events and the total number of participants were recorded. For continuous variables, the mean and standard deviation (SD) were extracted. All outcomes were extracted from per-protocol (PP) analyses, which were consistently available across all included trials. In studies reporting both intention-to-treat (ITT) and PP results, we preferentially used PP data to maintain consistency across comparisons.

### 2.4 Quality assessment and risk of bias

The risk of bias was assessed independently by two reviewers using the Risk of Bias (RoB) 2.0 (Cochrane Collaboration, London, UK). Discrepancies were resolved through discussion with a third reviewer. The risk of bias was categorized as low, concerning, or high.

### 2.5 Data synthesis and statistical analysis

A Bayesian NMA was conducted using R (version 4.5.1) and STATA (Stata Corp, College Station, TX, USA, version 17.0). Binary outcomes were analyzed using a binomial likelihood with a logit link function and non-informative priors. Random-effects models were applied to account for between-study heterogeneity. Results were reported as odds ratios (ORs) with 95% confidence intervals (CIs). Absolute event rates were estimated by applying NMA-derived ORs to the pooled event rate of the penicillin V group. Consistency was assessed using a design-by-treatment interaction model, with a deviance information criterion (DIC) difference of <5 considered acceptable. Local inconsistency was evaluated using node-splitting analysis. Subgroup analyses were conducted according to patient age and treatment duration. Small-study effects were assessed using comparison-adjusted funnel plots. Treatment rankings were summarized using the area under the cumulative ranking curve values. Statistical heterogeneity was quantified using the *I*^2^ statistic.

## 3 Results

### 3.1 Study selection

**Figure 1:**
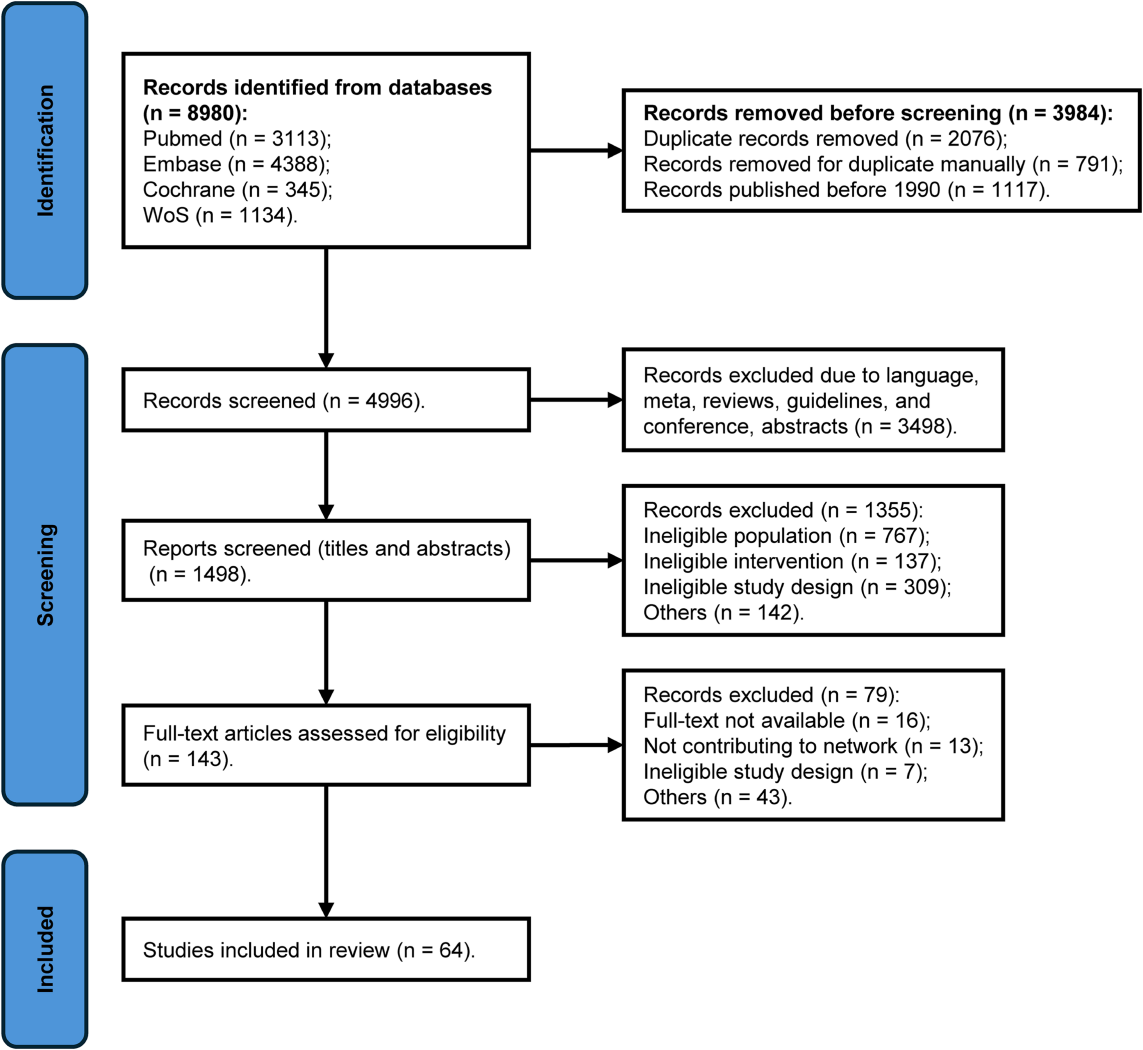
Study flow diagram. PRISMA flow diagram showing the identification, screening, eligibility assessment, and inclusion of studies.

The database search identified 8,980 articles. After removing 3,984 duplicates and studies published before 1990, 4,996 articles remained for screening. Following title and abstract screening, 1,498 articles were retained. Of these, 1,355 articles that did not meet the predefined population, intervention, or study design criteria were excluded. Finally, 143 full-text articles were assessed for eligibility, of which 79 were excluded, resulting in 64 RCTs included in the final analysis. The study selection process is presented in the Preferred Reporting Items for Systematic reviews and Meta-Analyses flow diagram (Figure 1). A full list of included studies is provided in Supplementary Appendix 1.

### 3.2 Characteristics of included studies

The included studies were published between 1990 and 2026 and conducted across multiple regions. Most studies originated in Europe, followed by North America, Asia, and Oceania. The sample size ranged from 60 to 1975 participants, with a total of 23,287 participants. The interventions included penicillin, amoxicillin, cephalosporins, and macrolides. Table 1 summarizes the study characteristics, including publication year, country, diagnostic criteria, intervention details (drug, dose, frequency, and duration), the number of participants, sex distribution, and age.

**Table 1:** Study characteristics of included trials.

Study	Year	Country	Diagnostic criteria	Intervention	Patients, *n* (%male)	Age, y	Dose	Frequency	Duration (days)
Li et al.	2019	China	Symptoms and signs + rapid antigen test + throat culture	Azithromycin	85 (47.0)	5.6 ± 2.3	10 mg/kg/day	QD	3
				Cefaclor	88 (56.0)	5.8 ± 2.3	20 mg/kg/day	TID	5
				Amoxicillin	83 (54.0)	5.9 ± 2.4	30 mg/kg/day	TID	10
Kuroki et al.	2012	Japan	Symptoms and signs + rapid antigen test	Amoxicillin-Clavulanate	54 (46.3)	5.6 (2–13)	Clavulanate 6.4 mg/kg/day, amoxicillin 90 mg/kg/day	BID	3
				Amoxicillin	43 (51.2)	5.3 (1–9)	30 mg/kg/day	TID	10
Koga et al.	2011	Japan	Symptoms and signs + rapid antigen test	Azithromycin	88 (44.3)	26.1 ± 16.5	500 mg/day (>16 y); 10 mg/kg/day (<16 y)	QD	3
				Cefcapene Pivoxil	71 (49.3)	24.6 ± 16.8	100 mg/day (>16 y), 3 mg/kg/day (<16 y)	TID	5
Rimoin et al.	2011	United States	Symptoms and signs + rapid antigen test + throat culture*	Benzathine Penicillin G	C: 84 (65.5) E: 191 (60.2)	C: 5.6 ± 2.6 E: 5.3 ± 2.6	600,000 U (<27 kg); 1.2 million U (> = 27 kg)	Once (IM)	1
				Amoxicillin	C: 82 (56.1) E: 201 (55.7)	C: 5.7 ± 2.4 E: 5.4 ± 2.5	750 mg/day	QD	10
Lennon et al.	2008	New Zealand	Symptoms and signs + throat culture	Amoxicillin	177 (52.0)	8.7 (7.1–10.2)	1,500 mg (> = 30 kg); 750 mg (<30 kg)	QD	10
				Penicillin V	176 (49.0)	8.5 (6.7–9.9)	500 mg (> = 20 kg); 250 mg (<20 kg)	BID	10
Pichichero et al.	2008	United States	Symptoms and signs + rapid antigen test + throat culture*	Amoxicillin	284 (45.1)	6.7 (2.78)	475 mg (6mo–4y); 775 mg (5–12y)	QD	7
				Penicillin V	282 (53.5)	7.0 (2.80)	10 mg/kg, max 250 mg	QID	10
Sakata et al.	2008	Japan	Symptoms and signs + rapid antigen test + throat culture	Cefcapene Pivoxil	82 (54.8)	5.5 ± 2.3	9–10 mg/kg/day	TID	5
				Amoxicillin	80 (57.1)	5.7 ± 2.4	30–40 mg/kg/day	TID	10
Mahakit et al.	2006	The Netherlands	Symptoms and signs + rapid antigen test + throat culture*	Clindamycin	384 (43.2)	28.1 (10.2)	300 mg	BID	10
				Amoxicillin-Clavulanate	390 (50.0)	27.9 (10.4)	1 g	BID	10
Kafetzis et al.	2004	Greece	Symptoms and signs + rapid antigen test + throat culture*	Penicillin V	91 (64.0)	6 (3–12)	50 mg/kg/day to 1.2 million IU	TID	10
				Clarithromycin	86 (48.0)	6 (3.5–13)	15 mg/kg/day to 500 mg/day	BID	10
				Cefprozil	88 (56.0)	6 (3–13)	30 mg/kg/day to 500 mg/day	BID	5
Scholz et al.	2004	Germany	Symptoms and signs + rapid antigen test + throat culture	Cefuroxime Axetil	496 (NA)	(1–17)	20 mg/kg/day (max 500 mg)	BID	5
				Penicillin V	1,456 (NA)		30 mg/kg/day	TID	10
Syrogiannopoulos et al.	2004	United States	Symptoms and signs + rapid antigen test + throat culture*	Clarithromycin	132 (51.5)	8 (2–16)	15 mg/kg/day (max 250 mg)	BID	5
				Amoxicillin-Clavulanate	135 (51.9)	7 (2–15)	43.8/6.2 mg/kg/day (max 1 g)	BID	5
				Penicillin V	135 (51.1)	7 (2–16)	30 mg/kg/day (max 450 mg/750,000 IU)	TID	10
Haczyński et al.	2003	Poland	Symptoms and signs + rapid antigen test	Cefaclor	49 (39.8)	6.7 ± 2.3	20 mg/kg/day	BID	10
				Amoxicillin-Clavulanate	51 (40.2)	6.1 ± 2.0	25 mg/kg/day	TID	10
Quinn et al.	2003	Canada	Symptoms and signs + rapid antigen test + throat culture*	Telithromycin	232 (36.2)	29 (13–72)	800 mg	QD	5
				Clarithromycin	231 (39.4)	29 (13–81)	250 mg	BID	10
Takker et al.	2003	United States	Symptoms and signs + rapid antigen test + throat culture*	Clarithromycin	270 (43.0)	28.9 (12.0)	500 mg/day	QD	5
				Penicillin V	267 (41.0)	29.9 (12.6)	500 mg	TID	10
Cohen et al.	2002	France	Symptoms and signs + rapid antigen test + throat culture*	Azithromycin	169 (55.6)	5.8 ± 2.3	10 mg/kg/day	QD	3
				Penicillin V	167 (52.1)	6.0 ± 2.2	45 mg/kg/day	TID	10
Esposito et al.	2002	Italy	Symptoms and signs + rapid antigen test + throat culture*	Cefaclor	175 (53.1)	5.3 (2–14)	40 mg/kg/day	BID	5
				Amoxicillin	173 (49.7)	4.9 (2–14)	40 mg/kg/day	TID	10
Portier et al.	2002	France	Symptoms and signs + rapid antigen test + throat culture*	Clarithromycin	177 (38.4)	27.8 ± 8.5	500 mg	QD	5
				Penicillin V	172 (41.2)	28.5 ± 9.3	590 mg (1 million units)	TID	10
Schaad et al.	2002	Switzerland	Symptoms and signs + rapid antigen test + throat culture*	Azithromycin	146 (NA)	NA	10 mg/kg/day (max 500 mg)	QD	3
				Penicillin V	146 (NA)		100,000 IU/kg/day (max 6,000,000 IU)	TID	10
Brook et al.	2001	United States	Symptoms and signs + rapid antigen test + throat culture*	Cefprozil	87 (49.0)	7.5 (2.5)	15 mg/kg/day	BID	10
				Erythromycin	85 (49.0)	7.3 (2.4)	30 mg/kg/day	TID	10
Haczyński et al.	2001	Poland	Symptoms and signs + rapid antigen test + throat culture*	Cefaclor	100 (39.0)	28.92 ± 11.26	375 mg	BID	10
				Amoxicillin-Clavulanate	100 (45.0)	28.65 ± 10.55	625 mg	BID	10
Kaplan et al.	2001	United States	Symptoms and signs + rapid antigen test + throat culture*	Clarithromycin	194 (37.0)	26.8 ± 11.5	250 mg	BID	10
				Azithromycin	198 (41.0)	26.1 ± 10.9	500 mg (day 1), 250 mg (day 2–5)	QD	5
Norrby et al.	2001	Sweden	Symptoms and signs + rapid antigen test + throat culture*	Telithromycin	198 (43.9)	32 (15–62)	800 mg	QD	5
				Penicillin V	197 (42.6)	33 (15–74)	500 mg	TID	10
Adam et al.	2000	Germany	Symptoms and signs + rapid antigen test + throat culture*	Cefuroxime Axetil	501 (49.8)	6.2 (1–16)	250 mg or 20 mg/kg/day (max 500 mg)	BID	5
				Penicillin V	1,474 (49.3)	6.0 (1–17)	50,000 IU/kg/day	TID	10
McCarty et al.	2000	United States	Symptoms and signs + rapid antigen test + throat culture*	Clarithromycin	268 (53.0)	7.4 ± 2.9	7.5 mg/kg	BID	5
				Penicillin V	260 (57.0)	7.7 ± 2.5	13.3 mg/kg (max 1,500 mg/day)	TID	10
Uysal et al.	2000	Turkey	Symptoms and signs + throat culture	Cefuroxime Axetil	42 (42.9)	8.7 ± 2.0	20 mg/kg/day	BID	10
				Benzathine Penicillin G	44 (52.3)	9.6 ± 2.1	1.2 million U	Once (IM)	1
Nemeth et al.	1999a	United States	Symptoms and signs + rapid antigen test + throat culture*	Cefdinir	264 (50.0)	7.0 (1–13)	7 mg/kg	BID	10
				Penicillin V	264 (50.4)	7.2 (2–12)	10 mg/kg	QID	10
Nemeth et al.	1999b	United States	Symptoms and signs + rapid antigen test + throat culture*	Cefdinir	304 (32.9)	26.5 (12–72)	300 mg	BID	10
				Penicillin V	310 (40.6)	25.0 (12–72)	250 mg	QID	10
Cremer et al.	1998	Germany	Symptoms and signs + throat culture	Azithromycin	62 (53.2)	6.0 (0.1–12.2)	10 mg/kg	QD	3
				Cefaclor	60 (52.0)	6.1 (0.9–11.7)	30 mg/kg/day	TID	10
Esposito et al.	1998	Italy	Symptoms and signs + rapid antigen test + throat culture	Cefaclor	85 (47.1)	6.4 (2–12)	25 mg/kg	BID	10
				Amoxicillin-Clavulanate	78 (46.2)	5.9 (3–10.5)	15 mg/kg	TID	10
				Erythromycin	82 (53.7)	6.8 (2.5–11)	15 mg/kg	TID	10
Gopichand et al.	1998	United States	Symptoms and signs + throat culture*	Penicillin V	79 (47.3)	8.2 ± 3.0	125 mg (<60 lbs) or 250 mg (> = 60 lbs)	TID	10
				Amoxicillin	82 (63.8)	8.5 ± 3.4	40 mg/kg/day	TID	10
Tack et al.	1998	United States	Symptoms and signs + rapid antigen test + throat culture*	Cefdinir	278 (NA)	26 (13–76)	300 mg	BID	5
				Penicillin V	280 (NA)		250 mg	QID	10
Venuta et al.	1998	Italy	Symptoms and signs + rapid antigen test + throat culture	Clarithromycin	63 (46.0)	8.1 (4–12.3)	7.5 mg/kg(maximum 500 mg/day)	BID	10
				Azithromycin	74 (49.0)	7.9 (4.1–11.9)	10 mg/kg (maximum 500 mg/day)	QD	3
Gendrel et al.	1997	France	Symptoms and signs + rapid antigen test + throat culture*	Spiramycin	149 (53.0)	6.7 ± 2.8	100,000 IU/kg	BID	5
				Penicillin V	149 (53.0)	6.7 ± 2.6	25,000 IU/kg	TID	7
Tack et al.	1997	United States	Symptoms and signs + rapid antigen test + throat culture*	Cefdinir	240 (53.3)	7.4 (1–13)	7 mg/kg (max 300 mg)	BID	5
				Penicillin V	242 (50.4)	7.7 (2–18)	10 mg/kg (max 250 mg)	QID	10
Watkins et al.	1997	United States	Symptoms and signs + rapid antigen test + throat culture*	Dirithromycin	170 (36.5)	28.3 ± 9.7	500 mg	QD	10
				Penicillin V	175 (36.0)	29.0 ± 9.4	250 mg	QID	10
Adam et al.	1996	Gamany	Symptoms and signs + rapid antigen test + throat culture*	Erythromycin	102 (52.9)	7.1 (3–17)	40 mg/kg/day (max 1800 mg)	BID	5
				Penicillin V	99 (49.5)	7.1 (3–13)	50,000 U/kg (max 2,225,000 U)	TID	10
Cohen et al.	1996	France	Symptoms and signs + rapid antigen test + throat culture*	Amoxicillin	160 (51.9)	5.9 ± 2.1	50 mg/kg/day	BID	6
				Penicillin V	158 (44.3)	5.9 ± 2.3	45 mg/kg/day	TID	10
O’Doherty et al.	1996	Ireland	Symptoms and signs + rapid antigen test + throat culture*	Azithromycin	166 (42.8)	7.7 ± 2.8	10 mg/kg	QD	3
				Penicillin V	163 (50.3)	7.7 ± 2.7	125 mg (<20 kg); 250 mg (> = 20 kg)	QID	10
Pacifico et al.	1996	Italy	Symptoms and signs + rapid antigen test + throat culture*	Azithromycin	76 (47.3)	6.7 (3–12)	10 mg/kg	QD	3
				Penicillin V	78 (50.0)	6.9 (3–12)	50,000 U/kg/day	BID	10
Peyramond et al.	1996	France	Symptoms and signs + rapid antigen test + throat culture*	Amoxicillin	165 (38.2)	32.2 ± 11.6	1 g	BID	6
				Penicillin V	173 (35.8)	34.0 ± 12.8	1 million U	TID	10
Schaad et al.	1996	Switzerland	Symptoms and signs + rapid antigen test + throat culture*	Azithromycin	170 (48.2)	7.1 (1.5–12.4)	10 mg/kg	QD	3
				Penicillin V	173 (51.4)	6.9 (1.9–13.9)	100,000 IU/kg/day	TID	10
Adam et al.	1995	Germany	Symptoms and signs + rapid antigen test + throat culture*	Cefixime	75 (62.7)	5.5 (2–11)	8 mg/kg	QD	5
				Penicillin V	76 (51.3)	5.4 (1–12)	20,000 IU/kg	TID	10
Aujard et al.	1995	France	Symptoms and signs + rapid antigen test + throat culture*	Cefuroxime Axetil	97 (47.4)	6.8 ± 3.5	20 mg/kg/day (max 500 mg)	BID	4
				Penicillin V	103 (44.7)	7.1 ± 3.0	45 mg/kg/day	TID	10
Carbon et al.	1995	France	Symptoms and signs + rapid antigen test + throat culture*	Cefotiam	119 (31.1)	32.2 ± 11.5	200 mg	BID	5
				Penicillin V	121 (42.2)	31.5 ± 10.3	600 mg	TID	10
Kaufhold et al.	1995	Germany	Symptoms and signs + throat culture*	Penicillin V	70 (40.0)	6.3 (3–11)	50,000 U/kg/day (max 1.5 million U)	TID	10
				Benzathine Penicillin V	131 (49.6)	6.4 (2–14)	50,000 U/kg/day (max 1.5 million U)	BID	10
Pichichero et al.	1994	United States	Symptoms and signs + rapid antigen test + throat culture*	Cefpodoxime Proxetil	126 (NA)	8.1 ± 3.4	10 mg/kg/day (maximum 200 mg)	BID	5
				Penicillin V	130 (NA)	8.0 ± 2.9	40 mg/kg/day (maximum 1,000 mg)	TID	10
Portier et al.	1994	France	Symptoms and signs + rapid antigen test + throat culture*	Cefpodoxime Proxetil	113 (54.0)	30.4 ± 12.1	100 mg	BID	5
				Penicillin V	107 (49.5)	28.6 ± 11.2	600 mg	TID	10
Dajani et al.	1993	United States	Symptoms and signs + rapid antigen test + throat culture*	Cefpodoxime Proxetil	275 (54.5)	8.4 ± 3.6	10 mg/kg/day (maximum 200 mg)	BID	10
				Penicillin V	138 (51.4)	8.3 ± 3.3	40 mg/kg/day (max 1,000 mg)	TID	10
Gooch et al.	1993	United States	Symptoms and signs + throat culture*	Cefuroxime Axetil	259 (47.1)	6.7 (2–12)	20 mg/kg/day (max 500 mg)	BID	10
				Penicillin V	126 (42.9)	6.9 (2–13)	50 mg/kg/day (max, 750 mg)	TID	10
Hamill et al.	1993	United Kingdom	Symptoms and signs + rapid antigen test + throat culture*	Azithromycin	49 (53.1)	7.4 (2–12)	10 mg/kg	QD	3
				Penicillin V	47 (53.2)	7.5 (3–12)	125 mg (<20 kg); 250 mg (> = 20 kg)	QID	10
Müller et al.	1993	Germany	Symptoms and signs + rapid antigen test + throat culture*	Dirithromycin	193 (39.4)	30.4 ± 11.4	500 mg	QD	10
				Erythromycin	196 (42.9)	31.3 ± 11.5	250 mg/day	QID	10
Ruggiero et al.	1993	Italy	Symptoms and signs + rapid antigen test + throat culture*	Dirithromycin	30 (40.0)	27 (18–49)	500 mg	QD	10
				Miocamycin	30 (46.7)	30 (18–52)	600 mg	BID	10
Weippl et al.	1993	Austria	Symptoms and signs + rapid antigen test + throat culture*	Azithromycin	46 (56.5)	5.4 (1–12)	10 mg/kg/day (max, 500 mg)	QD	3
				Erythromycin	47 (48.9)	5.0 (2–12)	30–50 mg/kg/day	TID	10
Block et al.	1992	United States	Symptoms and signs + rapid antigen test + throat culture*	Cefixime	55 (52.7)	9.3 ± 3.2	8 mg/kg (<=50 kg); 400 mg (> = 12 years or >50 kg)	QD	10
				Penicillin V	55 (38.2)	8.9 ± 3.0	250 mg	TID	10
Disney et al.	1992a	United States	Symptoms and signs + throat culture	Cephalexin	263 (55.1)	8.6	27 mg/kg/day	QID	10
				Penicillin V	262 (48.1)	8.1	27 mg/kg/day	QID	10
Disney et al.	1992b	United States	Symptoms and signs + rapid antigen test + throat culture*	Loracarbef	120 (54.2)	7.3 ± 2.6	200 mg or 15 mg/kg/day (max, 375 mg)	BID	10
				Penicillin V	113 (46.9)	7.2 ± 2.6	250 mg or 20 mg/kg/day (max, 500 mg)	QID	10
McCarty et al.	1992	United States	Symptoms and signs + rapid antigen test + throat culture*	Loracarbef	107 (36.7)	30.3 (12–65)	200 mg or 15 mg/kg/day (max, 375 mg)	BID	10
				Penicillin V	111 (0.0)		250 mg or 20 mg/kg/day (max, 500 mg)	QID	10
Müller et al.	1992	Germany	Symptoms and signs + rapid antigen test + throat culture*	Loracarbef	169 (45.6)	28.2 ± 13.8	200 mg or 15 mg/kg/day (max, 375 mg)	BID	10
				Penicillin V	175 (40.6)	28.2 ± 14.7	250 mg or 20 mg/kg/day (max, 500 mg)	QID	10
Ramet et al.	1992	Belgium	Symptoms and signs + rapid antigen test + throat culture*	Cefetamet Pivoxil	37 (NA)	6.3	20 mg/kg	BID	10
				Penicillin V	40 (NA)	5.1	20,000 IU/kg (max 750,000 IU/dose)	TID	10
Bachand et al.	1991	United States	Symptoms and signs + rapid antigen test + throat culture*	Clarithromycin	43 (30.2)	26.0 ± 8.6	250 mg	BID	NA
				Penicillin V	47 (25.5)	27.7 ± 10.8	250 mg	Q6h	NA
Christenson et al.	1991	United States	Symptoms and signs + throat culture*	Cefprozil	50 (NA)	33.6 (14–66)	500 mg	QD	10
				Cefaclor	28 (NA)	31.2 (13–50)	250 mg	TID	10
Hooton et al.	1991	United States	Symptoms and signs + rapid antigen test + throat culture*	Azithromycin	152 (44.7)	NA	500 mg (day 1), 250 mg (day 2–5)	QD	5
				Penicillin V	90 (36.7)		250 mg	Q6h	10
Stein et al.	1991	United States	Symptoms and signs + rapid antigen test + throat culture*	Clarithromycin	65 (36.9)	28 (12–54)	250 mg	BID	10
				Penicillin V	63 (23.8)	29 (12–58)	250 mg	Q6h	10
Scaglione et al.	1990	Italy	Symptoms and signs + throat culture	Clarithromycin	120 (NA)	43.97 ± 16.65	250 mg	BID	10
				Erythromycin	120 (NA)		500 mg	BID	10

*Indicates that participants were enrolled regardless of culture result, but only culture-positive cases were included in the outcome analysis. QD, once daily; BID, twice daily; TID, three times daily; QID, four times daily; Q6h, every 6 h; IM, intramuscular; NA, not available.

### 3.3 Risk of bias assessment

The risk of bias assessment for all included studies is presented in Supplementary Figure 1. Overall, 47 of the 64 studies were judged to have a low risk of bias, 17 were rated as having some concerns, and none were classified as high risk. The most common methodological limitations were lack of blinding and incomplete outcome data.

### 3.4 Network geometry

**Figure 2:**
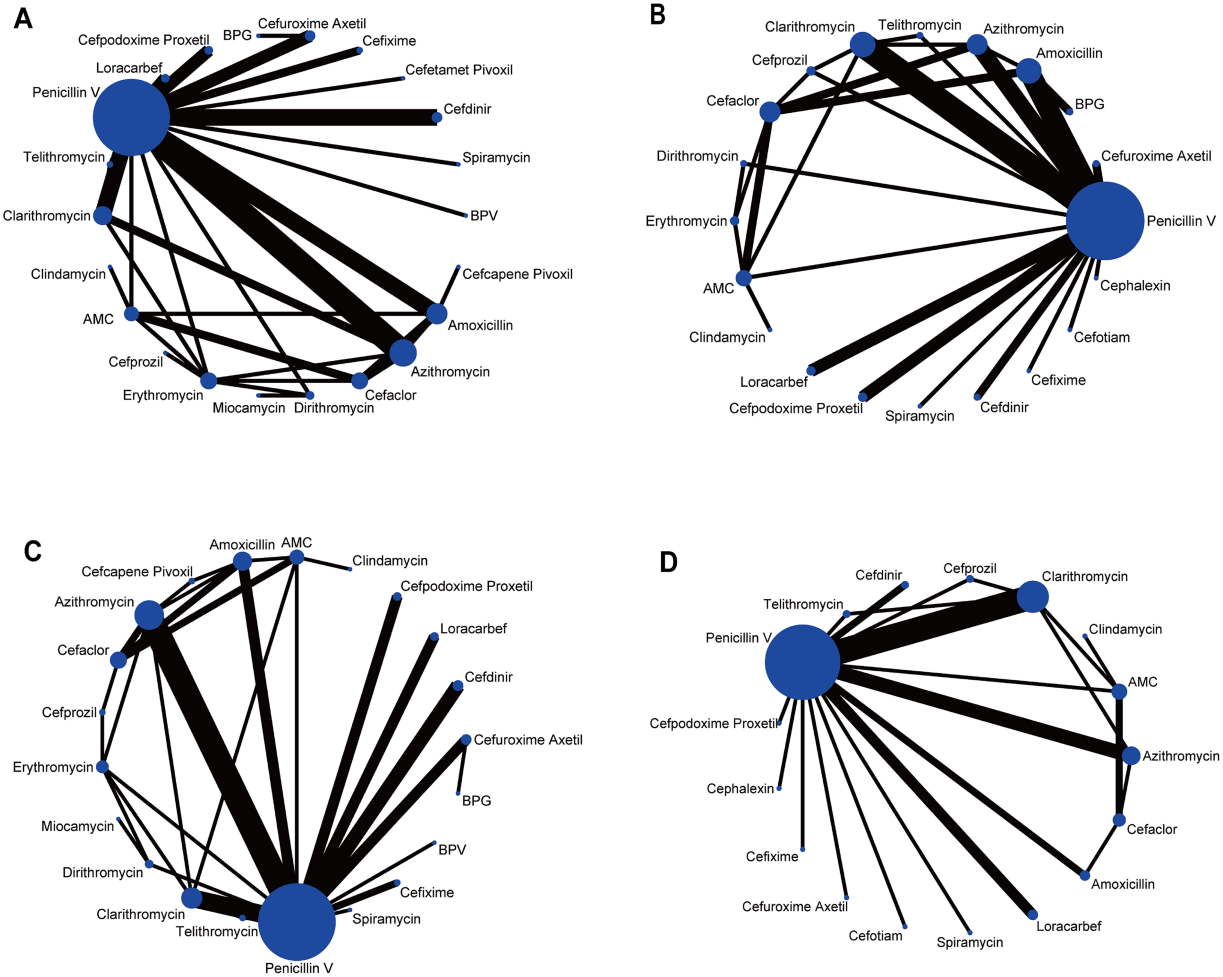
Network plots of primary outcomes. Network plots showing the geometry of evidence for **(A)** early bacterial eradication, **(B)** late bacterial eradication, **(C)** early clinical response, and **(D)** late clinical response. Node size is proportional to the number of participants receiving the treatment; line thickness represents the number of direct comparisons. AMC, amoxicillin-clavulanate; BPG, Benzathine Penicillin G; BPV, Benzathine Penicillin V.

The evidence network comprised 24 antibiotic regimens (Figure 2). Penicillin was the most frequently evaluated intervention (62 arms), followed by macrolides (35 arms) and cephalosporins (32 arms). Other agents, such as lincosamides (clindamycin, one arm) and ketolides (telithromycin, two arms), were rarely assessed. No study directly compared all interventions simultaneously. Consequently, the NMA incorporated both direct and indirect evidence. The network was well connected, enabling multiple comparisons across interventions.

### 3.5 Assessment of transitivity, consistency, and heterogeneity

Diagnostic criteria for GAS pharyngitis and treatment regimens were consistent across studies, and antibiotic doses were weight-adjusted where applicable. Definitions of clinical cure and bacterial eradication were also largely comparable. Subgroup analyses based on age and treatment duration were performed to further explore clinical heterogeneity, supporting the transitivity assumption. No significant global inconsistency was observed for the primary or secondary outcomes (all *p* > 0.05). Among the loops examined across the four primary outcomes, only one showed significant inconsistency (*p* = 0.028 for amoxicillin-clavulanate vs. azithromycin in late bacterial eradication), whereas all other loops demonstrated consistency. The remaining loops demonstrated good agreement between direct and indirect comparisons, supporting network consistency. Heterogeneity remained low, with *I*^2^ values ranging from 0 to 7% across the primary outcomes.

### 3.6 Primary outcomes

#### 3.6.1 Early bacteriological eradication

A total of 53 studies reported dichotomous outcomes for early bacteriological eradication (Figure 2A). The league table (Supplementary Table 27) indicated that cefdinir (OR: 3.09, 95% CI: 1.60–6.01) and cefpodoxime proxetil (OR: 2.58, 95% CI: 1.07–6.17) achieved higher eradication rates compared with penicillin V. Cefdinir also outperformed amoxicillin (OR: 0.31, 95% CI: 0.13–0.74), amoxicillin-clavulanate (OR: 0.31, 95% CI: 0.11–0.90), and cefaclor (OR: 0.32, 95% CI: 0.12–0.90). Conversely, azithromycin showed lower eradication rates compared with penicillin V (OR: 0.53, 95% CI: 0.32–0.90). It was less effective compared with several agents, including cefprozil (OR: 0.11, 95% CI: 0.02–0.68) and clarithromycin (OR: 0.40, 95% CI: 0.20–0.80). The observed absolute eradication rates were 83.9% for penicillin V, 94.2% (95% CI: 89.3–96.9%) for cefdinir, 93.1% (95% CI: 84.8–97.0%) for cefpodoxime proxetil, and 73.4% (95% CI: 62.5–82.4%) for azithromycin. No publication bias was observed (Supplementary Figure 2A).

In the pediatric group, cefdinir remained significantly superior to penicillin V (OR: 4.98, 95% CI: 1.61–15.54). Azithromycin remained inferior to cefdinir (OR: 0.12, 95% CI: 0.03–0.47) and cefpodoxime proxetil (OR: 0.21, 95% CI: 0.06–0.84) (Supplementary Table 3). In the adult subgroup, azithromycin was also inferior to loracarbef (OR: 0.15, 95% CI: 0.03–0.81, Supplementary Table 4). Subgroup analysis by treatment duration revealed that 10-day cefdinir remained significantly superior to 10-day penicillin V (OR: 3.99, 95% CI: 1.33–12.2) and five other regimens. However, no significant difference was observed between 5-day cefdinir and 10-day penicillin V (Supplementary Table 5).

#### 3.6.2 Late bacterial eradication

Forty-two studies reported late bacterial eradication (Figure 2B). League table (Supplementary Table 28) showed that cefprozil was the only antibiotic with significantly higher eradication rates compared with penicillin V (OR: 3.12, 95% CI: 1.03–11.25). Conversely, azithromycin exhibited significantly lower eradication rates compared with penicillin V (OR: 0.38, 95% CI: 0.25–0.62). Other regimens demonstrated comparable efficacy to penicillin V. Several *β*-lactam antibiotics demonstrated superiority over macrolides. Cefprozil outperformed azithromycin (OR: 0.12, 95% CI: 0.03–0.40), clarithromycin (OR: 4.54, 95% CI: 1.46–16.52), and telithromycin (OR: 3.84, 95% CI: 1.02–16.24). Cefuroxime axetil also showed higher efficacy compared with azithromycin (OR: 0.22, 95% CI: 0.10–0.48) and clarithromycin (OR: 2.48, 95% CI: 1.19–5.48). Conversely, amoxicillin-clavulanate was superior to azithromycin (OR: 4.13, 95% CI: 1.86–8.83) and clarithromycin (OR: 2.29, 95% CI: 1.11–4.68). Overall, azithromycin ranked the lowest, demonstrating significantly lower eradication rates compared with 12 other interventions across the network. The absolute eradication rate for the reference treatment (penicillin V) was 84.3%. Cefprozil achieved a higher absolute eradication rate of 94.4% (95% CI: 84.6–98.4%), whereas azithromycin showed a lower rate of 67.0% (95% CI: 57.2–76.8%). The funnel plot indicated no evidence of publication bias (Supplementary Figure 2B).

In the pediatric group, cefprozil remained superior to penicillin V (OR: 6.08, 95% CI: 1.08–57.27). Azithromycin was inferior to penicillin V (OR: 0.40, 95% CI: 0.22–0.79), cefpodoxime proxetil, cefprozil, and cefuroxime axetil (Supplementary Table 6). Conversely, no significant differences were observed among treatments in the adult subgroup (Supplementary Table 7). When stratified by treatment duration, 5-day cefprozil was superior to 17 regimens, including 10-day penicillin V (OR: 8.07, 95% CI: 1.78–64.89). In addition, 10-day cefuroxime axetil outperformed 10-day penicillin V (OR: 3.07, 95% CI: 1.22–7.98). By contrast, 3-day azithromycin was less effective compared with the other 14 regimens, including 10-day penicillin V (OR: 0.41, 95% CI: 0.26–0.68), and 5-day clarithromycin was inferior to 10-day penicillin V (OR: 0.51, 95% CI: 0.29–0.88) (Supplementary Table 8).

#### 3.6.3 Early clinical response

A total of 53 studies reported early clinical response outcomes (Figure 2C). The league table (Supplementary Table 29) showed that cefdinir (OR: 2.01, 95% CI: 1.28–3.25) and cefuroxime axetil (OR: 2.14, 95% CI: 1.19–3.60) achieved significantly higher early clinical response rates compared with penicillin V. Cefdinir also demonstrated advantages over commonly used antibiotics, such as amoxicillin (OR: 0.33, 95% CI: 0.16–0.65) and azithromycin (OR: 0.52, 95% CI: 0.26–0.99). Conversely, amoxicillin did not differ significantly from penicillin V but was inferior to several other treatments. These included cefixime (OR: 0.26, 95% CI: 0.07–0.84), cefpodoxime proxetil (OR: 0.42, 95% CI: 0.18–0.97), cefuroxime axetil (OR: 0.31, 95% CI: 0.15–0.68), and clindamycin (OR: 0.18, 95% CI: 0.05–0.70). The absolute cure rate for penicillin V was 92.9%, compared with 96.3% for cefdinir (95% CI: 94.3–97.7%) and 96.5% for cefuroxime axetil (95% CI: 93.9–97.9%). The funnel plot (Supplementary Figure 2C) showed no evidence of publication bias.

In the pediatric subgroup, no treatments showed significant differences compared with penicillin V (Supplementary Table 9). However, amoxicillin remained inferior to cefdinir (OR: 0.30, 95% CI: 0.09–0.86) and cefuroxime axetil (OR: 0.29, 95% CI: 0.11–0.81). No significant differences were observed in the adult subgroup (Supplementary Table 10). When stratified by treatment duration, 10-day cefdinir outperformed nine regimens, including 10-day penicillin V (OR: 3.63, 95% CI: 1.84–7.43) and 5-day cefdinir (OR: 2.81, 95% CI: 1.14–7.10). Additionally, 10-day cefixime (OR: 5.72, 95% CI: 1.45–32.04) and 5-day cefuroxime axetil (OR: 2.46, 95% CI: 1.40–4.40) were superior to 10-day penicillin V (Supplementary Table 11).

#### 3.6.4 Late clinical response

Thirty-two studies reported late clinical response outcomes (Figure 2D). The league table (Table 2) showed that spiramycin was the only antibiotic that significantly outperformed penicillin V (OR: 0.17, 95% CI: 0.02–0.94). Moreover, spiramycin achieved higher cure rates compared with azithromycin (OR: 0.12, 95% CI: 0.01–0.73), clarithromycin (OR: 0.13, 95% CI: 0.01–0.74), and loracarbef (OR: 0.13, 95% CI: 0.01–0.88). Similarly, cephalexin showed superior outcomes compared with clarithromycin (OR: 3.85, 95% CI: 1.07–15.48) and azithromycin (OR: 0.24, 95% CI: 0.06–0.93). Meanwhile, cefuroxime axetil achieved higher cure rates compared with clarithromycin (OR: 3.49, 95% CI: 1.10–12.61) and azithromycin (OR: 0.27, 95% CI: 0.07–0.91). The absolute cure rate for penicillin V was 91.2%, compared with 98.4% (95% CI: 91.7–99.8%) for spiramycin. Funnel plot assessment showed no evidence of publication bias (Supplementary Figure 2D).

**Table 2:** League table of pairwise comparisons for late clinical response.

**Amoxicillin**																
1.69 (0.57, 5.23)	**AMC**															
2.08 (0.78, 5.73)	1.23 (0.42, 3.53)	**Azithromycin**														
1.09 (0.42, 2.86)	0.65 (0.26, 1.55)	0.52 (0.19, 1.44)	**Cefaclor**													
0.82 (0.21, 2.79)	0.49 (0.12, 1.77)	0.4 (0.12, 1.14)	0.75 (0.18, 2.76)	**Cefdinir**												
1.44 (0.2, 12.06)	0.86 (0.11, 7.54)	0.7 (0.1, 5.36)	1.33 (0.17, 11.9)	1.77 (0.24, 16.06)	**Cefixime**											
1.41 (0.29, 6.61)	0.84 (0.15, 4.07)	0.68 (0.15, 2.82)	1.29 (0.24, 6.41)	1.71 (0.36, 9.08)	0.96 (0.09, 8.86)	**Cefotiam**										
4.91 (0.36, 194.19)	2.93 (0.2, 124.93)	2.37 (0.18, 99.3)	4.48 (0.32, 184.96)	6.11 (0.44, 251.62)	3.53 (0.14, 191.58)	3.54 (0.22, 169.2)	**Cefpodoxime Proxetil**									
0.93 (0.03, 12.43)	0.56 (0.02, 7.26)	0.46 (0.01, 5.29)	0.86 (0.03, 11.82)	1.15 (0.04, 16.18)	0.64 (0.01, 13.32)	0.66 (0.02, 10.6)	0.17 (0, 6.44)	**Cefprozil**								
0.56 (0.13, 2.19)	0.33 (0.07, 1.37)	**0.27 (0.07, 0.91)**	0.52 (0.11, 2.15)	0.68 (0.16, 3.06)	0.38 (0.04, 3.17)	0.4 (0.07, 2.21)	0.11 (0, 1.63)	0.6 (0.04, 20.28)	**Cefuroxime Axetil**							
0.51 (0.11, 2.21)	0.3 (0.06, 1.35)	**0.24 (0.06, 0.93)**	0.47 (0.09, 2.13)	0.61 (0.13, 3.1)	0.35 (0.03, 3.05)	0.36 (0.06, 2.2)	0.1 (0, 1.61)	0.54 (0.04, 18.45)	0.91 (0.17, 4.95)	**Cephalexin**						
1.96 (0.77, 5.1)	1.16 (0.43, 3.09)	0.94 (0.48, 1.87)	1.8 (0.66, 4.98)	2.37 (0.89, 7.53)	1.36 (0.18, 8.91)	1.39 (0.36, 5.83)	0.4 (0.01, 4.92)	2.03 (0.19, 62.29)	**3.49 (1.1, 12.61)**	**3.85 (1.07, 15.48)**	**Clarithromycin**					
1.82 (0.37, 9.77)	1.08 (0.32, 3.73)	0.88 (0.18, 4.53)	1.68 (0.38, 7.85)	2.22 (0.39, 15.25)	1.26 (0.11, 14.1)	1.3 (0.18, 10.48)	0.36 (0.01, 6.95)	1.94 (0.12, 81.64)	3.25 (0.52, 23.96)	3.63 (0.53, 28.01)	0.94 (0.2, 4.58)	**Clindamycin**				
1.89 (0.55, 6.3)	1.13 (0.29, 3.96)	0.91 (0.31, 2.55)	1.74 (0.47, 6.2)	2.3 (0.67, 8.73)	1.31 (0.15, 9.71)	1.35 (0.28, 6.56)	0.38 (0.01, 5.32)	2.01 (0.15, 62.8)	3.39 (0.84, 14.24)	3.74 (0.83, 17.14)	0.97 (0.35, 2.52)	1.03 (0.16, 5.82)	**Loracarbef**			
1.47 (0.62, 3.37)	0.87 (0.32, 2.2)	0.71 (0.37, 1.26)	1.35 (0.5, 3.45)	1.79 (0.73, 4.83)	1.02 (0.14, 6.11)	1.04 (0.28, 3.9)	0.3 (0.01, 3.47)	1.54 (0.14, 45.34)	2.64 (0.87, 8.2)	2.91 (0.85, 10.29)	0.76 (0.45, 1.17)	0.81 (0.16, 3.59)	0.78 (0.33, 1.83)	**PenicillinV**		
0.25 (0.02, 1.68)	0.15 (0.01, 1.01)	**0.12 (0.01, 0.73)**	0.23 (0.02, 1.59)	0.3 (0.03, 2.25)	0.16 (0.01, 2.11)	0.17 (0.01, 1.53)	0.05 (0, 1.04)	0.26 (0.01, 11.21)	0.44 (0.04, 3.5)	0.48 (0.04, 4.1)	**0.13 (0.01, 0.74)**	0.13 (0.01, 1.29)	**0.13 (0.01, 0.88)**	**0.17 (0.02, 0.94)**	**Spiramycin**	
1.33 (0.38, 4.55)	0.79 (0.21, 2.79)	0.64 (0.21, 1.82)	1.22 (0.32, 4.4)	1.62 (0.45, 6.45)	0.92 (0.11, 6.93)	0.94 (0.19, 4.75)	0.27 (0.01, 3.71)	1.4 (0.11, 44.84)	2.37 (0.57, 10.56)	2.61 (0.57, 12.59)	0.68 (0.26, 1.65)	0.73 (0.12, 4.09)	0.7 (0.2, 2.5)	0.9 (0.36, 2.31)	5.36 (0.77, 57.03)	**Telithromycin**

Values are presented as odds ratios (95% confidence intervals). Penicillin V is used as reference. Statistically significant results are highlighted in bold. AMC, amoxicillin/clavulanate.

In the pediatric subgroup (Supplementary Table 12), spiramycin remained superior to clarithromycin (OR: 0.11, 95% CI: 0.01–0.84) and azithromycin (OR: 0.11, 95% CI: 0.01–0.84). No significant differences were observed in the adult subgroup (Supplementary Table 13). When stratified by treatment duration, no antibiotics demonstrated significant differences in late clinical response (Supplementary Table 14). Notably, spiramycin was excluded from this subgroup analysis due to network disconnection.

### 3.7 Sensitivity analysis

To assess the impact of increasing macrolide resistance, a sensitivity analysis was conducted including only trials published after 2005. This subset included six trials for early bacterial eradication, five for late eradication, and six for early clinical response. Late clinical response was excluded due to insufficient data. No significant differences were observed between antibiotics across these outcomes (Supplementary Tables 15–17). Notably, only two recent trials evaluated azithromycin for early clinical response, and only one provided bacteriological outcome data.

### 3.8 Secondary outcomes

#### 3.8.1 Bacteriological recurrence

Early bacteriological recurrence did not differ significantly between antibiotics and penicillin V (Supplementary Table 18). For late recurrence (Supplementary Table 19), azithromycin was the only antibiotic associated with a significantly higher recurrence rate compared with penicillin V (OR: 2.08, 95% CI: 1.18–3.53). Notably, azithromycin was associated with higher late recurrence rates than several other treatments, including amoxicillin-clavulanate (OR: 0.17, 95% CI: 0.03–0.88), cefaclor (OR: 3.8, 95% CI: 1.39–10.96), cefotiam (OR: 11.11, 95% CI: 1.11–363.98), cefpodoxime proxetil (OR: 2.46, 95% CI: 1.03–5.57), cefprozil (OR: 4.25, 95% CI: 1.43–12.9), and erythromycin (OR: 2.68, 95% CI: 1.22–5.87). Funnel plots for early and late bacteriological recurrence (Supplementary Figures 2E,F) showed no evidence of small-study effects or publication bias.

#### 3.8.2 Safety

Adverse events were reported in 13 to 32 studies. Compared with penicillin V, azithromycin (OR: 3.69, 95% CI: 1.93–7.13), erythromycin (OR: 2.52, 95% CI: 1.04–6.26), and clarithromycin (OR: 1.53, 95% CI: 1.01–2.40) were associated with a significantly higher risk of overall adverse events (Supplementary Table 20). Azithromycin also showed higher risks compared with cefaclor (OR: 4, 95% CI: 1.16–13.70), cefdinir (OR: 2.63, 95% CI: 1.10–6.42), cefotiam (OR: 6.85, 95% CI: 1.81–25.88), clarithromycin (OR: 2.41, 95% CI: 1.12–5.13), loracarbef (OR: 3.44, 95% CI: 1.38–8.65), and spiramycin (OR: 4.51, 95% CI: 1.19–17.23). By contrast, amoxicillin was associated with significantly fewer overall adverse events than both azithromycin (OR: 0.2, 95% CI: 0.07–0.61) and erythromycin (OR: 0.3, 95% CI: 0.09–0.94).

Several antibiotics were associated with a higher risk of gastrointestinal adverse events compared with penicillin V. For diarrhea, significantly increased risks were observed for amoxicillin, amoxicillin-clavulanate, cefprozil, erythromycin, and telithromycin (Supplementary Table 21). For vomiting, cefixime was associated with a lower risk compared with nine other antibiotics, including penicillin V (Supplementary Table 22). No statistically significant differences were observed for nausea across treatments (Supplementary Table 23). For abdominal pain, cefixime was associated with a higher risk of abdominal pain compared with penicillin V, whereas cefprozil and cefuroxime axetil demonstrated a lower risk (Supplementary Table 24).

With regard dermatological events, cefprozil was associated with a higher risk of rash compared with penicillin V, whereas cefcapene pivoxil exhibited a lower risk (Supplementary Table 25). In terms of neurological adverse events, headache occurred more frequently with cefaclor and azithromycin, whereas miocamycin was associated with a lower rate compared with penicillin V (Supplementary Table 26).

Funnel plots for adverse events (Supplementary Figures 2G–M) showed no evidence of small-study effects or publication bias.

## 4 Discussion

This NMA synthesized evidence from 23,287 participants across 64 RCTs to evaluate the efficacy and safety of 24 antibiotic treatments for GAS pharyngitis. By systematically ranking treatments against penicillin V, this study aimed to identify alternative regimens with improved efficacy or safety profiles (11, 15, 18, 19). The main findings indicate cefdinir is a highly effective alternative to penicillin V, with significantly superior early bacteriological and clinical outcomes and a lower overall risk of adverse events. Cefprozil was the only regimen that significantly outperformed penicillin V in late bacteriological eradication and was also associated with reduced late recurrence. Conversely, azithromycin performed poorly across both clinical and bacteriological outcomes, with a significantly higher risk of late recurrence and adverse events.

Effective bacterial eradication is essential to reduce communicability within 24 h and to prevent complications, such as peritonsillar abscess and rheumatic fever (20, 21). Among the evaluated antibiotics, cefdinir and cefprozil demonstrated notable advantages in early and late bacterial clearance, respectively. This superiority may be attributed to their enhanced *β*-lactamase stability, which maintains effective drug concentrations at the infection site and supports oral microbiome homeostasis, thereby reducing the risk of GAS recolonization (22, 23). By contrast, azithromycin exhibited poorer bacteriological outcomes, primarily driven by increasing macrolide resistance associated with widespread use. In particular, ribosomal methylation in GAS reduces drug binding affinity at the target site, whereas mef-encoded efflux pumps decrease intracellular drug concentrations, collectively impairing sustained antibacterial activity (24, 25). Furthermore, pharmacokinetic and pharmacodynamic limitations, including suboptimal tissue penetration and prolonged sub-inhibitory exposure, may contribute to incomplete bacterial eradication and persistent carriage. These conditions promote resistant strain recolonization and transmission among close contacts, resulting in higher recurrence rates (26, 27). Overall, these factors reduce the clinical utility of azithromycin compared with *β*-lactam antibiotics.

Although bacterial eradication is essential to prevent long-term adverse outcomes, achieving rapid clinical resolution is an equally important treatment goal. Given that acute symptoms such as sore throat and dysphagia substantially impair daily functioning, prompt symptom relief is highly prioritized by patients, particularly in the context of reduced risk of severe complications with routine antibiotic therapy (20, 28). Amoxicillin is widely used in clinical practice (29). Although it did not differ significantly from penicillin V, its early clinical response was significantly inferior to that of clindamycin and several cephalosporins, including cefdinir, cefixime, cefpodoxime proxetil, and cefuroxime axetil. This finding is consistent with that reported by Gualtieri et al. (30). In this trial, amoxicillin showed no significant benefit over placebo in terms of fever duration, pain severity, or complication risk. The superior early symptom relief observed with these alternative antibiotics may be explained by pharmacokinetic advantages, including improved tissue penetration and prolonged half-lives. Additionally, their broader antimicrobial spectra may facilitate the eradication of concomitant pathogens, accelerating clinical recovery (31, 32). With regard to late clinical response, azithromycin performed poorly, consistent with the higher bacteriological recurrence rates described above. Although spiramycin showed superior late clinical outcomes, this finding should be interpreted with caution due to the limited sample size. Consequently, ranking probabilities should not be equated with definitive clinical superiority, and treatment decisions should consider both the quantity and quality of available evidence. Overall, these findings underscore the importance of balancing rapid symptom relief with sustained bacteriological eradication when selecting optimal therapies.

Subgroup analyses provided additional insights into the main findings. In the pediatric population, cefdinir and cefprozil maintained significant bacteriological advantages over penicillin V. However, these differences were not consistently observed for clinical outcomes. Given that children accounted for the majority of participants with GAS pharyngitis (42 of 64 trials), these bacteriological benefits remain clinically relevant for this population. In adults, no significant differences were observed, except for the inferiority of azithromycin compared with loracarbef. This lack of significance likely reflects limited data availability, as only 8–12 trials contributed to each outcome across multiple regimens, resulting in reduced statistical power. Furthermore, the self-limiting nature of adult infections may attenuate observed treatment differences (6, 7). When stratified by treatment duration, optimal treatment periods varied across antibiotic agents and endpoints. For cefdinir, the 10-day regimen maintained superiority over penicillin V across early endpoints and outperformed its 5-day counterpart in clinical response. Conversely, 5-day regimens of cefprozil and cefuroxime axetil showed superiority in late bacteriological and early clinical outcomes, respectively. Notably, 10-day cefuroxime axetil revealed late bacteriological benefits not observed in the primary analysis. Regimens such as cefpodoxime proxetil lost statistical significance due to reduced power, though consistent effect directions suggested no true inferiority. These findings provided a more granular perspective than previous meta-analyses that aggregated various cephalosporins into a single category (19). Sensitivity analysis restricted to post-2005 trials showed no significant differences among treatments. This likely reflects extreme data sparsity rather than true clinical equivalence. The limited number of contemporary trials substantially reduced the statistical power to detect differences for azithromycin. Consequently, these restricted findings do not alter the primary conclusions regarding azithromycin efficacy.

Safety profiles varied considerably across antibiotic classes in this NMA. Macrolides, particularly azithromycin, clarithromycin, and erythromycin, were associated with a higher risk of overall adverse events compared with penicillin V. Specifically, erythromycin was associated with an increased risk of diarrhea. This is likely driven by motilin receptor agonism, which stimulates gastrointestinal motility (33). Azithromycin was also associated with a higher incidence of neurological events, such as headache. By contrast, penicillin V demonstrated a favorable safety profile, consistent with longstanding clinical experience (34). Although amoxicillin was associated with fewer overall adverse events compared with macrolides, both amoxicillin and amoxicillin-clavulanate exhibited a significantly higher risk of diarrhea compared with penicillin V. This effect may be explained by disruption of the gut microbiota and an increased intestinal osmotic load related to clavulanate (35, 36). Finally, cephalosporins exhibited a heterogeneous safety profile. Cefprozil was associated with higher rates of diarrhea and rash but a lower risk of abdominal pain. Conversely, cefixime was linked to an increased risk of abdominal pain, despite showing the lowest risk of vomiting. Furthermore, cefuroxime axetil demonstrated a notably lower risk of abdominal pain. These intraclass differences likely reflect variations in gastrointestinal exposure, direct mucosal irritation, and differential effects on the commensal microbiome, which may alter microbial balance and gastrointestinal tolerability (37, 38).

A key finding of this NMA is the superior clinical performance of Watch group cephalosporins (particularly cefdinir and cefprozil) compared with penicillin V and amoxicillin, the standard Access agents. These cephalosporins offer faster symptom relief and superior bacterial eradication. However, their broader antimicrobial spectrum warrants a careful stewardship approach. According to the World Health Organization AWaRe classification principles, routine use of Watch agents may accelerate antimicrobial resistance. This concern is particularly relevant given that GAS remains globally susceptible to penicillins (25, 39), which should remain first-line therapy. Accordingly, these Watch group cephalosporins should be restricted as evidence-based alternatives for specific clinical scenarios. These include patients with confirmed severe penicillin allergy, particularly those with a history of anaphylaxis or severe cutaneous adverse reactions such as Stevens-Johnson syndrome. They may also be appropriate for individuals at high risk of suppurative complications or those with first-line treatment failure. Although precise thresholds for symptom severity require further standardization by professional societies, our study provides the necessary evidence base to support these clinical protocols.

Some limitations should be considered when interpreting our findings. First, per-protocol data were utilized as most trials did not report intention-to-treat analyses. Although this approach maintains network consistency, it may slightly overestimate treatment effects. Second, data sparsity in certain nodes limited statistical power. For example, evidence for spiramycin is based on a single trial with a small sample size, which limits the generalizability and robustness of its observed superiority. Third, although subgroup analyses stratified by treatment duration addressed some variability, combining different dosages and dosing frequencies within network nodes may still introduce residual clinical heterogeneity. Fourth, zero-event cells in several safety trials reduced statistical precision, resulting in wide CIs for specific adverse events. Fifth, local inconsistency was observed in the amoxicillin-clavulanate versus azithromycin loop in late bacterial eradication. This likely reflects regional variations in macrolide resistance; however, the absence of significant global inconsistency suggests that overall network stability remains preserved. Finally, most included trials were conducted in high-income countries, limiting applicability to low- and middle-income countries (LMICs). High-cost agents such as cefdinir and cefprozil may not be available in these settings. Given that LMICs carry the greatest burden of rheumatic heart disease (28), penicillin V and amoxicillin remain the essential first-line treatments in these regions.

## 5 Conclusion

This NMA highlights the importance of strengthened antimicrobial stewardship in the treatment of GAS pharyngitis. Standard penicillins should remain the first-line therapy to minimize the antimicrobial resistance. In patients with penicillin allergy or treatment failure, cefdinir may serve as a highly effective alternative, particularly in pediatric populations. Conversely, azithromycin should be avoided due to comparatively inferior outcomes and safety concerns. This strategy aligns clinical efficacy with global stewardship goals.

## Data Availability

The original contributions presented in the study are included in the article/Supplementary material, further inquiries can be directed to the corresponding authors.

## Supplementary material

SUPPLEMENTARY FIGURE 1 Risk of bias summary for included RCTs. Review authors’ judgements about each risk of bias domain for the included studies.

SUPPLEMENTARY FIGURE 2 Funnel plots for primary and secondary outcomes. Funnel plots assessing small‐study effects for each outcome: **(A)** early bacterial eradication, **(B)** late bacterial eradication, **(C)** early clinical response, and **(D)** late clinical response, **(E)** early bacteriological recurrence, **(F)** late bacteriological recurrence, **(G)** overall adverse events, **(H)** diarrhea, **(I)** vomiting, **(J)** nausea, **(K)** abdominal pain, **(L)** rash, and **(M)** headache.
