# Supplementary figures and images for: Efficacy and safety of 24 antibiotics for group A streptococcal pharyngitis: a network meta-analysis of 64 randomized controlled trials
Source: Front Public Health. 2026 Jun 22;14:1848949. doi: 10.3389/fpubh.2026.1848949 (PMC13333768; doi:10.3389/fpubh.2026.1848949)

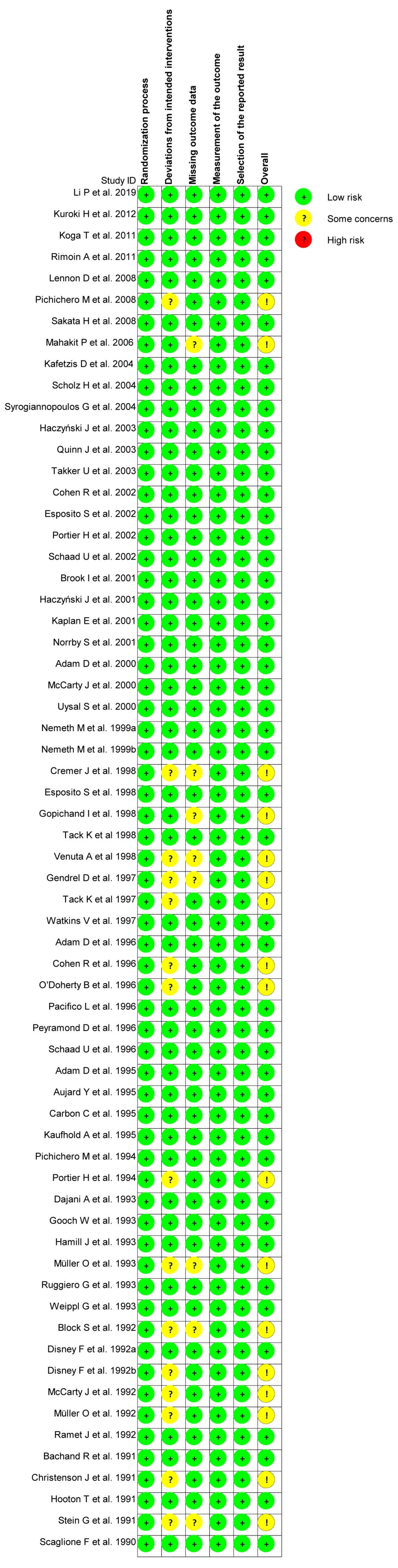

Supplement: SUPPLEMENTARY FIGURE 1 — Risk of bias summary for included RCTs. Review authors’ judgements about each risk of bias domain for the included studies. [file Image_1.tif]

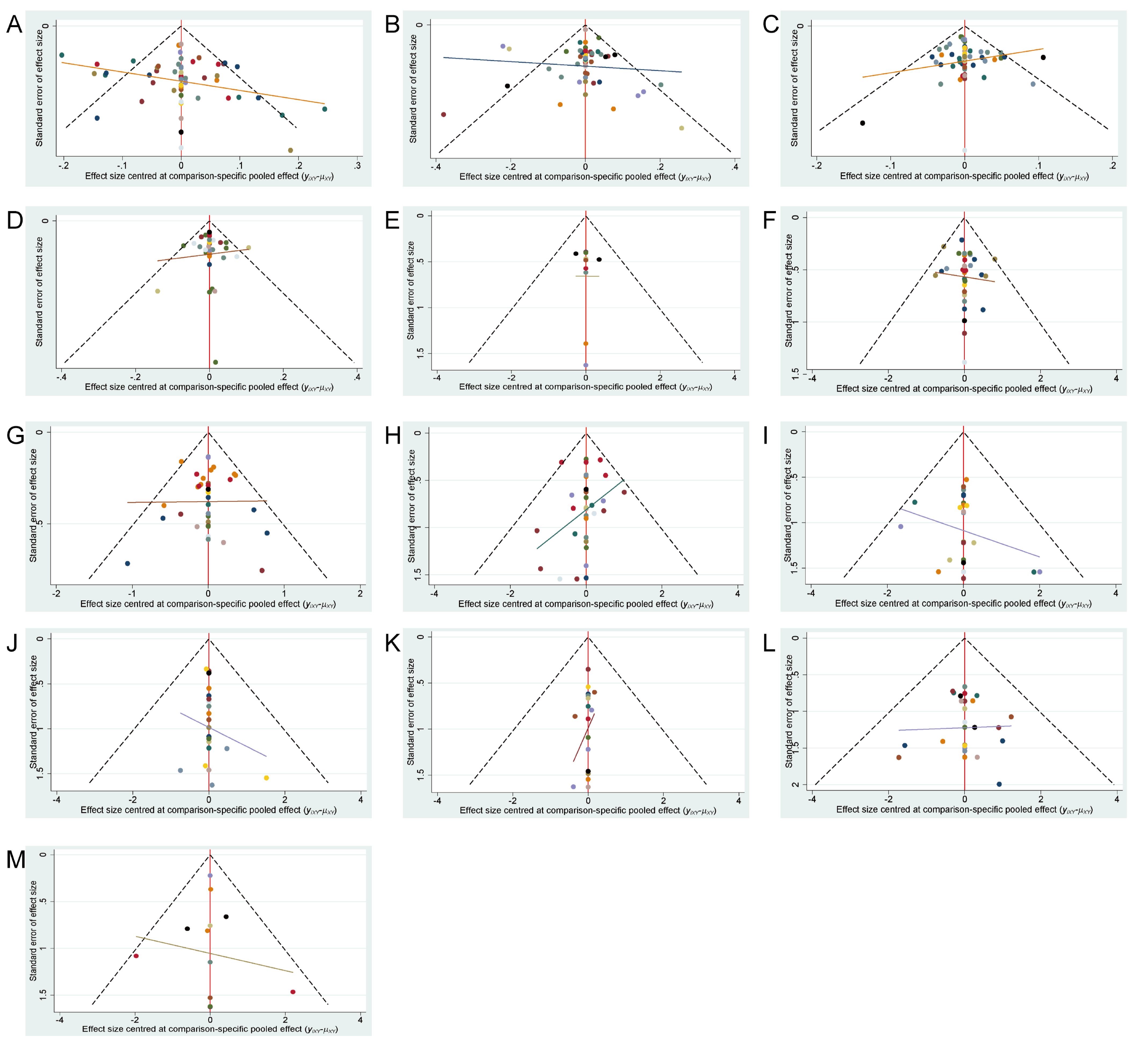

Supplement: SUPPLEMENTARY FIGURE 2 — Funnel plots for primary and secondary outcomes. Funnel plots assessing small‐study effects for each outcome: (A) early bacterial eradication, (B) late bacterial eradication, (C) early clinical response, and (D) late clinical response, (E) early bacteriological recurrence, (F) late bacteriological recurrence, (G) overall adverse events, (H) diarrhea, (I) vomiting, (J) nausea, (K) abdominal pain, (L) rash, and (M) headache. [file Image_2.tif]
