# Supplementary material for: Efficacy and safety of 24 antibiotics for group A streptococcal pharyngitis: a network meta-analysis of 64 randomized controlled trials
Source: Front Public Health. 2026 Jun 22;14:1848949. doi: 10.3389/fpubh.2026.1848949 (PMC13333768; doi:10.3389/fpubh.2026.1848949)
Supplement: Supplementary file 3 [file Supplementary_file_1.docx]

**Supplementary Appendix 1: List of studies included in the systematic review and meta-analysis**

1. Ruggiero G, Utfli R, Adinoht LE, Attanasio V, Scarano MP, Mazzone A, et al. Clinical efficacy of dirithromycin versus miocamycin in tonsillopharyngitis. *J Antimicrob Chemother*. 1993;31:S103–109. [doi: https://doi.org/10.1093/jac/31.suppl_c.103](https://doi.org/10.1093/jac/31.suppl_c.103)
2. Müller O, Wettich K. Clinical efficacy of dirithromycin in pharyngitis and tonsillitis. *J Antimicrob Chemother*. 1993;31:S97–102. [doi: https://doi.org/10.1093/jac/31.suppl_c.97](https://doi.org/10.1093/jac/31.suppl_c.97)
3. Weippl G. Multicentre comparison of azithromycin versus erythromycin in the treatment of paediatric pharyngitis or tonsillitis caused by group A streptococci. *J Antimicrob Chemother*. 1993;31:95–101. [doi: https://doi.org/10.1093/jac/31.suppl_e.95](https://doi.org/10.1093/jac/31.suppl_e.95)
4. Portier H, Chavanet P, Waldner-Combernoux A, Kisterman JP, Grey PC, Ichou F, et al. Five versus ten days treatment of streptococcal pharyngotonsillitis: a randomized controlled trial comparing cefpodoxime proxetil and phenoxymethyl penicillin. *Scand J Infect Dis*. 1994;26:59–66. [doi: https://doi.org/10.3109/00365549409008592](https://doi.org/10.3109/00365549409008592)
5. Pichichero ME. Effective short-course treatment of acute group A β-hemolytic streptococcal tonsillopharyngitis. *Arch Pediatr Adolesc Med*. 1994;148:1053–1060. [doi: https://doi.org/10.1001/archpedi.1994.02170100051010](https://doi.org/10.1001/archpedi.1994.02170100051010)
6. Adam D, Hostalek U, Tröster K. 5-day cefixime therapy for bacterial pharyngitis and/or tonsillitis: comparison with 10-day penicillin V therapy. *Infection*. 1995;23:S83–86. [doi: https://doi.org/10.1007/BF01742990](https://doi.org/10.1007/BF01742990)
7. Aujard Y, Boucot I, Brahimi N, Chiche D, Bingen E. Comparative efficacy and safety of four-day cefuroxime axetil and ten-day penicillin treatment of group A beta-hemolytic streptococcal pharyngitis in children. *Pediatr Infect Dis J*. 1995;14:295–300. [doi: https://doi.org/10.1097/00006454-199504000-00009](https://doi.org/10.1097/00006454-199504000-00009)
8. Carbon C, Chatelin A, Bingen E, Zuck P, Rio Y, Guetat F, et al. A double-blind randomized trial comparing the efficacy and safety of a 5-day course of cefotiam hexetil with that of a 10-day course of penicillin V in adult patients with pharyngitis caused by group A β-haemolytic streptococci. *J Antimicrob Chemother*. 1995;35:843–54. [doi: https://doi.org/10.1093/jac/35.6.843](https://doi.org/10.1093/jac/35.6.843)
9. Cohen R, Levy C, Doit C, Rocque FDL, Boucherat M, Fitoussi F, et al. Six-day amoxicillin vs. ten-day penicillin V therapy for group A streptococcal tonsillopharyngitis. *Pediatr Infect Dis J*. 1996;15:678–682. [doi: https://doi.org/10.1097/00006454-199608000-00008](https://doi.org/10.1097/00006454-199608000-00008)
10. Pacifico L, Scopetti F, Ranucci A, Pataracchia M, Savignoni F, Chiesa C. Comparative efficacy and safety of 3-day azithromycin and 10-day penicillin V treatment of group A beta-haemolytic streptococcal pharyngitis in children. *Antimicrob Agents Chemother*. 1996;40:1005–8. [doi: https://doi.org/10.1128/AAC.40.4.1005](https://doi.org/10.1128/AAC.40.4.1005)
11. Schaad UB, Heynen G, Swiss Tonsillopharyngitis Study Group. Evaluation of the efficacy, safety and toleration of azithromycin vs. penicillin V in the treatment of acute streptococcal pharyngitis in children: results of a multicenter, open comparative study. *Pediatr Infect Dis J*. 1996;15:791–795. [doi: https://doi.org/10.1097/00006454-199609000-00011](https://doi.org/10.1097/00006454-199609000-00011)
12. Adam D, Scholz H, Group the PS. Five days of erythromycin estolate versus ten days of penicillin V in the treatment of group A streptococcal tonsillopharyngitis in children. *Eur J Clin Microbiol Infect Dis*. 1996;15:712-717. [doi: https://doi.org/10.1007/BF01691957](https://doi.org/10.1007/BF01691957)
13. Peyramond D, Portier H, Geslin P, Cohen R. Six-day amoxicillin versus ten-day penicillin V for group A β-haemolytic streptococcal acute tonsillitis in adults: a French multicentre, open-label, randomized study. *Scand J Infect Dis*. 1996;28:497–501. [doi: https://doi.org/10.3109/00365549609037947](https://doi.org/10.3109/00365549609037947)
14. Doherty C, The Paediatric Azithromycin Study Group. Azithromycin versus penicillin V in the treatment of paediatric patients with acute streptococcal pharyngitis/tonsillitis. *Eur J Clin Microbiol Infect Dis*. 1996;15:718–724. [doi: https://doi.org/10.1007/BF01691958](https://doi.org/10.1007/BF01691958)
15. Kaufhold A, Lütticken R, Schwien U. Randomized evaluation of benzathine penicillin V twice daily versus potassium penicillin V three times daily in the treatment of group A streptococcal pharyngitis. *Eur J Clin Microbiol Infect Dis*. 1995;14:92–98. [doi: https://doi.org/10.1007/BF02111865](https://doi.org/10.1007/BF02111865)
16. Tack KJ, Hedrick JA, Rothstein E, Nemeth MA, Keyserling CH, Pichichero ME, et al. A study of 5-day cefdinir treatment for streptococcal pharyngitis in children. *Arch Pediatr Adolesc Med*. 1997;151:45–49. [doi: https://doi.org/10.1001/archpedi.1997.02170380049008](https://doi.org/10.1001/archpedi.1997.02170380049008)
17. Gendrel D, Bourillon A, Bingen E, Raymond J, Lilienthal F, Touron D. Five-day spiramycin vs seven-day penicillin V in the treatment of streptococcal tonsillitis in children. *Clin Drug Investig*. 1997;13:338–344. [doi: https://doi.org/10.2165/00044011-199713060-00006](https://doi.org/10.2165/00044011-199713060-00006)
18. Cremer J, Wallrauch C, Milatovic D, Braveny I. Azithromycin versus cefaclor in the treatment of pediatric patients with acute group A beta-hemolytic streptococcal tonsillopharyngitis. *Eur J Clin Microbiol Infect Dis*. 1998;17:235–239. [doi: https://doi.org/10.1007/BF01699979](https://doi.org/10.1007/BF01699979)
19. Watkins VS, Smietana M, Conforti PM, Sides GD, Huck W. Comparison of dirithromycin and penicillin for treatment of streptococcal pharyngitis. *Antimicrob Agents Chemother*. 1997;41:72–75. [doi: https://doi.org/10.1128/AAC.41.1.72](https://doi.org/10.1128/AAC.41.1.72)
20. Gopichand I, Williams GD, Medendorp SV, Saracusa C, Sabella C, Lampe JB, et al. Randomized, single-blinded comparative study of the efficacy of amoxicillin (40 mg/kg/day) versus standard-dose penicillin V in the treatment of group A streptococcal pharyngitis in children. *Clin Pediatr (Phila)*. 1998;37:341–346. [doi: https://doi.org/10.1177/000992289803700602](https://doi.org/10.1177/000992289803700602)
21. Esposito S, De Ritis G, D’Errico G, Noviello S, Ianniello F. Clinical comparison of cefaclor twice daily versus amoxicillin-clavulanate or erythromycin three times daily in the treatment of patients with streptococcal pharyngitis. *Clin Ther*. 1998;20:72–79. [doi: https://doi.org/10.1016/S0149-2918(98)80035-0](https://doi.org/10.1016/S0149-2918(98)80035-0)
22. Venuta A, Laudizi L, Beverelli A, Bettelli F, Milioli S, Garetti E. Azithromycin compared with clarithromycin for the treatment of streptococcal pharyngitis in children. *J Int Med Res*. 1998;26:152–158. [doi: https://doi.org/10.1177/030006059802600306](https://doi.org/10.1177/030006059802600306)
23. Tack KJ, Henry DC, Gooch WM, Brink DN, Keyserling CH, Group TCPS. Five-day cefdinir treatment for streptococcal pharyngitis. *Antimicrob Agents Chemother*. 1998;42:1073–1075. [doi: https://doi.org/10.1128/AAC.42.5.1073](https://doi.org/10.1128/AAC.42.5.1073)
24. Nemeth MA, McCarty J, Gooch WM, Henry D, Keyserling CH, Tack KJ. Comparison of cefdinir and penicillin for the treatment of streptococcal pharyngitis. *Clin Ther*. 1999;21:1873–1881. [doi: https://doi.org/10.1016/s0149-2918(00)86735-1](https://doi.org/10.1016/s0149-2918(00)86735-1)
25. Nemeth MA, Gooch WM, Hedrick J, Slosberg E, Keyserling CH, Tack KJ. Comparison of cefdinir and penicillin for the treatment of pediatric streptococcal pharyngitis. *Clin Ther*. 1999;21:1525–1532. [doi: https://doi.org/10.1016/s0149-2918(00)80007-7](https://doi.org/10.1016/s0149-2918(00)80007-7)
26. Kaplan EL, Gooch III WM, Notario GF, Craft JC. Macrolide therapy of group A streptococcal pharyngitis: 10 days of macrolide therapy (clarithromycin) is more effective in streptococcal eradication than 5 days (azithromycin). *Clin Infect Dis*. 2001;32:1798–1802. [doi: https://doi.org/10.1086/320745](https://doi.org/10.1086/320745)
27. McCarty J, Hedrick JA, Gooch WM. Clarithromycin suspension vs penicillin V suspension in children with streptococcal pharyngitis. *Adv Ther*. 2000;17:14–26. [doi: https://doi.org/10.1007/BF02868027](https://doi.org/10.1007/BF02868027)
28. Uysal S, Sancak R, Sunbul M. A comparison of the efficacy of cefuroxime axetil and intramuscular benzathine penicillin for treating streptococcal tonsillopharyngitis. *Ann Trop Paediatr*. 2000;20:199–202. [doi: https://doi.org/10.1080/02724936.2000.11748134](https://doi.org/10.1080/02724936.2000.11748134)
29. Adam D, Scholz H, Helmerking M. Comparison of short-course (5-day) cefuroxime axetil with a standard 10-day oral penicillin V regimen in the treatment of tonsillopharyngitis. *J Antimicrob Chemother*. 2000;45:23–30. [doi: https://doi.org/10.1093/jac/45.suppl_1.23](https://doi.org/10.1093/jac/45.suppl_1.23)
30. Kuroki H, Ishiwada N, Inoue N, Ishikawa N, Suzuki H, Himi K, et al. Comparison of clinical efficacy between 3-day combined clavulanate/amoxicillin preparation treatment and 10-day amoxicillin treatment in children with pharyngolaryngitis or tonsillitis. *J Infect Chemother*. 2013;19:12–9. [doi: https://doi.org/10.1007/s10156-012-0444-1](https://doi.org/10.1007/s10156-012-0444-1)
31. Brook I, Aronovitz GH, Pichichero ME. Open-label, parallel-group, multicenter, randomized study of cefprozil versus erythromycin in children with group A streptococcal pharyngitis/tonsillitis. *Clin Ther*. 2001;23:1889–1900. [doi: https://doi.org/10.1016/s0149-2918(00)89084-0](https://doi.org/10.1016/s0149-2918(00)89084-0)
32. Scholz H. Streptococcal-A tonsillopharyngitis: a 5-day course of cefuroxime axetil versus a 10-day course of penicillin V. *Chemotherapy*. 2004;50:51–4. [doi: https://doi.org/10.1159/000077286](https://doi.org/10.1159/000077286)
33. Kafetzis DA, Liapi G, Tsolia M, Aoudi H, Mathioudakis J, Paraskakis I, et al. Failure to eradicate Group A beta-haemolytic streptococci (GABHS) from the upper respiratory tract after antibiotic treatment. *Int J Antimicrob Agents*. 2004;23:67–71. [doi: https://doi.org/10.1016/j.ijantimicag.2003.05.015](https://doi.org/10.1016/j.ijantimicag.2003.05.015)
34. Portier H, Chavanet P, Gouyon JB, et al. Five-day clarithromycin modified release versus 10-day penicillin V for group A streptococcal pharyngitis: a multicenter, open-label, randomized study. *J Antimicrob Chemother*. 2002;49:337–44. [doi: https://doi.org/10.1093/jac/49.2.337](https://doi.org/10.1093/jac/49.2.337)
35. Norrby SR, Rabie WJ, Bacart P, et al. Efficacy of short-course therapy with the ketolide telithromycin compared with 10 days of penicillin V for the treatment of pharyngitis/tonsillitis. *Scand J Infect Dis*. 2001;33:883–90. [doi: https://doi.org/10.1080/00365540110077443](https://doi.org/10.1080/00365540110077443)
36. Cohen R, Reinert P, de la Rocque F, Levy C, Boucherat M, Robert M, et al. Comparison of two dosages of azithromycin for three days versus penicillin V for ten days in acute group A streptococcal tonsillopharyngitis. *Pediatr Infect Dis J*. 2002;21:297–303. [doi: https://doi.org/10.1097/00006454-200204000-00008](https://doi.org/10.1097/00006454-200204000-00008)
37. Takker U, Dzyublyk O, Busman T, Notario G. Comparison of 5 days of extended-release clarithromycin versus 10 days of penicillin V for the treatment of streptococcal pharyngitis/tonsillitis: results of a multicenter, double-blind, randomized study in adolescent and adult patients. *Curr Med Res Opin*. 2003;19:421–429. [doi: https://doi.org/10.1185/030079903125002027](https://doi.org/10.1185/030079903125002027)
38. Haczyński J, Chmielik M, Bień S, Kawalski H, Zawadzka-Głos L, Mierzwa T, et al. A comparative study of cefaclor vs amoxicillin/clavulanate in pediatric pharyngotonsillitis. *Med Sci Monit*. 2003;9:129–135.
39. Haczyński J, Bardadin J, Gryczyńska D, Gryczyński M, Gołąbek W, Kawalski H, Kaźmierczak H, Kręcicki T, Kubik P, Namysłowski G, Popiel L. A comparative study of cefaclor vs. amoxicillin/clavulanate in tonsillopharyngitis. *Med Sci Monit*. 2001;7:1016–1022.
40. Schaad UB, Kellerhals P, Altwegg M. Azithromycin versus penicillin V for treatment of acute group A streptococcal pharyngitis. *Pediatr Infect Dis J*. 2002;21:304–308. [doi: https://doi.org/10.1097/00006454-200204000-00009](https://doi.org/10.1097/00006454-200204000-00009)
41. Esposito S, Marchisio P, Bosis S, Droghetti R, Mattina R, Principi N. Comparative efficacy and safety of 5-day cefaclor and 10-day amoxycillin treatment of group A streptococcal pharyngitis in children. *Int J Antimicrob Agents*. 2002;20:28–33. [doi: https://doi.org/10.1016/s0924-8579(02)00118-8](https://doi.org/10.1016/s0924-8579(02)00118-8)
42. Quinn J, Ruoff GE, Ziter PS. Efficacy and tolerability of 5-day, once-daily telithromycin compared with 10-day, twice-daily clarithromycin for the treatment of group A beta-hemolytic streptococcal tonsillitis/pharyngitis: a multicenter, randomized, double-blind, parallel-group study. *Clin Ther*. 2003;25:422–443. [doi: https://doi.org/10.1016/S0149-2918(03)80087-5](https://doi.org/10.1016/S0149-2918(03)80087-5)
43. Syrogiannopoulos GA, Bozdogan B, Grivea IN, Ednie LM, Kritikou DI, Katopodis GD, Beratis NG, Appelbaum PC. Two dosages of clarithromycin for five days, amoxicillin/clavulanate for five days or penicillin V for ten days in acute group A streptococcal tonsillopharyngitis. *Pediatr Infect Dis J*. 2004;23:857–865. [doi: https://doi.org/10.1097/01.inf.0000138080.74674.a2](https://doi.org/10.1097/01.inf.0000138080.74674.a2)
44. Lennon DR, Farrell E, Martin DR, Stewart JM. Once-daily amoxicillin versus twice-daily penicillin V in group A β-hemolytic streptococcal pharyngitis. *Arch Dis Child*. 2008;93:474–478. [doi: https://doi.org/10.1136/adc.2006.113506](https://doi.org/10.1136/adc.2006.113506)
45. Pichichero ME, Casey JR, Block SL, Guttendorf R, Flanner H, Markowitz D, Clausen S. Pharmacodynamic analysis and clinical trial of amoxicillin sprinkle administered once daily for 7 days compared to penicillin V potassium administered four times daily for 10 days in the treatment of tonsillopharyngitis due to Streptococcus pyogenes in children. *Antimicrob Agents Chemother*. 2008;52:2512–2520. [doi: https://doi.org/10.1128/AAC.00132-07](https://doi.org/10.1128/AAC.00132-07)
46. Sakata H. Comparative study of 5-day cefcapene-pivoxil and 10-day amoxicillin or cefcapene-pivoxil for treatment of group A streptococcal pharyngitis in children. *J Infect Chemother*. 2008;14:208–212. [doi: https://doi.org/10.1007/s10156-008-0597-0](https://doi.org/10.1007/s10156-008-0597-0)
47. Koga T, Rikimaru T, Tokunaga N, Higashi T, Nakamura M, Ichikawa Y, et al. Evaluation of short-term clinical efficacy of 3-day therapy with azithromycin in comparison with 5-day cefcapene-pivoxyl for acute streptococcal tonsillopharyngitis in primary care. *J Infect Chemother*. 2011;17:499–503. [doi: https://doi.org/10.1007/s10156-010-0207-9](https://doi.org/10.1007/s10156-010-0207-9)
48. Rimoin AW, Hoff NA, Fischer Walker CL, et al. Treatment of streptococcal pharyngitis with once-daily amoxicillin versus intramuscular benzathine penicillin G in low-resource settings: a randomized controlled trial. *Clin Pediatr (Phila)*. 2011;50:535–542. [doi: https://doi.org/10.1177/0009922810394838](https://doi.org/10.1177/0009922810394838)
49. Li P, Jiang G, Shen X. Evaluation of 3-day azithromycin or 5-day cefaclor in comparison with 10-day amoxicillin for treatment of tonsillitis in children. *Can J Physiol Pharmacol*. 2019;97:939–944. [doi: https://doi.org/10.1139/cjpp-2019-0087](https://doi.org/10.1139/cjpp-2019-0087)
50. Mahakit P, Vicente JG, Butt DI, Angeli G, Bansal S, Zambrano D. Oral clindamycin 300 mg BID compared with oral amoxicillin/clavulanic acid 1 g BID in the outpatient treatment of acute recurrent pharyngotonsillitis caused by group A beta-hemolytic streptococci: an international, multicenter, randomized, investigator-blinded, prospective trial in patients between the ages of 12 and 60 years. *Clin Ther*. 2006;28:99–109. [doi: https://doi.org/10.1016/j.clinthera.2006.01.006](https://doi.org/10.1016/j.clinthera.2006.01.006)
51. Scaglione F. Comparison of the clinical and bacteriological efficacy of clarithromycin and erythromycin in the treatment of streptococcal pharyngitis. *Curr Med Res Opin*. 1990;12:25–33. [doi: https://doi.org/10.1185/03007999009111488](https://doi.org/10.1185/03007999009111488)
52. Bachand RT. A comparative study of clarithromycin and penicillin VK in the treatment of outpatients with streptococcal pharyngitis. *J Antimicrob Chemother*. 1991;27:75–82. [doi: https://doi.org/10.1093/jac/27.suppl_A.75](https://doi.org/10.1093/jac/27.suppl_A.75)
53. Christenson JC, Swenson E, Gooch WM, Herrod JN. Comparative efficacy and safety of cefprozil (BMY-28100) and cefaclor in the treatment of acute group A beta-hemolytic streptococcal pharyngitis. *Antimicrob Agents Chemother*. 1991;35:1127–1130. [doi: https://doi.org/10.1128/AAC.35.6.1127](https://doi.org/10.1128/AAC.35.6.1127)
54. Disney FA, Dillon H, Blumer JL, Dudding BA, McLinn SE, Nelson DB, et al. Cephalexin and penicillin in the treatment of group A beta-hemolytic streptococcal throat infections. *Am J Dis Child*. 1992;146:1324–1327. [doi: https://doi.org/10.1001/archpedi.1992.02160230082024](https://doi.org/10.1001/archpedi.1992.02160230082024)
55. Stein GE, Christensen S, Mummaw N. Comparative study of clarithromycin and penicillin V in the treatment of streptococcal pharyngitis. *Eur J Clin Microbiol Infect Dis*. 1991;10:949–953. [doi: https://doi.org/10.1007/BF02005450](https://doi.org/10.1007/BF02005450.)
56. Hooton TM. A comparison of azithromycin and penicillin V for the treatment of streptococcal pharyngitis. *Am J Med*. 1991;91:S23–26. [doi: https://doi.org/10.1016/0002-9343(91)90397-g](https://doi.org/10.1016/0002-9343(91)90397-g)
57. Block S. Comparative study of the effectiveness of cefixime and penicillin V for the treatment of streptococcal pharyngitis in children and adolescents. *Pediatr Infect Dis J*. 1992;11:919–925. [doi: https://doi.org/10.1097/00006454-199211110-00003](https://doi.org/10.1097/00006454-199211110-00003)
58. McCarty J. Loracarbef versus penicillin VK in the treatment of streptococcal pharyngitis and tonsillitis in an adult population. *Am J Med*. 1992;92:S74–79. [doi: https://doi.org/10.1016/0002-9343(92)90612-F](https://doi.org/10.1016/0002-9343(92)90612-F)
59. Müller O, Spirer Z, Wettich K. Loracarbef versus penicillin V in the treatment of streptococcal pharyngitis and tonsillitis. *Infection*. 1992;20:301–308. [doi: https://doi.org/10.1007/BF01710806](https://doi.org/10.1007/BF01710806)
60. Disney F. Loracarbef (LY163892) vs. penicillin VK in the treatment of streptococcal pharyngitis and tonsillitis. *Pediatr Infect Dis J*. 1992;11:S20–26. [doi: https://doi.org/10.1097/00006454-199208001-00004](https://doi.org/10.1097/00006454-199208001-00004)
61. Ramet J, Pierard D, Vandenberghe P, De Boeck K. Comparative study of cefetamet pivoxil and penicillin V in the treatment of group A beta-hemolytic streptococcal pharyngitis. *Chemotherapy*. 1992;38:33–37. [doi: https://doi.org/10.1159/000239096](https://doi.org/10.1159/000239096)
62. Gooch WM, McLinn SE, Aronovitz GH, Pichichero ME, Kumar A, Kaplan EL, et al. Efficacy of cefuroxime axetil suspension compared with that of penicillin V suspension in children with group A streptococcal pharyngitis. *Antimicrob Agents Chemother*. 1993;37:159–163. [doi: https://doi.org/10.1128/AAC.37.2.159](https://doi.org/10.1128/AAC.37.2.159)
63. Dajani AS, Kessler SL, Mendelson R, Uden DL, Todd WM. Cefpodoxime proxetil vs. penicillin V in pediatric streptococcal pharyngitis/tonsillitis. *Pediatr Infect Dis J*. 1993;12:275–279. [doi: https://doi.org/10.1097/00006454-199304000-00003](https://doi.org/10.1097/00006454-199304000-00003)
64. Hamill J. Multicentre evaluation of azithromycin and penicillin V in the treatment of acute streptococcal pharyngitis and tonsillitis in children. *J Antimicrob Chemother*. 1993;31:S89–94. [doi: https://doi.org/10.1093/jac/31.suppl_E.89](https://doi.org/10.1093/jac/31.suppl_E.89)
