# Supplementary material for: Efficacy and safety of 24 antibiotics for group A streptococcal pharyngitis: a network meta-analysis of 64 randomized controlled trials
Source: Front Public Health. 2026 Jun 22;14:1848949. doi: 10.3389/fpubh.2026.1848949 (PMC13333768; doi:10.3389/fpubh.2026.1848949)
Supplement: Supplementary file 4 [file Supplementary_file_2.doc]

**Supplementary Table 1. PRISMA checklist**

| **Section and Topic** | **Item #** | **Checklist item** | **Location where item is reported** |
| --- | --- | --- | --- |
| **TITLE** | | |  |
| Title | 1 | Identify the report as a systematic review. | Title page |
| **ABSTRACT** | | |  |
| Abstract | 2 | See the PRISMA 2020 for Abstracts checklist. | Abstract |
| **INTRODUCTION** | | |  |
| Rationale | 3 | Describe the rationale for the review in the context of existing knowledge. | Introduction |
| Objectives | 4 | Provide an explicit statement of the objective(s) or question(s) the review addresses. | Introduction |
| **METHODS** | | |  |
| Eligibility criteria | 5 | Specify the inclusion and exclusion criteria for the review and how studies were grouped for the syntheses. | Methods, Eligibility criteria |
| Information sources | 6 | Specify all databases, registers, websites, organisations, reference lists and other sources searched or consulted to identify studies. Specify the date when each source was last searched or consulted. | Methods, Search strategy |
| Search strategy | 7 | Present the full search strategies for all databases, registers and websites, including any filters and limits used. | Supplementary Table 2 |
| Selection process | 8 | Specify the methods used to decide whether a study met the inclusion criteria of the review, including how many reviewers screened each record and each report retrieved, whether they worked independently, and if applicable, details of automation tools used in the process. | Methods, Study selection |
| Data collection process | 9 | Specify the methods used to collect data from reports, including how many reviewers collected data from each report, whether they worked independently, any processes for obtaining or confirming data from study investigators, and if applicable, details of automation tools used in the process. | Methods, Data extraction |
| Data items | 10a | List and define all outcomes for which data were sought. Specify whether all results that were compatible with each outcome domain in each study were sought (e.g. for all measures, time points, analyses), and if not, the methods used to decide which results to collect. | Methods, Outcomes |
|  | 10b | List and define all other variables for which data were sought (e.g. participant and intervention characteristics, funding sources). Describe any assumptions made about any missing or unclear information. | Methods, Data extraction |
| Study risk of bias assessment | 11 | Specify the methods used to assess risk of bias in the included studies, including details of the tool(s) used, how many reviewers assessed each study and whether they worked independently, and if applicable, details of automation tools used in the process. | Methods, Risk of bias assessment |
| Effect measures | 12 | Specify for each outcome the effect measure(s) (e.g. risk ratio, mean difference) used in the synthesis or presentation of results. | Methods, Statistical analysis |
| Synthesis methods | 13a | Describe the processes used to decide which studies were eligible for each synthesis (e.g. tabulating the study intervention characteristics and comparing against the planned groups for each synthesis (item #5)). | Methods, Statistical analysis |
|  | 13b | Describe any methods required to prepare the data for presentation or synthesis, such as handling of missing summary statistics, or data conversions. | Methods, Statistical analysis |
|  | 13c | Describe any methods used to tabulate or visually display results of individual studies and syntheses. | Methods, Statistical analysis |
|  | 13d | Describe any methods used to synthesize results and provide a rationale for the choice(s). If meta-analysis was performed, describe the model(s), method(s) to identify the presence and extent of statistical heterogeneity, and software package(s) used. | Methods, Statistical analysis |
|  | 13e | Describe any methods used to explore possible causes of heterogeneity among study results (e.g. subgroup analysis, meta-regression). | Methods, Statistical analysis |
|  | 13f | Describe any sensitivity analyses conducted to assess robustness of the synthesized results. | Methods, Statistical analysis |
| Reporting bias assessment | 14 | Describe any methods used to assess risk of bias due to missing results in a synthesis (arising from reporting biases). | Methods, Publication bias |
| Certainty assessment | 15 | Describe any methods used to assess certainty (or confidence) in the body of evidence for an outcome. | Methods, Quality assessment |
| **RESULTS** | | |  |
| Study selection | 16a | Describe the results of the search and selection process, from the number of records identified in the search to the number of studies included in the review, ideally using a flow diagram. | Figure 1 |
|  | 16b | Cite studies that might appear to meet the inclusion criteria, but which were excluded, and explain why they were excluded. | N/A |
| Study characteristics | 17 | Cite each included study and present its characteristics. | Table 1 |
| Risk of bias in studies | 18 | Present assessments of risk of bias for each included study. | Supplementary figure 1 |
| Results of individual studies | 19 | For all outcomes, present, for each study: (a) summary statistics for each group (where appropriate) and (b) an effect estimate and its precision (e.g. confidence/credible interval), ideally using structured tables or plots. | Table 2-5, Supplementary Table 3-11 |
| Results of syntheses | 20a | For each synthesis, briefly summarise the characteristics and risk of bias among contributing studies. | Results, Study characteristics |
|  | 20b | Present results of all statistical syntheses conducted. If meta-analysis was done, present for each the summary estimate and its precision (e.g. confidence/credible interval) and measures of statistical heterogeneity. If comparing groups, describe the direction of the effect. | Results, Network geometry |
|  | 20c | Present results of all investigations of possible causes of heterogeneity among study results. | Results, Heterogeneity and inconsistency |
|  | 20d | Present results of all sensitivity analyses conducted to assess the robustness of the synthesized results. | Results, Sensitivity analysis |
| Reporting biases | 21 | Present assessments of risk of bias due to missing results (arising from reporting biases) for each synthesis assessed. | Results, Publication bias |
| Certainty of evidence | 22 | Present assessments of certainty (or confidence) in the body of evidence for each outcome assessed. | Results, Risk of bias |
| **DISCUSSION** | | |  |
| Discussion | 23a | Provide a general interpretation of the results in the context of other evidence. | Discussion |
|  | 23b | Discuss any limitations of the evidence included in the review. | Discussion, Limitations |
|  | 23c | Discuss any limitations of the review processes used. | Discussion, Limitations |
|  | 23d | Discuss implications of the results for practice, policy, and future research. | Conclusion |
| **OTHER INFORMATION** | | |  |
| Registration and protocol | 24a | Provide registration information for the review, including register name and registration number, or state that the review was not registered. | Abstract; Methods |
|  | 24b | Indicate where the review protocol can be accessed, or state that a protocol was not prepared. | Abstract; Methods |
|  | 24c | Describe and explain any amendments to information provided at registration or in the protocol. | N/A |
| Support | 25 | Describe sources of financial or non-financial support for the review, and the role of the funders or sponsors in the review. | Funding |
| Competing interests | 26 | Declare any competing interests of review authors. | Conflict of Interest |
| Availability of data, code and other materials | 27 | Report which of the following are publicly available and where they can be found: template data collection forms; data extracted from included studies; data used for all analyses; analytic code; any other materials used in the review. | Data Availability Statement |

**Supplementary Table 2. Search strategy for PubMed**

| **Search block title** | **Search terms used in PubMed** |
| --- | --- |
| **Streptococcal Infections** | "Streptococcal Infections"[Title/Abstract] OR "infection streptococcal"[Title/Abstract] OR "streptococcal infection"[Title/Abstract] OR "infections streptococcal"[Title/Abstract] OR "group a streptococcal infections"[Title/Abstract] OR "group a streptococcal infection"[Title/Abstract] OR "group a strep infection"[Title/Abstract] OR "Streptococcal Infections"[MeSH Terms] |
| **Pharyngitis** | "Tonsillitis"[Title/Abstract] OR ("Pharyngitis"[MeSH Terms] OR ("Pharyngitides"[Title/Abstract] OR "sore throat"[Title/Abstract] OR "sore throats"[Title/Abstract] OR "throat sore"[Title/Abstract] OR "Pharyngitis"[Title/Abstract])))) |
| **Therapy** | ((("therapy" [Subheading]) OR ((disease management [Title/Abstract]) OR (treatment [Title/Abstract]))) OR (therapy [Title/Abstract])) |

**Supplementary Table 3. League table of pairwise comparisons for early bacterial eradication in the pediatric subgroup**

| AMC |  |  |  |  |  |  |  |  |  |  |  |  |  |  |  |  |  |
| --- | --- | --- | --- | --- | --- | --- | --- | --- | --- | --- | --- | --- | --- | --- | --- | --- | --- |
| 0.84 (0.28, 2.49) | Amoxicillin |  |  |  |  |  |  |  |  |  |  |  |  |  |  |  |  |
| 1.38 (0.42, 4.35) | 1.66 (0.66, 3.96) | Azithromycin |  |  |  |  |  |  |  |  |  |  |  |  |  |  |  |
| 0.63 (0.06, 6.93) | 0.76 (0.08, 7.49) | 0.46 (0.05, 4.52) | BPG |  |  |  |  |  |  |  |  |  |  |  |  |  |  |
| 0.97 (0.13, 7.36) | 1.16 (0.18, 7.81) | 0.7 (0.11, 4.65) | 1.54 (0.1, 23.5) | BPV |  |  |  |  |  |  |  |  |  |  |  |  |  |
| 0.7 (0.2, 2.31) | 0.83 (0.31, 2.2) | 0.5 (0.19, 1.36) | 1.1 (0.1, 11.3) | 0.72 (0.1, 5.14) | Cefaclor |  |  |  |  |  |  |  |  |  |  |  |  |
| 0.61 (0.06, 5.7) | 0.73 (0.1, 5.1) | 0.44 (0.05, 3.85) | 0.96 (0.05, 18.77) | 0.62 (0.04, 9.38) | 0.87 (0.1, 7.77) | CefcapenePivoxil | |  |  |  |  |  |  |  |  |  |  |
| **0.17 (0.04, 0.79)** | **0.2 (0.05, 0.79)** | **0.12 (0.03, 0.47)** | 0.26 (0.02, 2.87) | 0.17 (0.02, 1.34) | 0.24 (0.05, 1.1) | 0.27 (0.03, 3) | Cefdinir |  |  |  |  |  |  |  |  |  |  |
| 0.5 (0.05, 4.96) | 0.6 (0.07, 5.26) | 0.36 (0.04, 3.15) | 0.78 (0.04, 14.67) | 0.51 (0.03, 7.47) | 0.71 (0.07, 6.93) | 0.82 (0.04, 15.5) | 2.99 (0.28, 31.34) | CefetametPivoxil | |  |  |  |  |  |  |  |  |
| 0.53 (0.1, 2.78) | 0.63 (0.14, 2.81) | 0.38 (0.09, 1.67) | 0.83 (0.07, 9.84) | 0.54 (0.06, 4.66) | 0.76 (0.14, 3.86) | 0.87 (0.07, 10.22) | 3.2 (0.54, 17.44) | 1.06 (0.09, 12.09) | Cefixime |  |  |  |  |  |  |  |  |
| 0.29 (0.06, 1.42) | 0.35 (0.09, 1.41) | **0.21 (0.06, 0.84)** | 0.46 (0.04, 5.15) | 0.3 (0.04, 2.39) | 0.42 (0.09, 1.96) | 0.48 (0.04, 5.39) | 1.75 (0.35, 8.85) | 0.58 (0.06, 6.3) | 0.55 (0.1, 3.28) | CefpodoximeProxetil | |  |  |  |  |  |  |
| 0.18 (0.02, 1.63) | 0.21 (0.02, 1.89) | 0.13 (0.01, 1.12) | 0.28 (0.01, 5.53) | 0.18 (0.01, 2.73) | 0.25 (0.02, 2.3) | 0.29 (0.01, 5.49) | 1.07 (0.08, 11.66) | 0.35 (0.02, 6.77) | 0.33 (0.02, 4.13) | 0.61 (0.05, 6.72) | Cefprozil |  |  |  |  |  |  |
| 0.55 (0.14, 2.29) | 0.66 (0.21, 2.22) | 0.4 (0.13, 1.32) | 0.87 (0.13, 5.95) | 0.57 (0.08, 4.1) | 0.79 (0.21, 3.16) | 0.91 (0.09, 9.35) | 3.33 (0.79, 14.45) | 1.11 (0.12, 10.83) | 1.04 (0.22, 5.41) | 1.9 (0.44, 8.45) | 3.12 (0.32, 36.98) | CefuroximeAxetil | |  |  |  |  |
| 0.32 (0.06, 1.83) | 0.38 (0.09, 1.88) | 0.23 (0.06, 1.02) | 0.5 (0.04, 6.6) | 0.33 (0.04, 3.11) | 0.46 (0.1, 2.49) | 0.53 (0.05, 6.83) | 1.92 (0.35, 11.78) | 0.64 (0.06, 8.07) | 0.6 (0.1, 4.35) | 1.1 (0.2, 6.86) | 1.81 (0.16, 26.37) | 0.58 (0.12, 3.08) | Clarithromycin |  |  |  |  |
| 1.4 (0.37, 4.93) | 1.68 (0.47, 5.6) | 1.01 (0.31, 3.26) | 2.22 (0.19, 23.54) | 1.44 (0.18, 10.84) | 2.01 (0.57, 6.86) | 2.31 (0.23, 23.18) | **8.5 (1.64, 39.96)** | 2.82 (0.27, 28.93) | 2.66 (0.47, 14.6) | 4.85 (0.92, 23.12) | **7.92 (1.27, 55.13)** | 2.55 (0.56, 10.19) | 4.4 (0.71, 22.29) | Erythromycin |  |  |  |
| 0.58 (0.08, 4.23) | 0.69 (0.11, 4.43) | 0.42 (0.07, 2.66) | 0.91 (0.06, 13.71) | 0.59 (0.05, 6.61) | 0.83 (0.12, 5.93) | 0.95 (0.07, 14.16) | 3.48 (0.46, 26.44) | 1.16 (0.08, 16.8) | 1.09 (0.13, 9.55) | 1.99 (0.25, 15.29) | 3.28 (0.22, 55.47) | 1.05 (0.15, 7.03) | 1.81 (0.2, 14.64) | 0.41 (0.06, 3.22) | Loracarbef |  |  |
| 0.82 (0.29, 2.41) | 0.99 (0.47, 2.14) | 0.59 (0.31, 1.23) | 1.31 (0.15, 10.94) | 0.85 (0.15, 4.78) | 1.18 (0.45, 3.26) | 1.36 (0.17, 11.44) | **4.98 (1.61, 15.54)** | 1.66 (0.22, 13.25) | 1.56 (0.43, 6.01) | 2.84 (0.89, 9.05) | 4.67 (0.56, 45.32) | 1.5 (0.59, 3.7) | 2.59 (0.64, 9.38) | 0.59 (0.2, 1.88) | 1.43 (0.27, 7.77) | PenicillinV |  |
| 1.92 (0.27, 14.15) | 2.3 (0.37, 14.95) | 1.39 (0.24, 8.95) | 3.04 (0.2, 45.62) | 1.97 (0.18, 22.17) | 2.77 (0.4, 20.05) | 3.17 (0.22, 48.57) | **11.63 (1.55, 88.88)** | 3.88 (0.28, 57.54) | 3.64 (0.45, 32.31) | 6.62 (0.86, 51.66) | 10.92 (0.74, 187.41) | 3.48 (0.51, 23.92) | 6.06 (0.67, 49.41) | 1.37 (0.19, 11.02) | 3.36 (0.31, 36.45) | 2.33 (0.43, 12.7) | Spiramycin |

Values are presented as odds ratios (95% confidence intervals). Penicillin V is used as reference. Statistically significant results are highlighted in bold. Abbreviations: AMC, amoxicillin-clavulanate; BPG, Benzathine Penicillin G; BPV, Benzathine Penicillin V.

**Supplementary Table 4. League table of pairwise comparisons for early bacterial eradication in the adult subgroup**

| Amoxicillin |  |  |  |  |  |  |  |  |
| --- | --- | --- | --- | --- | --- | --- | --- | --- |
| 2.1 (0.38, 11.36) | Azithromycin |  |  |  |  |  |  |  |
| 0.54 (0.05, 5.03) | 0.26 (0.03, 2.1) | CefpodoximeProxetil |  |  |  |  |  |  |
| 0.82 (0.17, 3.64) | 0.39 (0.14, 1) | 1.51 (0.22, 11.45) | Clarithromycin |  |  |  |  |  |
| 1.34 (0.24, 7.8) | 0.64 (0.16, 2.76) | 2.5 (0.31, 22.85) | 1.66 (0.53, 5.54) | Dirithromycin |  |  |  |  |
| 1.24 (0.21, 8.29) | 0.58 (0.14, 2.85) | 2.3 (0.26, 23.47) | 1.53 (0.49, 5.58) | 0.92 (0.33, 2.72) | Erythromycin |  |  |  |
| 0.32 (0.05, 2.11) | **0.15 (0.03, 0.81)** | 0.59 (0.06, 5.96) | 0.4 (0.08, 1.76) | 0.24 (0.04, 1.26) | 0.26 (0.04, 1.46) | Loracarbef |  |  |
| 3.74 (0.12, 185.79) | 1.78 (0.07, 79.44) | 7.1 (0.18, 419.94) | 4.58 (0.2, 187.22) | 2.72 (0.15, 93.77) | 2.97 (0.14, 115.06) | 11.75 (0.41, 599.75) | Miocamycin |  |
| 0.91 (0.23, 3.48) | 0.43 (0.15, 1.19) | 1.67 (0.28, 11.3) | 1.12 (0.57, 2.26) | 0.68 (0.22, 1.93) | 0.74 (0.2, 2.35) | 2.82 (0.76, 11.66) | 0.25 (0.01, 5.52) | PenicillinV |

Values are presented as odds ratios (95% confidence intervals). Penicillin V is used as reference. Statistically significant results are highlighted in bold.

**Supplementary Table 5. League table of pairwise comparisons for early bacterial eradication stratified by treatment duration**

| AMC10d |  |  |  |  |  |  |  |  |  |  |  |  |  |  |  |  |  |  |  |  |  |  |  |  |  |  |  |  |  |  |  |
| --- | --- | --- | --- | --- | --- | --- | --- | --- | --- | --- | --- | --- | --- | --- | --- | --- | --- | --- | --- | --- | --- | --- | --- | --- | --- | --- | --- | --- | --- | --- | --- |
| 10.7 (0.62, 218.43) | AMC3d |  |  |  |  |  |  |  |  |  |  |  |  |  |  |  |  |  |  |  |  |  |  |  |  |  |  |  |  |  |  |
| 1.69 (0.14, 24.25) | 0.16 (0.01, 2.23) | AMC5d |  |  |  |  |  |  |  |  |  |  |  |  |  |  |  |  |  |  |  |  |  |  |  |  |  |  |  |  |  |
| 3.27 (0.35, 35.83) | 0.31 (0.05, 1.8) | 1.92 (0.27, 14.27) | Amoxicillin10d | |  |  |  |  |  |  |  |  |  |  |  |  |  |  |  |  |  |  |  |  |  |  |  |  |  |  |  |
| 2.03 (0.2, 23.26) | 0.19 (0.02, 2.16) | 1.2 (0.17, 8.58) | 0.62 (0.11, 3.28) | Amoxicillin6d | |  |  |  |  |  |  |  |  |  |  |  |  |  |  |  |  |  |  |  |  |  |  |  |  |  |  |
| 2.04 (0.17, 27.88) | 0.19 (0.01, 2.58) | 1.21 (0.14, 10.55) | 0.63 (0.09, 4.21) | 1 (0.15, 6.77) | Amoxicillin7d | |  |  |  |  |  |  |  |  |  |  |  |  |  |  |  |  |  |  |  |  |  |  |  |  |  |
| 3.34 (0.48, 25.29) | 0.31 (0.03, 2.57) | 1.98 (0.33, 10.78) | 1.03 (0.28, 3.32) | 1.65 (0.4, 6.28) | 1.64 (0.3, 8.23) | Azithromycin3d | |  |  |  |  |  |  |  |  |  |  |  |  |  |  |  |  |  |  |  |  |  |  |  |  |
| 4.71 (0.47, 50.4) | 0.44 (0.03, 5.44) | 2.77 (0.32, 22.8) | 1.43 (0.23, 8.58) | 2.3 (0.36, 14.14) | 2.3 (0.29, 17.46) | 1.4 (0.32, 6.33) | Azithromycin5d | |  |  |  |  |  |  |  |  |  |  |  |  |  |  |  |  |  |  |  |  |  |  |  |
| 2.14 (0.16, 32.53) | 0.2 (0.01, 2.97) | 1.26 (0.13, 12.69) | 0.65 (0.08, 5.06) | 1.05 (0.14, 8.16) | 1.04 (0.11, 9.96) | 0.64 (0.11, 4.11) | 0.45 (0.05, 4.17) | BPV10d |  |  |  |  |  |  |  |  |  |  |  |  |  |  |  |  |  |  |  |  |  |  |  |
| 0.9 (0.16, 5.3) | 0.08 (0.01, 1.03) | 0.53 (0.05, 4.68) | 0.28 (0.04, 1.69) | 0.44 (0.06, 2.96) | 0.44 (0.05, 3.64) | 0.27 (0.06, 1.1) | 0.19 (0.03, 1.32) | 0.42 (0.04, 3.91) | Cefaclor10d | |  |  |  |  |  |  |  |  |  |  |  |  |  |  |  |  |  |  |  |  |  |
| 3.44 (0.31, 42.82) | 0.32 (0.04, 2.72) | 2.02 (0.23, 17.9) | 1.05 (0.31, 3.44) | 1.68 (0.25, 11.32) | 1.67 (0.2, 14) | 1.02 (0.24, 4.64) | 0.73 (0.1, 5.58) | 1.61 (0.17, 15.32) | 3.81 (0.52, 31.02) | Cefaclor5d | |  |  |  |  |  |  |  |  |  |  |  |  |  |  |  |  |  |  |  |  |
| 2.35 (0.12, 50.68) | 0.22 (0.02, 2.93) | 1.38 (0.09, 21.76) | 0.72 (0.1, 4.88) | 1.15 (0.09, 14.8) | 1.14 (0.08, 17.54) | 0.7 (0.07, 7.07) | 0.5 (0.04, 7.24) | 1.1 (0.07, 18.46) | 2.61 (0.19, 40.62) | 0.68 (0.07, 6.56) | CefcapenePivoxil5d | |  |  |  |  |  |  |  |  |  |  |  |  |  |  |  |  |  |  |  |
| 0.45 (0.05, 4.99) | **0.04 (0, 0.46)** | 0.27 (0.04, 1.84) | **0.14 (0.03, 0.69)** | 0.22 (0.04, 1.11) | 0.22 (0.03, 1.42) | **0.14 (0.04, 0.52)** | **0.1 (0.02, 0.59)** | 0.21 (0.03, 1.57) | 0.5 (0.08, 3.61) | **0.13 (0.02, 0.84)** | 0.19 (0.02, 2.39) | Cefdinir10d |  |  |  |  |  |  |  |  |  |  |  |  |  |  |  |  |  |  |  |
| 0.75 (0.08, 8.34) | **0.07 (0.01, 0.77)** | 0.44 (0.06, 3) | 0.23 (0.04, 1.15) | 0.37 (0.07, 1.81) | 0.37 (0.06, 2.33) | **0.22 (0.06, 0.85)** | **0.16 (0.03, 0.96)** | 0.35 (0.05, 2.58) | 0.83 (0.13, 5.98) | 0.22 (0.03, 1.38) | 0.32 (0.03, 3.93) | 1.66 (0.35, 7.84) | Cefdinir5d | |  |  |  |  |  |  |  |  |  |  |  |  |  |  |  |  |  |
| 1.08 (0.06, 20.24) | 0.1 (0.01, 1.87) | 0.64 (0.05, 8.08) | 0.33 (0.03, 3.31) | 0.53 (0.05, 5.39) | 0.53 (0.04, 6.29) | 0.33 (0.04, 2.74) | 0.23 (0.02, 2.67) | 0.51 (0.04, 6.79) | 1.21 (0.1, 15.77) | 0.32 (0.03, 3.8) | 0.46 (0.02, 9.49) | 2.41 (0.24, 23.3) | 1.45 (0.14, 13.88) | CefetametPivoxil7d | |  |  |  |  |  |  |  |  |  |  |  |  |  |  |  |  |
| 0.34 (0.02, 6.13) | **0.03 (0, 0.57**) | 0.2 (0.01, 2.44) | 0.1 (0.01, 1) | 0.17 (0.01, 1.62) | 0.17 (0.01, 1.92) | **0.1 (0.01, 0.82)** | **0.07 (0.01, 0.81)** | 0.16 (0.01, 2.08) | 0.38 (0.03, 4.77) | 0.1 (0.01, 1.14) | 0.14 (0.01, 2.87) | 0.75 (0.07, 6.98) | 0.45 (0.04, 4.15) | 0.31 (0.02, 5.23) | Cefixime10d | |  |  |  |  |  |  |  |  |  |  |  |  |  |  |  |
| 2.91 (0.21, 46.16) | 0.27 (0.02, 4.22) | 1.72 (0.16, 17.61) | 0.89 (0.11, 7.21) | 1.43 (0.18, 11.63) | 1.42 (0.14, 13.94) | 0.87 (0.14, 5.91) | 0.62 (0.07, 5.98) | 1.36 (0.12, 15.21) | 3.23 (0.33, 35.51) | 0.85 (0.08, 8.34) | 1.24 (0.07, 21.57) | 6.42 (0.83, 51.1) | 3.88 (0.51, 30.11) | 2.67 (0.19, 39.25) | 8.59 (0.63, 132.48) | Cefixime5d | |  |  |  |  |  |  |  |  |  |  |  |  |  |  |
| 0.57 (0.05, 8.11) | **0.05 (0, 0.75)** | 0.34 (0.04, 3.15) | 0.18 (0.02, 1.24) | 0.28 (0.04, 2) | 0.28 (0.03, 2.45) | 0.17 (0.03, 1) | 0.12 (0.02, 1.04) | 0.27 (0.03, 2.67) | 0.64 (0.07, 6.18) | 0.17 (0.02, 1.46) | 0.25 (0.02, 3.87) | 1.27 (0.19, 8.74) | 0.77 (0.11, 5.19) | 0.53 (0.04, 6.92) | 1.69 (0.14, 24.13) | 0.2 (0.02, 2.06) | CefpodoximeProxetil10d | | |  |  |  |  |  |  |  |  |  |  |  |  |
| 0.82 (0.08, 9.92) | **0.08 (0.01, 0.92)** | 0.48 (0.06, 3.72) | 0.25 (0.04, 1.42) | 0.4 (0.07, 2.29) | 0.4 (0.06, 2.87) | 0.24 (0.06, 1.1) | 0.17 (0.03, 1.19) | 0.39 (0.05, 3.17) | 0.91 (0.13, 7.28) | 0.24 (0.03, 1.69) | 0.35 (0.03, 4.69) | 1.81 (0.34, 9.92) | 1.09 (0.2, 5.89) | 0.75 (0.07, 8.4) | 2.42 (0.24, 28.62) | 0.28 (0.03, 2.43) | 1.43 (0.19, 10.92) | CefpodoximeProxetil5d | | |  |  |  |  |  |  |  |  |  |  |  |
| 0.36 (0.03, 4.26) | **0.03 (0, 0.72)** | 0.21 (0.01, 3.43) | 0.11 (0.01, 1.36) | 0.18 (0.01, 2.34) | 0.18 (0.01, 2.71) | 0.11 (0.01, 1.03) | **0.08 (0.01, 0.96)** | 0.17 (0.01, 2.84) | 0.4 (0.04, 3.99) | 0.11 (0.01, 1.48) | 0.15 (0.01, 3.64) | 0.8 (0.05, 10.13) | 0.49 (0.03, 6) | 0.33 (0.01, 7.04) | 1.07 (0.04, 24.56) | 0.12 (0.01, 2.22) | 0.63 (0.03, 10.05) | 0.44 (0.03, 6.14) | Cefprozil10d | |  |  |  |  |  |  |  |  |  |  |  |
| 1.77 (0.13, 26.72) | 0.17 (0.01, 2.48) | 1.04 (0.1, 10.29) | 0.54 (0.07, 4.15) | 0.87 (0.11, 6.59) | 0.86 (0.09, 8.07) | 0.53 (0.09, 3.35) | 0.38 (0.04, 3.38) | 0.83 (0.08, 8.78) | 1.97 (0.21, 20.29) | 0.52 (0.05, 4.78) | 0.76 (0.04, 12.69) | 3.91 (0.53, 28.79) | 2.35 (0.32, 17.19) | 1.62 (0.12, 22.84) | 5.25 (0.4, 76.79) | 0.61 (0.05, 6.71) | 3.08 (0.31, 30.46) | 2.16 (0.26, 17.44) | 4.89 (0.28, 98.95) | CefuroximeAxetil4d | |  |  |  |  |  |  |  |  |  |  |
| 1.06 (0.11, 11.46) | 0.1 (0.01, 1.06) | 0.62 (0.09, 4.15) | 0.32 (0.06, 1.57) | 0.52 (0.11, 2.48) | 0.52 (0.08, 3.19) | 0.32 (0.09, 1.17) | 0.23 (0.04, 1.33) | 0.5 (0.07, 3.57) | 1.17 (0.19, 8.13) | 0.31 (0.05, 1.9) | 0.45 (0.04, 5.45) | 2.34 (0.51, 10.81) | 1.42 (0.31, 6.32) | 0.97 (0.1, 9.63) | 3.12 (0.35, 33.08) | 0.36 (0.05, 2.73) | 1.85 (0.28, 12.1) | 1.29 (0.25, 6.75) | 2.91 (0.24, 43.85) | 0.6 (0.08, 4.32) | CefuroximeAxetil5d | |  |  |  |  |  |  |  |  |  |
| 1.92 (0.24, 15.9) | 0.18 (0.01, 1.93) | 1.13 (0.14, 8.1) | 0.59 (0.1, 2.91) | 0.94 (0.17, 4.96) | 0.94 (0.13, 6.2) | 0.57 (0.16, 1.93) | 0.41 (0.1, 1.51) | 0.9 (0.11, 6.89) | 2.13 (0.39, 11.85) | 0.56 (0.08, 3.45) | 0.82 (0.06, 9.91) | 4.24 (0.77, 21.66) | 2.56 (0.47, 12.87) | 1.76 (0.16, 18.56) | 5.65 (0.54, 63.35) | 0.66 (0.07, 5.3) | 3.35 (0.43, 23.45) | 2.34 (0.38, 13.45) | 5.29 (0.52, 60.07) | 1.08 (0.13, 8.32) | 1.82 (0.34, 8.67) | Clarithromycin10d | |  |  |  |  |  |  |  |  |
| 0.96 (0.11, 10.18) | **0.09 (0.01, 0.94)** | 0.57 (0.09, 3.58) | 0.3 (0.06, 1.36) | 0.48 (0.11, 2.13) | 0.47 (0.08, 2.78) | **0.29 (0.09, 0.98)** | 0.21 (0.04, 1.16) | 0.45 (0.07, 3.1) | 1.07 (0.18, 7.13) | 0.28 (0.05, 1.66) | 0.41 (0.04, 4.91) | 2.13 (0.51, 9.27) | 1.29 (0.31, 5.53) | 0.89 (0.1, 8.53) | 2.85 (0.33, 29.33) | 0.33 (0.05, 2.42) | 1.68 (0.27, 10.6) | 1.18 (0.24, 5.83) | 2.66 (0.23, 39.2) | 0.55 (0.08, 3.76) | 0.91 (0.23, 3.78) | 0.5 (0.11, 2.54) | Clarithromycin5d | |  |  |  |  |  |  |  |
| 0.34 (0.05, 1.96) | **0.03 (0, 0.88)** | 0.2 (0.01, 4.31) | 0.1 (0.01, 1.77) | 0.17 (0.01, 3) | 0.17 (0.01, 3.44) | 0.1 (0.01, 1.38) | 0.07 (0, 1.29) | 0.16 (0.01, 3.62) | 0.38 (0.03, 4.49) | 0.1 (0, 1.91) | 0.14 (0, 4.43) | 0.74 (0.04, 13.25) | 0.45 (0.02, 7.79) | 0.31 (0.01, 8.73) | 1 (0.03, 29.43) | 0.12 (0, 2.75) | 0.59 (0.02, 12.72) | 0.41 (0.02, 7.73) | 0.93 (0.04, 20.79) | 0.19 (0.01, 4.34) | 0.32 (0.02, 5.52) | 0.18 (0.01, 2.7) | 0.35 (0.02, 5.72) | Clindamycin10d | |  |  |  |  |  |  |
| 1.88 (0.14, 28) | 0.18 (0.01, 2.63) | 1.11 (0.11, 10.88) | 0.57 (0.07, 4.4) | 0.92 (0.12, 6.99) | 0.92 (0.1, 8.46) | 0.56 (0.09, 3.54) | 0.4 (0.05, 3.6) | 0.88 (0.08, 9.2) | 2.08 (0.22, 21.45) | 0.55 (0.06, 5.08) | 0.8 (0.05, 13.31) | 4.15 (0.57, 30.8) | 2.51 (0.34, 18.16) | 1.73 (0.13, 23.89) | 5.55 (0.43, 81.98) | 0.65 (0.06, 7.03) | 3.27 (0.33, 32.04) | 2.29 (0.28, 18.61) | 5.16 (0.31, 104.67) | 1.06 (0.1, 11.21) | 1.77 (0.25, 12.62) | 0.98 (0.13, 8.09) | 1.94 (0.28, 12.98) | 5.59 (0.24, 145.41) | Dirithromycin10d | |  |  |  |  |  |
| 2.9 (0.54, 15.61) | 0.27 (0.02, 3.26) | 1.71 (0.18, 14.3) | 0.89 (0.13, 5.19) | 1.43 (0.2, 9.12) | 1.42 (0.16, 10.96) | 0.86 (0.21, 3.43) | 0.62 (0.1, 3.67) | 1.36 (0.13, 12.11) | 3.21 (0.76, 13.41) | 0.84 (0.11, 5.97) | 1.24 (0.08, 16.8) | 6.41 (0.92, 39.3) | 3.88 (0.56, 23.19) | 2.66 (0.21, 31.78) | 8.55 (0.69, 110.2) | 1 (0.09, 9.3) | 5.06 (0.54, 41.75) | 3.54 (0.45, 24.27) | 7.93 (1.33, 54.55) | 1.64 (0.16, 14.58) | 2.74 (0.41, 15.91) | 1.51 (0.34, 6.52) | 3.01 (0.47, 16.55) | 8.58 (0.75, 101.91) | 1.54 (0.15, 13.68) | Erythromycin10d | |  |  |  |  |
| 2.67 (0.2, 39.38) | 0.25 (0.02, 3.63) | 1.56 (0.16, 15.41) | 0.81 (0.1, 6.16) | 1.31 (0.17, 9.78) | 1.3 (0.14, 11.88) | 0.79 (0.14, 4.94) | 0.57 (0.07, 5.04) | 1.25 (0.12, 13.12) | 2.95 (0.32, 29.92) | 0.77 (0.08, 7.11) | 1.13 (0.07, 18.73) | 5.86 (0.81, 42.35) | 3.54 (0.49, 25.76) | 2.44 (0.19, 33.46) | 7.88 (0.62, 113.94) | 0.92 (0.08, 9.85) | 4.63 (0.48, 44.79) | 3.25 (0.4, 25.66) | 7.35 (0.44, 143.77) | 1.49 (0.14, 15.87) | 2.52 (0.36, 17.68) | 1.39 (0.18, 11.26) | 2.75 (0.41, 18.1) | 7.89 (0.35, 208.03) | 1.42 (0.14, 14.68) | 0.92 (0.1, 9.1) | Erythromycin5d | |  |  |  |
| 1.62 (0.16, 18.85) | 0.15 (0.01, 1.74) | 0.95 (0.13, 7.04) | 0.5 (0.09, 2.69) | 0.79 (0.15, 4.27) | 0.79 (0.12, 5.39) | 0.48 (0.12, 2.03) | 0.35 (0.05, 2.24) | 0.76 (0.1, 5.98) | 1.8 (0.26, 13.72) | 0.47 (0.07, 3.23) | 0.69 (0.05, 9.01) | 3.57 (0.71, 18.57) | 2.16 (0.43, 10.98) | 1.49 (0.15, 15.91) | 4.76 (0.49, 55.17) | 0.56 (0.07, 4.6) | 2.81 (0.39, 20.5) | 1.97 (0.35, 11.56) | 4.47 (0.34, 71.94) | 0.92 (0.12, 7.19) | 1.53 (0.32, 7.56) | 0.84 (0.16, 4.99) | 1.67 (0.36, 7.67) | 4.83 (0.26, 103.8) | 0.86 (0.11, 6.74) | 0.56 (0.09, 4.16) | 0.61 (0.08, 4.69) | Loracarbef10d | |  |  |
| 5.32 (0.1, 512.21) | 0.5 (0.01, 49.15) | 3.11 (0.07, 241.14) | 1.61 (0.04, 112.43) | 2.59 (0.07, 176.41) | 2.59 (0.06, 195.15) | 1.57 (0.05, 99.11) | 1.13 (0.03, 82.54) | 2.49 (0.05, 194.23) | 5.9 (0.14, 458.45) | 1.54 (0.03, 118.4) | 2.28 (0.04, 237.67) | 11.67 (0.31, 772.6) | 7.04 (0.19, 466.55) | 4.92 (0.09, 457) | 16 (0.29, 1493.09) | 1.83 (0.04, 151.05) | 9.24 (0.21, 705.16) | 6.47 (0.16, 449.24) | 14.97 (0.23, 1684.3) | 3 (0.06, 232.51) | 4.96 (0.13, 329.89) | 2.77 (0.07, 194.48) | 5.45 (0.15, 348.08) | 15.99 (0.2, 2178.17) | 2.77 (0.13, 118) | 1.83 (0.04, 141.18) | 1.99 (0.04, 156.91) | 3.25 (0.08, 224.92) | Miocamycin10d | |  |
| 1.81 (0.24, 15.21) | 0.17 (0.02, 1.43) | 1.07 (0.22, 5.17) | 0.55 (0.16, 1.81) | 0.89 (0.28, 2.86) | 0.89 (0.2, 3.94) | 0.54 (0.27, 1.14) | 0.39 (0.1, 1.6) | 0.85 (0.16, 4.51) | 2 (0.44, 10.18) | 0.53 (0.12, 2.36) | 0.77 (0.08, 7.48) | **3.99 (1.33, 12.2)** | 2.41 (0.82, 7.2) | 1.66 (0.23, 12.88) | 5.31 (0.76, 44.53) | 0.62 (0.11, 3.51) | 3.15 (0.65, 15.2) | 2.2 (0.62, 7.99) | 4.99 (0.5, 60.49) | 1.02 (0.19, 5.46) | 1.71 (0.6, 4.87) | 0.94 (0.29, 3.39) | 1.87 (0.72, 4.76) | 5.37 (0.38, 89.21) | 0.97 (0.18, 5.12) | 0.63 (0.15, 3) | 0.68 (0.13, 3.53) | 1.12 (0.33, 3.68) | 0.35 (0.01, 10.95) | PenicillinV10d | |
| 1.86 (0.2, 19.3) | 0.17 (0.01, 2.04) | 1.1 (0.14, 8.4) | 0.57 (0.1, 3.13) | 0.91 (0.15, 5.21) | 0.91 (0.12, 6.44) | 0.56 (0.14, 2.28) | 0.4 (0.07, 2.13) | 0.87 (0.1, 7.15) | 2.06 (0.31, 14.48) | 0.54 (0.07, 3.73) | 0.8 (0.06, 10.44) | 4.11 (0.72, 22.29) | 2.49 (0.45, 13.45) | 1.71 (0.16, 19.1) | 5.49 (0.52, 65.63) | 0.64 (0.07, 5.48) | 3.25 (0.41, 24.5) | 2.27 (0.35, 13.89) | 5.14 (0.42, 72.51) | 1.05 (0.12, 8.55) | 1.76 (0.32, 9.25) | 0.97 (0.27, 3.66) | 1.93 (0.37, 9.38) | 5.53 (0.32, 106.52) | 0.99 (0.12, 8.05) | 0.64 (0.11, 3.96) | 0.7 (0.08, 5.57) | 1.15 (0.19, 6.62) | 0.35 (0, 14.02) | 1.03 (0.27, 3.72) | Telithromycin5d |

Values are presented as odds ratios (95% confidence intervals). Penicillin V 10d is used as reference. Statistically significant results are highlighted in bold. Abbreviations: AMC, amoxicillin-clavulanate; BPV, Benzathine Penicillin V.

**Supplementary Table 6. League table of pairwise comparisons for late bacterial eradication in the pediatric subgroup**

| AMC |  |  |  |  |  |  |  |  |  |  |  |  |  |  |  |
| --- | --- | --- | --- | --- | --- | --- | --- | --- | --- | --- | --- | --- | --- | --- | --- |
| 1.27 (0.45, 3.62) | Amoxicillin |  |  |  |  |  |  |  |  |  |  |  |  |  |  |
| 3.21 (1.06, 9.01) | 2.54 (1.09, 5.36) | Azithromycin |  |  |  |  |  |  |  |  |  |  |  |  |  |
| 1.48 (0.36, 6.28) | 1.17 (0.43, 3.13) | 0.46 (0.14, 1.72) | BenzathinePenicillinG | |  |  |  |  |  |  |  |  |  |  |  |
| 1.44 (0.57, 3.65) | 1.14 (0.48, 2.63) | 0.45 (0.19, 1.13) | 0.98 (0.26, 3.56) | Cefaclor |  |  |  |  |  |  |  |  |  |  |  |
| 1.39 (0.27, 7.26) | 1.1 (0.25, 4.69) | 0.43 (0.1, 1.98) | 0.94 (0.16, 5.48) | 0.96 (0.2, 4.69) | Cefdinir |  |  |  |  |  |  |  |  |  |  |
| 0.94 (0.11, 7.79) | 0.74 (0.1, 5.35) | 0.29 (0.04, 2.23) | 0.64 (0.07, 5.81) | 0.65 (0.08, 5.15) | 0.68 (0.07, 6.77) | Cefixime |  |  |  |  |  |  |  |  |  |
| 0.89 (0.24, 3.35) | 0.71 (0.24, 2.05) | **0.28 (0.1, 0.89)** | 0.61 (0.14, 2.61) | 0.62 (0.18, 2.15) | 0.64 (0.13, 3.22) | 0.95 (0.12, 7.52) | CefpodoximeProxetil | |  |  |  |  |  |  |  |
| 0.21 (0.02, 1.43) | 0.17 (0.02, 1.04) | **0.07 (0.01, 0.43)** | 0.14 (0.01, 1.14) | **0.15 (0.01, 0.99)** | 0.15 (0.01, 1.36) | 0.22 (0.01, 2.99) | 0.24 (0.02, 1.69) | Cefprozil |  |  |  |  |  |  |  |
| 0.72 (0.19, 2.58) | 0.57 (0.18, 1.58) | **0.22 (0.08, 0.67)** | 0.49 (0.11, 2.01) | 0.5 (0.14, 1.64) | 0.52 (0.1, 2.46) | 0.77 (0.09, 5.88) | 0.81 (0.22, 2.76) | 3.39 (0.47, 36.32) | CefuroximeAxetil |  |  |  |  |  |  |
| 0.79 (0.16, 3.92) | 0.62 (0.15, 2.57) | 0.24 (0.06, 1.09) | 0.53 (0.09, 2.96) | 0.54 (0.11, 2.59) | 0.57 (0.09, 3.57) | 0.83 (0.08, 8.13) | 0.88 (0.18, 4.26) | 3.75 (0.42, 48.57) | 1.09 (0.23, 5.49) | Cephalexin |  |  |  |  |  |
| 2.4 (0.79, 7.08) | 1.89 (0.63, 5.37) | 0.74 (0.25, 2.28) | 1.63 (0.37, 6.79) | 1.66 (0.51, 5.17) | 1.73 (0.33, 8.47) | 2.53 (0.31, 20.18) | 2.68 (0.72, 9.55) | **11.21 (1.98, 104.65)** | 3.32 (0.93, 12.27) | 3.05 (0.61, 14.86) | Clarithromycin |  |  |  |  |
| 1.55 (0.27, 8.25) | 1.22 (0.19, 7.19) | 0.48 (0.07, 3.04) | 1.05 (0.13, 7.93) | 1.07 (0.19, 5.54) | 1.12 (0.11, 10.19) | 1.63 (0.12, 21.52) | 1.74 (0.22, 12.53) | 7.41 (0.6, 121.64) | 2.15 (0.28, 16.04) | 1.96 (0.21, 17.73) | 0.65 (0.09, 4.32) | Erythromycin |  |  |  |
| 1.24 (0.23, 6.89) | 0.98 (0.21, 4.53) | 0.39 (0.09, 1.9) | 0.84 (0.13, 5.16) | 0.86 (0.17, 4.5) | 0.89 (0.13, 6.2) | 1.31 (0.13, 13.7) | 1.39 (0.26, 7.39) | 5.94 (0.61, 82.85) | 1.72 (0.34, 9.54) | 1.57 (0.23, 10.79) | 0.52 (0.1, 2.86) | 0.8 (0.08, 8.17) | Loracarbef |  |  |
| 1.3 (0.5, 3.37) | 1.02 (0.56, 1.84) | **0.4 (0.22, 0.79)** | 0.88 (0.27, 2.75) | 0.9 (0.39, 2.1) | 0.94 (0.25, 3.51) | 1.38 (0.21, 8.89) | 1.45 (0.58, 3.57) | **6.08 (1.08, 57.27)** | 1.79 (0.76, 4.58) | 1.65 (0.45, 6.07) | 0.54 (0.22, 1.39) | 0.83 (0.14, 5.35) | 1.05 (0.25, 4.26) | PenicillinV |  |
| 0.91 (0.18, 4.71) | 0.72 (0.16, 3.07) | 0.28 (0.07, 1.3) | 0.62 (0.1, 3.54) | 0.63 (0.13, 3.05) | 0.66 (0.1, 4.27) | 0.97 (0.1, 9.48) | 1.02 (0.2, 5.1) | 4.37 (0.47, 56.59) | 1.26 (0.26, 6.52) | 1.16 (0.18, 7.45) | 0.38 (0.08, 1.96) | 0.59 (0.06, 5.7) | 0.74 (0.1, 5.07) | 0.7 (0.18, 2.67) | Spiramycin |

Values are presented as odds ratios (95% confidence intervals). Penicillin V is used as reference. Statistically significant results are highlighted in bold. Abbreviations: AMC, amoxicillin-clavulanate.

**Supplementary Table 7. League table of pairwise comparisons for late bacterial eradication in the adult subgroup**

| Amoxicillin |  |  |  |  |  |  |  |  |
| --- | --- | --- | --- | --- | --- | --- | --- | --- |
| 2.5 (0.35, 16.39) | Azithromycin |  |  |  |  |  |  |  |
| 0.87 (0.14, 5.43) | 0.34 (0.06, 2.2) | Cefotiam |  |  |  |  |  |  |
| 0.66 (0.08, 5.3) | 0.26 (0.03, 2.18) | 0.76 (0.1, 5.71) | CefpodoximeProxetil | |  |  |  |  |
| 1.14 (0.23, 5.32) | 0.45 (0.15, 1.36) | 1.3 (0.3, 5.47) | 1.73 (0.28, 10.34) | Clarithromycin |  |  |  |  |
| 0.63 (0.1, 3.72) | 0.25 (0.04, 1.55) | 0.73 (0.14, 3.88) | 0.97 (0.13, 6.85) | 0.56 (0.14, 2.31) | Dirithromycin |  |  |  |
| 0.42 (0.05, 3.78) | 0.17 (0.02, 1.56) | 0.49 (0.06, 4) | 0.65 (0.06, 6.83) | 0.37 (0.06, 2.57) | 0.67 (0.18, 2.46) | Erythromycin |  |  |
| 0.62 (0.1, 3.81) | 0.25 (0.04, 1.6) | 0.71 (0.13, 4.02) | 0.95 (0.13, 7.19) | 0.55 (0.13, 2.43) | 0.98 (0.19, 5.26) | 1.46 (0.18, 12.02) | Loracarbef |  |
| 0.78 (0.2, 3) | 0.31 (0.08, 1.24) | 0.9 (0.26, 3.06) | 1.19 (0.23, 6.03) | 0.69 (0.32, 1.57) | 1.23 (0.39, 3.89) | 1.83 (0.33, 10.36) | 1.25 (0.37, 4.19) | PenicillinV |

Values are presented as odds ratios (95% confidence intervals). Penicillin V is used as reference.

**Supplementary Table 8. League table of pairwise comparisons for late bacterial eradication stratified by treatment duration**

| AMC10d |  |  |  |  |  |  |  |  |  |  |  |  |  |  |  |  |  |  |  |  |  |  |  |  |  |  |  |
| --- | --- | --- | --- | --- | --- | --- | --- | --- | --- | --- | --- | --- | --- | --- | --- | --- | --- | --- | --- | --- | --- | --- | --- | --- | --- | --- | --- |
| 1.53 (0.34, 8.2) | AMC5d |  |  |  |  |  |  |  |  |  |  |  |  |  |  |  |  |  |  |  |  |  |  |  |  |  |  |
| 2.17 (0.5, 10.69) | 1.41 (0.53, 3.89) | Amoxicillin10d | |  |  |  |  |  |  |  |  |  |  |  |  |  |  |  |  |  |  |  |  |  |  |  |  |
| 3.7 (0.84, 19.32) | 2.41 (0.79, 7.59) | 1.72 (0.63, 4.55) | Amoxicillin6d | |  |  |  |  |  |  |  |  |  |  |  |  |  |  |  |  |  |  |  |  |  |  |  |
| 2.53 (0.57, 13.11) | 1.65 (0.55, 4.88) | 1.18 (0.44, 2.94) | 0.69 (0.23, 1.98) | Amoxicillin7d | |  |  |  |  |  |  |  |  |  |  |  |  |  |  |  |  |  |  |  |  |  |  |
| **5.86 (1.6, 23.78)** | **3.83 (1.45, 9.24)** | **2.7 (1.25, 5.46)** | 1.59 (0.61, 3.84) | 2.32 (0.92, 5.32) | Azithromycin3d | |  |  |  |  |  |  |  |  |  |  |  |  |  |  |  |  |  |  |  |  |  |
| **5.54 (1.03, 33.95)** | 3.61 (0.91, 14) | 2.56 (0.71, 8.75) | 1.49 (0.39, 5.68) | 2.2 (0.58, 8.23) | 0.94 (0.3, 3.25) | Azithromycin5d | |  |  |  |  |  |  |  |  |  |  |  |  |  |  |  |  |  |  |  |  |
| 2.49 (0.48, 14.43) | 1.63 (0.49, 5.69) | 1.15 (0.57, 2.35) | 0.67 (0.21, 2.34) | 0.98 (0.31, 3.33) | 0.43 (0.16, 1.25) | 0.45 (0.11, 1.96) | BenzathinePenicillinG1d | | |  |  |  |  |  |  |  |  |  |  |  |  |  |  |  |  |  |  |
| 1.1 (0.47, 2.67) | 0.72 (0.16, 2.84) | 0.51 (0.13, 1.81) | 0.3 (0.07, 1.15) | 0.44 (0.1, 1.64) | **0.19 (0.06, 0.57)** | **0.2 (0.04, 0.97)** | 0.44 (0.09, 1.87) | Cefaclor10d | |  |  |  |  |  |  |  |  |  |  |  |  |  |  |  |  |  |  |
| 3.87 (0.79, 22.22) | 2.49 (0.79, 8.66) | 1.77 (0.85, 3.87) | 1.03 (0.33, 3.53) | 1.51 (0.5, 5.09) | 0.66 (0.26, 1.83) | 0.7 (0.17, 3.01) | 1.54 (0.56, 4.38) | 3.5 (0.85, 16.26) | Cefaclor5d | |  |  |  |  |  |  |  |  |  |  |  |  |  |  |  |  |  |
| 1.88 (0.43, 9.3) | 1.22 (0.41, 3.47) | 0.87 (0.33, 2.1) | 0.51 (0.17, 1.44) | 0.74 (0.26, 2) | **0.32 (0.14, 0.76)** | 0.34 (0.09, 1.22) | 0.76 (0.22, 2.28) | 1.69 (0.45, 6.9) | 0.49 (0.15, 1.45) | Cefdinir10d | |  |  |  |  |  |  |  |  |  |  |  |  |  |  |  |  |
| 1.76 (0.22, 15.16) | 1.13 (0.19, 7.28) | 0.82 (0.15, 4.57) | 0.47 (0.08, 2.8) | 0.7 (0.12, 4.1) | 0.3 (0.06, 1.64) | 0.31 (0.05, 2.24) | 0.7 (0.11, 4.46) | 1.61 (0.22, 11.92) | 0.46 (0.07, 2.78) | 0.95 (0.17, 5.39) | Cefixime10d | |  |  |  |  |  |  |  |  |  |  |  |  |  |  |  |
| 2.65 (0.51, 16.11) | 1.73 (0.48, 6.3) | 1.23 (0.38, 3.9) | 0.71 (0.2, 2.6) | 1.05 (0.3, 3.7) | 0.45 (0.15, 1.43) | 0.48 (0.11, 2.18) | 1.07 (0.27, 4.07) | 2.42 (0.52, 12.1) | 0.69 (0.18, 2.57) | 1.41 (0.43, 5) | 1.49 (0.22, 9.87) | Cefotiam5d | |  |  |  |  |  |  |  |  |  |  |  |  |  |  |
| 2.61 (0.53, 14.37) | 1.7 (0.52, 5.61) | 1.21 (0.41, 3.39) | 0.7 (0.22, 2.27) | 1.03 (0.33, 3.25) | 0.44 (0.17, 1.26) | 0.47 (0.12, 1.93) | 1.05 (0.29, 3.6) | 2.37 (0.58, 10.71) | 0.68 (0.19, 2.27) | 1.39 (0.47, 4.36) | 1.47 (0.23, 9.01) | 0.99 (0.26, 3.71) | CefpodoximeProxetil10d | | |  |  |  |  |  |  |  |  |  |  |  |  |
| 1.22 (0.28, 6.24) | 0.8 (0.28, 2.34) | 0.56 (0.23, 1.44) | **0.33 (0.12, 0.95)** | 0.48 (0.18, 1.36) | **0.21 (0.09, 0.52)** | **0.22 (0.06, 0.83)** | 0.49 (0.15, 1.62) | 1.12 (0.3, 4.63) | **0.32 (0.1, 0.97)** | 0.65 (0.26, 1.81) | 0.7 (0.12, 3.99) | 0.46 (0.14, 1.59) | 0.47 (0.16, 1.44) | CefpodoximeProxetil5d | | |  |  |  |  |  |  |  |  |  |  |  |
| 2.57 (0.21, 75.29) | 1.74 (0.11, 60.91) | 1.2 (0.08, 40.21) | 0.7 (0.04, 24.36) | 1.02 (0.06, 36.86) | 0.44 (0.03, 14.9) | 0.47 (0.03, 17.88) | 1.06 (0.06, 36.17) | 2.29 (0.22, 63.81) | 0.68 (0.04, 24.02) | 1.41 (0.09, 49.53) | 1.49 (0.06, 64.32) | 0.98 (0.06, 38.91) | 1 (0.06, 34.53) | 2.12 (0.14, 78.53) | Cefprozil10d | |  |  |  |  |  |  |  |  |  |  |  |
| 0.29 (0.03, 2.4) | 0.19 (0.02, 1.06) | **0.13 (0.02, 0.73)** | **0.08 (0.01, 0.45)** | **0.11 (0.01, 0.63)** | **0.05 (0.01, 0.25)** | **0.05 (0.01, 0.31)** | **0.11 (0.01, 0.76)** | 0.26 (0.03, 1.79) | **0.08 (0.01, 0.47)** | **0.16 (0.02, 0.83)** | 0.16 (0.01, 1.54) | **0.11 (0.01, 0.71)** | **0.11 (0.01, 0.69)** | 0.24 (0.03, 1.24) | 0.11 (0, 2.46) | Cefprozil5d | |  |  |  |  |  |  |  |  |  |  |
| 0.77 (0.16, 4.4) | 0.5 (0.15, 1.71) | 0.36 (0.11, 1.05) | **0.21 (0.06, 0.69)** | **0.3 (0.09, 0.99)** | **0.13 (0.05, 0.39)** | **0.14 (0.03, 0.58)** | 0.31 (0.08, 1.12) | 0.7 (0.17, 3.29) | **0.2 (0.05, 0.7)** | 0.41 (0.13, 1.34) | 0.44 (0.06, 2.76) | 0.29 (0.07, 1.14) | 0.29 (0.08, 1.04) | 0.63 (0.19, 1.96) | 0.29 (0.01, 5.26) | 2.69 (0.44, 25.61) | CefuroximeAxetil10d | |  |  |  |  |  |  |  |  |  |
| 1.9 (0.45, 9.67) | 1.24 (0.42, 3.58) | 0.88 (0.34, 2.14) | 0.51 (0.18, 1.46) | 0.75 (0.27, 2.09) | **0.32 (0.15, 0.8)** | 0.34 (0.09, 1.27) | 0.77 (0.23, 2.34) | 1.72 (0.47, 7.19) | 0.5 (0.15, 1.47) | 1.02 (0.39, 2.81) | 1.08 (0.18, 6.08) | 0.72 (0.21, 2.43) | 0.73 (0.24, 2.2) | 1.57 (0.57, 4) | 0.74 (0.02, 11.22) | **6.54 (1.24, 57.78)** | 2.46 (0.78, 7.91) | CefuroximeAxetil5d | |  |  |  |  |  |  |  |  |
| 1.46 (0.31, 7.97) | 0.95 (0.29, 3.06) | 0.67 (0.23, 1.91) | 0.39 (0.12, 1.24) | 0.57 (0.18, 1.78) | **0.25 (0.09, 0.69)** | 0.26 (0.06, 1.05) | 0.58 (0.16, 2.12) | 1.33 (0.32, 5.94) | 0.38 (0.1, 1.26) | 0.78 (0.26, 2.37) | 0.83 (0.14, 4.91) | 0.55 (0.14, 2.03) | 0.56 (0.16, 1.86) | 1.2 (0.38, 3.48) | 0.55 (0.02, 9.32) | 5.02 (0.87, 47.09) | 1.88 (0.53, 6.79) | 0.76 (0.25, 2.35) | Cephalexin10d | |  |  |  |  |  |  |  |
| 2.51 (0.58, 12.36) | 1.63 (0.56, 4.67) | 1.16 (0.45, 2.84) | 0.68 (0.23, 1.9) | 0.99 (0.36, 2.72) | 0.43 (0.19, 1.02) | 0.45 (0.19, 1.06) | 1.01 (0.31, 3.11) | 2.31 (0.59, 9.22) | 0.66 (0.2, 1.97) | 1.35 (0.51, 3.64) | 1.43 (0.24, 7.89) | 0.95 (0.28, 3.18) | 0.97 (0.32, 2.89) | 2.06 (0.76, 5.31) | 0.96 (0.03, 15.64) | **8.73 (1.83, 69.14)** | **3.27 (1.03, 10.29)** | 1.32 (0.5, 3.49) | 1.72 (0.58, 5.26) | Clarithromycin10d | |  |  |  |  |  |  |
| 4.65 (1.15, 22.46) | 3.07 (1.36, 6.78) | 2.16 (0.95, 4.91) | 1.27 (0.47, 3.28) | 1.85 (0.74, 4.64) | 0.8 (0.4, 1.71) | 0.85 (0.25, 2.89) | 1.88 (0.62, 5.62) | **4.25 (1.23, 16.33)** | 1.22 (0.41, 3.36) | 2.52 (1.05, 6.28) | 2.68 (0.47, 14.24) | 1.77 (0.56, 5.55) | 1.8 (0.64, 4.99) | **3.85 (1.55, 8.97)** | 1.8 (0.05, 27.23) | **16.14 (3.17, 137.13)** | **6.11 (2.08, 18.4)** | **2.47 (1.01, 5.95)** | **3.23 (1.17, 8.97)** | 1.87 (0.77, 4.56) | Clarithromycin5d | |  |  |  |  |  |
| 2.47 (0.18, 64.12) | 1.64 (0.07, 54.63) | 1.16 (0.05, 37) | 0.67 (0.03, 23.02) | 0.99 (0.04, 33.9) | 0.43 (0.02, 13.61) | 0.46 (0.02, 17.26) | 1 (0.04, 33.84) | 2.25 (0.14, 65.42) | 0.64 (0.03, 21.68) | 1.35 (0.06, 44.73) | 1.41 (0.05, 64.51) | 0.96 (0.04, 33.36) | 0.97 (0.04, 33.65) | 2.07 (0.09, 68.03) | 0.91 (0.01, 58.94) | 9.23 (0.28, 454.52) | 3.28 (0.14, 116.8) | 1.32 (0.06, 43.94) | 1.71 (0.07, 60.21) | 1.01 (0.05, 34.05) | 0.53 (0.02, 17.29) | Clindamycin10d | |  |  |  |  |
| 1.93 (0.5, 8.16) | 1.26 (0.38, 4.02) | 0.89 (0.31, 2.42) | 0.52 (0.16, 1.62) | 0.76 (0.25, 2.3) | **0.33 (0.13, 0.86)** | 0.35 (0.09, 1.36) | 0.78 (0.21, 2.58) | 1.77 (0.52, 6.17) | 0.5 (0.14, 1.62) | 1.03 (0.35, 3.09) | 1.08 (0.18, 6.52) | 0.73 (0.19, 2.64) | 0.74 (0.22, 2.42) | 1.58 (0.52, 4.49) | 0.74 (0.02, 10.92) | **6.67 (1.14, 66.61)** | 2.49 (0.7, 8.88) | 1.02 (0.34, 2.96) | 1.32 (0.39, 4.43) | 0.77 (0.26, 2.3) | 0.41 (0.15, 1.11) | 0.77 (0.02, 15.66) | Dirithromycin10d | |  |  |  |
| 1.33 (0.37, 4.95) | 0.86 (0.2, 3.49) | 0.61 (0.16, 2.16) | 0.36 (0.08, 1.39) | 0.53 (0.13, 2.01) | **0.23 (0.07, 0.74)** | 0.24 (0.05, 1.16) | 0.53 (0.11, 2.23) | 1.21 (0.37, 3.85) | 0.35 (0.07, 1.4) | 0.71 (0.18, 2.69) | 0.75 (0.1, 5.38) | 0.5 (0.1, 2.23) | 0.5 (0.11, 2.14) | 1.08 (0.27, 3.93) | 0.51 (0.02, 7.32) | 4.64 (0.63, 51.23) | 1.73 (0.38, 7.51) | 0.7 (0.17, 2.58) | 0.9 (0.21, 3.83) | 0.52 (0.13, 2.01) | **0.28 (0.08, 0.99)** | 0.53 (0.02, 10.39) | 0.69 (0.25, 1.79) | Erythromycin10d | |  |  |
| 2.14 (0.51, 10.53) | 1.39 (0.51, 3.84) | 0.99 (0.41, 2.32) | 0.58 (0.21, 1.58) | 0.85 (0.32, 2.22) | **0.36 (0.17, 0.83)** | 0.39 (0.11, 1.38) | 0.86 (0.27, 2.58) | 1.96 (0.55, 7.9) | 0.56 (0.18, 1.61) | 1.15 (0.46, 2.97) | 1.2 (0.22, 6.79) | 0.81 (0.25, 2.63) | 0.82 (0.29, 2.4) | 1.76 (0.69, 4.34) | 0.82 (0.02, 12.97) | **7.39 (1.42, 63.29)** | 2.77 (0.93, 8.66) | 1.12 (0.45, 2.92) | 1.47 (0.51, 4.31) | 0.85 (0.34, 2.19) | 0.46 (0.2, 1.05) | 0.86 (0.03, 18.48) | 1.11 (0.4, 3.2) | 1.61 (0.45, 6.29) | Loracarbef10d | |  |
| 2.37 (0.65, 10.18) | 1.55 (0.69, 3.47) | 1.1 (0.59, 1.96) | 0.65 (0.29, 1.4) | 0.94 (0.45, 1.97) | **0.41 (0.26, 0.68)** | 0.43 (0.14, 1.29) | 0.96 (0.37, 2.34) | 2.15 (0.69, 7.36) | 0.62 (0.24, 1.46) | 1.27 (0.64, 2.62) | 1.35 (0.26, 6.56) | 0.9 (0.33, 2.42) | 0.91 (0.38, 2.15) | 1.95 (0.96, 3.76) | 0.92 (0.03, 13.25) | **8.07 (1.78, 64.89)** | **3.07 (1.22, 7.98)** | 1.25 (0.62, 2.52) | 1.64 (0.7, 3.93) | 0.95 (0.47, 1.89) | **0.51 (0.29, 0.88)** | 0.95 (0.03, 19.23) | 1.23 (0.54, 2.89) | 1.79 (0.58, 6) | 1.11 (0.59, 2.06) | PenicillinV10d | |
| 2.42 (0.55, 12.41) | 1.59 (0.52, 4.76) | 1.12 (0.42, 2.89) | 0.66 (0.22, 1.94) | 0.96 (0.34, 2.76) | 0.41 (0.17, 1.05) | 0.44 (0.14, 1.34) | 0.97 (0.28, 3.22) | 2.22 (0.57, 9.23) | 0.63 (0.19, 1.98) | 1.31 (0.47, 3.73) | 1.39 (0.23, 7.97) | 0.92 (0.26, 3.19) | 0.93 (0.29, 2.94) | 1.98 (0.7, 5.37) | 0.94 (0.03, 15.34) | **8.32 (1.64, 73.71)** | 3.17 (0.96, 10.4) | 1.28 (0.46, 3.55) | 1.66 (0.54, 5.28) | 0.97 (0.47, 1.99) | 0.52 (0.2, 1.33) | 0.97 (0.03, 21) | 1.27 (0.41, 3.91) | 1.84 (0.48, 7.51) | 1.14 (0.42, 3) | 1.02 (0.48, 2.18) | Telithromycin5d |

Values are presented as odds ratios (95% confidence intervals). Penicillin V 10d is used as reference. Abbreviations: AMC, amoxicillin-clavulanate.

**Supplementary Table 9. League table of pairwise comparisons for early clinical response in the pediatric subgroup**

| AMC |  |  |  |  |  |  |  |  |  |  |  |  |  |  |  |  |
| --- | --- | --- | --- | --- | --- | --- | --- | --- | --- | --- | --- | --- | --- | --- | --- | --- |
| 2.62 (0.75, 9.82) | Amoxicillin |  |  |  |  |  |  |  |  |  |  |  |  |  |  |  |
| 1.58 (0.43, 6.16) | 0.6 (0.26, 1.33) | Azithromycin |  |  |  |  |  |  |  |  |  |  |  |  |  |  |
| 0.74 (0.04, 12.33) | 0.28 (0.02, 3.91) | 0.46 (0.03, 6.23) | BPG |  |  |  |  |  |  |  |  |  |  |  |  |  |
| 4.87 (0.34, 196.93) | 1.81 (0.15, 64.19) | 3.01 (0.27, 105.63) | 7.02 (0.19, 484.46) | BPV |  |  |  |  |  |  |  |  |  |  |  |  |
| 1.87 (0.45, 8.14) | 0.71 (0.28, 1.78) | 1.18 (0.43, 3.2) | 2.55 (0.17, 41.32) | 0.39 (0.01, 5.1) | Cefaclor |  |  |  |  |  |  |  |  |  |  |  |
| 0 (0, 704014165974.02) | 0 (0, 252432139953.64) | 0 (0, 417045306925.28) | 0 (0, 951901314372.46) | 0 (0, 141213076521.77) | 0 (0, 354082151428.34) | CefcapenePivoxil |  |  |  |  |  |  |  |  |  |  |
| 0.78 (0.17, 3.46) | **0.3 (0.09, 0.86)** | 0.49 (0.17, 1.34) | 1.06 (0.07, 15.53) | 0.16 (0, 1.95) | 0.42 (0.11, 1.53) | 2801.83 (0, 15487696981321533440) | Cefdinir |  |  |  |  |  |  |  |  |  |
| 0.62 (0.11, 3.79) | 0.24 (0.05, 1) | 0.39 (0.09, 1.58) | 0.84 (0.05, 15.64) | 0.13 (0, 1.95) | 0.33 (0.07, 1.7) | 2441.78 (0, 13103897953892866048) | 0.8 (0.18, 3.82) | Cefixime |  |  |  |  |  |  |  |  |
| 0.93 (0.2, 4.32) | 0.35 (0.11, 1.08) | 0.59 (0.2, 1.69) | 1.27 (0.09, 19.18) | 0.19 (0.01, 2.38) | 0.5 (0.13, 1.91) | 3359.92 (0, 18376482298833190912) | 1.19 (0.35, 4.19) | 1.51 (0.31, 7.05) | CefpodoximeProxetil | |  |  |  |  |  |  |
| 1.24 (0.09, 15.88) | 0.47 (0.04, 4.67) | 0.78 (0.07, 7.3) | 1.66 (0.05, 51.67) | 0.24 (0, 6.69) | 0.66 (0.05, 7.39) | 4600.94 (0, 28176989209606938624) | 1.59 (0.13, 17.09) | 1.99 (0.13, 25.51) | 1.34 (0.11, 14.51) | Cefprozil |  |  |  |  |  |  |
| 0.77 (0.19, 3.33) | **0.29 (0.11, 0.81)** | 0.49 (0.2, 1.27) | 1.06 (0.09, 12.52) | 0.16 (0, 1.92) | 0.41 (0.12, 1.46) | 2781.54 (0, 15675869953243158528) | 0.98 (0.34, 3.22) | 1.24 (0.29, 5.48) | 0.83 (0.27, 2.77) | 0.62 (0.06, 7.46) | CefuroximeAxetil |  |  |  |  |  |
| 1.47 (0.41, 5.87) | 0.56 (0.18, 1.77) | 0.93 (0.33, 2.78) | 2.04 (0.14, 31.46) | 0.31 (0.01, 4.02) | 0.79 (0.21, 3.11) | 5343.48 (0, 29922655131395403776) | 1.89 (0.56, 7.21) | 2.38 (0.5, 12) | 1.59 (0.45, 6.04) | 1.19 (0.11, 15.24) | 1.92 (0.59, 6.31) | Clarithromycin |  |  |  |  |
| 1.1 (0.12, 9.4) | 0.42 (0.06, 2.65) | 0.7 (0.1, 4) | 1.5 (0.06, 33.82) | 0.22 (0, 4.33) | 0.59 (0.07, 4.25) | 4027.25 (0, 23823640258774282240) | 1.43 (0.18, 10.03) | 1.79 (0.18, 15.48) | 1.2 (0.15, 8.27) | 0.89 (0.22, 3.71) | 1.43 (0.19, 9.24) | 0.75 (0.09, 5.21) | Erythromycin |  |  |  |
| 0.73 (0.07, 6.27) | 0.28 (0.04, 1.81) | 0.46 (0.06, 2.93) | 0.99 (0.04, 21.25) | 0.15 (0, 2.92) | 0.39 (0.04, 2.96) | 2788.34 (0, 15265803827252310016) | 0.95 (0.11, 6.67) | 1.18 (0.11, 10.09) | 0.79 (0.09, 5.71) | 0.59 (0.03, 10.9) | 0.95 (0.12, 6.16) | 0.49 (0.06, 3.51) | 0.67 (0.05, 8.5) | Loracarbef |  |  |
| 1.56 (0.46, 5.5) | 0.6 (0.29, 1.17) | 0.99 (0.54, 1.76) | 2.14 (0.17, 27.51) | 0.33 (0.01, 3.4) | 0.84 (0.3, 2.3) | 5645.05 (0, 29452097120995635200) | 2.01 (0.87, 4.8) | 2.51 (0.7, 9.21) | 1.69 (0.69, 4.13) | 1.26 (0.14, 13.18) | 2.04 (0.94, 4) | 1.06 (0.4, 2.63) | 1.41 (0.25, 9.27) | 2.14 (0.37, 14.98) | PenicillinV |  |
| 3.89 (0.35, 49.26) | 1.47 (0.17, 15.39) | 2.42 (0.29, 26.04) | 5.4 (0.2, 153.69) | 0.78 (0.01, 21.66) | 2.09 (0.21, 24.63) | 14593.08 (0, 85446586893183303680) | 4.93 (0.55, 56.13) | 6.18 (0.54, 91.57) | 4.12 (0.45, 47.82) | 3.19 (0.15, 77.7) | 4.97 (0.56, 54.07) | 2.6 (0.27, 30.16) | 3.54 (0.24, 62.64) | 5.38 (0.35, 101.52) | 2.43 (0.32, 24.49) | Spiramycin |

Values are presented as odds ratios (95% confidence intervals). Penicillin V is used as reference. Statistically significant results are highlighted in bold. Abbreviations: AMC, amoxicillin-clavulanate; BPG, Benzathine Penicillin G; BPV, Benzathine Penicillin V.

**Supplementary Table 10. League table of pairwise comparisons for early clinical response in the adult subgroup**

| Amoxicillin |  |  |  |  |  |  |  |  |
| --- | --- | --- | --- | --- | --- | --- | --- | --- |
| 0.7 (0.01, 53.42) | Azithromycin |  |  |  |  |  |  |  |
| 1.03 (0.05, 20.31) | 1.46 (0.02, 137.12) | CefpodoximeProxetil | |  |  |  |  |  |
| 0.63 (0.07, 5.68) | 0.89 (0.02, 52.12) | 0.61 (0.05, 7.38) | Clarithromycin |  |  |  |  |  |
| 0.86 (0.07, 10.3) | 1.21 (0.02, 80.97) | 0.84 (0.05, 12.94) | 1.37 (0.25, 7.39) | Dirithromycin |  |  |  |  |
| 0.82 (0.06, 10.71) | 1.16 (0.02, 79.94) | 0.8 (0.05, 13.35) | 1.3 (0.27, 6.54) | 0.95 (0.21, 4.56) | Erythromycin |  |  |  |
| 0.71 (0.04, 11.6) | 1 (0.01, 82.08) | 0.68 (0.03, 14.65) | 1.13 (0.1, 11.3) | 0.82 (0.06, 10.62) | 0.86 (0.05, 11.92) | Loracarbef |  |  |
| 0 (0, 6254947.23) | 0 (0, 10318070.93) | 0 (0, 5843912.12) | 0 (0, 9738767.54) | 0 (0, 6355772.49) | 0 (0, 6935120.19) | 0 (0, 9966878.74) | Miocamycin |  |
| 1.15 (0.16, 7.97) | 1.64 (0.03, 86.35) | 1.12 (0.12, 10.62) | 1.83 (0.61, 5.54) | 1.33 (0.28, 6.42) | 1.42 (0.25, 7.52) | 1.63 (0.21, 14.03) | 1416.03 (0, 346907562058458816) | PenicillinV |

Values are presented as odds ratios (95% confidence intervals). Penicillin V is used as reference.

**Supplementary Table 11. League table of pairwise comparisons for early clinical response stratified by treatment duration**

| AMC10d |  |  |  |  |  |  |  |  |  |  |  |  |  |  |  |  |  |  |  |  |  |  |  |  |  |  |  |  |  |  |
| --- | --- | --- | --- | --- | --- | --- | --- | --- | --- | --- | --- | --- | --- | --- | --- | --- | --- | --- | --- | --- | --- | --- | --- | --- | --- | --- | --- | --- | --- | --- |
| 0.59 (0.01, 29.81) | AMC3d |  |  |  |  |  |  |  |  |  |  |  |  |  |  |  |  |  |  |  |  |  |  |  |  |  |  |  |  |  |
| 0.6 (0.03, 12.32) | 0.93 (0.04, 45.01) | AMC5d |  |  |  |  |  |  |  |  |  |  |  |  |  |  |  |  |  |  |  |  |  |  |  |  |  |  |  |  |
| 3.44 (0.15, 78.11) | 5.56 (0.52, 158.63) | 5.6 (0.79, 51.65) | Amoxicillin10d | |  |  |  |  |  |  |  |  |  |  |  |  |  |  |  |  |  |  |  |  |  |  |  |  |  |  |
| 1.09 (0.06, 17.97) | 1.76 (0.09, 69.75) | 1.82 (0.4, 8.8) | 0.32 (0.05, 1.64) | Amoxicillin6d | |  |  |  |  |  |  |  |  |  |  |  |  |  |  |  |  |  |  |  |  |  |  |  |  |  |
| 1.9 (0.1, 31.73) | 3.13 (0.15, 126.96) | 3.14 (0.69, 16.37) | 0.56 (0.08, 3.13) | 1.75 (0.59, 5.4) | Amoxicillin7d | |  |  |  |  |  |  |  |  |  |  |  |  |  |  |  |  |  |  |  |  |  |  |  |  |
| 1.08 (0.07, 16.13) | 1.8 (0.1, 63.67) | 1.8 (0.45, 8.43) | 0.32 (0.06, 1.25) | 1 (0.41, 2.48) | 0.57 (0.21, 1.51) | Azithromycin3d | |  |  |  |  |  |  |  |  |  |  |  |  |  |  |  |  |  |  |  |  |  |  |  |
| 0.61 (0.01, 70.44) | 1.02 (0.01, 172.99) | 1.01 (0.02, 56.25) | 0.17 (0, 11.65) | 0.56 (0.02, 27.24) | 0.32 (0.01, 15.99) | 0.56 (0.02, 26.34) | Azithromycin5d | |  |  |  |  |  |  |  |  |  |  |  |  |  |  |  |  |  |  |  |  |  |  |
| 3.02 (0.1, 254.43) | 5.19 (0.12, 804.5) | 4.98 (0.37, 210.06) | 0.88 (0.05, 41.47) | 2.7 (0.29, 105.29) | 1.56 (0.16, 59.87) | 2.64 (0.3, 100.78) | 4.98 (0.07, 708.42) | BPV10d |  |  |  |  |  |  |  |  |  |  |  |  |  |  |  |  |  |  |  |  |  |  |
| 1 (0.09, 9.66) | 1.62 (0.07, 67.96) | 1.64 (0.22, 12.69) | 0.28 (0.03, 2.11) | 0.89 (0.17, 4.68) | 0.51 (0.1, 2.74) | 0.89 (0.21, 3.73) | 1.6 (0.02, 72.22) | 0.33 (0.01, 4.5) | Cefaclor10d | |  |  |  |  |  |  |  |  |  |  |  |  |  |  |  |  |  |  |  |  |
| 2.43 (0.1, 52.83) | 3.97 (0.32, 120.27) | 3.95 (0.55, 36.71) | 0.7 (0.28, 1.7) | 2.19 (0.41, 14.5) | 1.25 (0.22, 8.53) | 2.15 (0.53, 11.45) | 4.07 (0.06, 182.68) | 0.8 (0.02, 13.78) | 2.46 (0.32, 21.18) | Cefaclor5d | |  |  |  |  |  |  |  |  |  |  |  |  |  |  |  |  |  |  |  |
| 2.24 (0.07, 74.78) | 3.7 (0.1, 234.42) | 3.7 (0.31, 55.97) | 0.64 (0.05, 10.73) | 2.02 (0.22, 24.3) | 1.14 (0.12, 13.81) | 2 (0.26, 20.33) | 3.77 (0.04, 249.31) | 0.72 (0.01, 17.4) | 2.22 (0.19, 35.01) | 0.91 (0.07, 15.32) | CefcapenePivoxil5d | |  |  |  |  |  |  |  |  |  |  |  |  |  |  |  |  |  |  |
| 0.28 (0.02, 4.48) | 0.46 (0.02, 18.24) | 0.46 (0.11, 2.27) | **0.08 (0.01, 0.44)** | **0.26 (0.1, 0.72)** | **0.15 (0.05, 0.43)** | **0.26 (0.11, 0.62)** | 0.47 (0.01, 16.46) | **0.1 (0, 0.89)** | 0.29 (0.06, 1.47) | **0.12 (0.02, 0.63)** | 0.13 (0.01, 1.17) | Cefdinir10d | |  |  |  |  |  |  |  |  |  |  |  |  |  |  |  |  |  |
| 0.79 (0.04, 12.56) | 1.3 (0.07, 49.49) | 1.3 (0.31, 6.22) | 0.24 (0.04, 1.15) | 0.73 (0.29, 1.89) | 0.42 (0.15, 1.14) | 0.73 (0.33, 1.59) | 1.31 (0.03, 45.92) | 0.27 (0.01, 2.44) | 0.82 (0.17, 4.04) | 0.34 (0.06, 1.69) | 0.36 (0.03, 3.18) | **2.81 (1.14, 7.1)** | Cefdinir5d | |  |  |  |  |  |  |  |  |  |  |  |  |  |  |  |  |
| 0.18 (0.01, 3.96) | 0.28 (0.01, 13.79) | 0.3 (0.04, 2.05) | **0.05 (0, 0.38)** | **0.16 (0.03, 0.79)** | **0.09 (0.01, 0.47)** | **0.16 (0.03, 0.72)** | 0.28 (0, 13.32) | **0.06 (0, 0.78)** | 0.18 (0.02, 1.42) | **0.08 (0.01, 0.56)** | 0.08 (0, 1) | 0.63 (0.1, 3.07) | 0.23 (0.04, 1.01) | Cefixime10d | |  |  |  |  |  |  |  |  |  |  |  |  |  |  |  |
| 1.76 (0.05, 61.07) | 3.06 (0.08, 177.23) | 2.96 (0.26, 41.53) | 0.52 (0.03, 7.24) | 1.63 (0.19, 17.93) | 0.92 (0.1, 10.35) | 1.6 (0.19, 16.15) | 2.96 (0.03, 168.41) | 0.57 (0.01, 14.84) | 1.82 (0.15, 26.07) | 0.73 (0.05, 10.99) | 0.79 (0.04, 18.92) | 6.23 (0.72, 68.19) | 2.22 (0.26, 22.34) | 10.23 (0.82, 166.4) | Cefixime5d | |  |  |  |  |  |  |  |  |  |  |  |  |  |  |
| 0.57 (0.03, 9.75) | 0.92 (0.04, 37.92) | 0.93 (0.19, 4.95) | **0.17 (0.02, 0.92)** | 0.52 (0.17, 1.64) | **0.3 (0.09, 0.99)** | 0.52 (0.18, 1.44) | 0.92 (0.02, 35.06) | 0.19 (0, 1.92) | 0.58 (0.1, 3.22) | 0.24 (0.03, 1.36) | 0.26 (0.02, 2.43) | 2 (0.65, 6.26) | 0.71 (0.25, 2.06) | 3.17 (0.6, 21.36) | 0.32 (0.03, 2.9) | CefpodoximeProxetil10d | | |  |  |  |  |  |  |  |  |  |  |  |  |
| 0.73 (0.04, 13.18) | 1.22 (0.06, 47.96) | 1.21 (0.24, 6.83) | 0.22 (0.03, 1.22) | 0.68 (0.21, 2.23) | 0.39 (0.11, 1.32) | 0.67 (0.23, 1.91) | 1.19 (0.02, 47.75) | 0.25 (0.01, 2.58) | 0.75 (0.13, 4.27) | 0.31 (0.04, 1.82) | 0.33 (0.03, 3.33) | 2.6 (0.82, 8.37) | 0.92 (0.3, 2.78) | 4.13 (0.78, 28.16) | 0.42 (0.04, 3.88) | 1.31 (0.36, 4.67) | CefpodoximeProxetil5d | | |  |  |  |  |  |  |  |  |  |  |  |
| 0.9 (0.06, 14.47) | 1.51 (0.06, 66.17) | 1.52 (0.22, 11.48) | 0.27 (0.03, 1.89) | 0.83 (0.18, 4.24) | 0.48 (0.09, 2.49) | 0.83 (0.21, 3.48) | 1.49 (0.02, 64.53) | 0.3 (0.01, 4.04) | 0.92 (0.24, 3.86) | 0.38 (0.05, 2.85) | 0.42 (0.03, 4.79) | 3.22 (0.69, 16.1) | 1.15 (0.25, 5.41) | 5.22 (0.71, 46.36) | 0.52 (0.04, 6.09) | 1.62 (0.31, 8.62) | 1.23 (0.24, 6.88) | Cefprozil10d | |  |  |  |  |  |  |  |  |  |  |  |
| 1.39 (0.06, 31) | 2.36 (0.09, 106.44) | 2.29 (0.31, 19.92) | 0.4 (0.04, 3.52) | 1.27 (0.24, 7.28) | 0.71 (0.13, 4.22) | 1.26 (0.26, 6.47) | 2.24 (0.03, 103.03) | 0.46 (0.01, 6.7) | 1.42 (0.18, 11.48) | 0.57 (0.06, 5.11) | 0.63 (0.04, 8.17) | 4.84 (0.93, 27.42) | 1.72 (0.34, 9.38) | 7.82 (0.98, 74.43) | 0.79 (0.05, 9.49) | 2.44 (0.42, 14.43) | 1.87 (0.32, 11.9) | 1.53 (0.2, 11.53) | CefuroximeAxetil4d | |  |  |  |  |  |  |  |  |  |  |
| 0.42 (0.02, 6.61) | 0.68 (0.03, 26.46) | 0.68 (0.17, 3.2) | **0.12 (0.02, 0.61)** | **0.39 (0.15, 0.97)** | **0.22 (0.08, 0.59)** | **0.38 (0.18, 0.82)** | 0.69 (0.01, 24.09) | 0.14 (0, 1.27) | 0.43 (0.09, 2.06) | **0.18 (0.03, 0.9)** | 0.19 (0.02, 1.68) | 1.48 (0.6, 3.67) | 0.53 (0.23, 1.19) | 2.35 (0.52, 14.38) | 0.24 (0.02, 1.97) | 0.74 (0.26, 2.11) | 0.57 (0.19, 1.69) | 0.46 (0.1, 2.02) | 0.3 (0.06, 1.52) | CefuroximeAxetil5d | |  |  |  |  |  |  |  |  |  |
| 0.69 (0.03, 12.14) | 1.16 (0.05, 46) | 1.14 (0.19, 7.28) | 0.2 (0.03, 1.24) | 0.63 (0.16, 2.48) | 0.36 (0.09, 1.49) | 0.64 (0.19, 2.05) | 1.1 (0.02, 45.2) | 0.23 (0.01, 2.52) | 0.71 (0.13, 3.81) | 0.29 (0.04, 1.8) | 0.31 (0.02, 3.21) | 2.45 (0.63, 9.47) | 0.87 (0.23, 3.12) | 3.86 (0.65, 28.71) | 0.39 (0.03, 4.13) | 1.23 (0.28, 5.16) | 0.94 (0.22, 4.03) | 0.75 (0.17, 3.34) | 0.5 (0.07, 3.35) | 1.65 (0.45, 5.88) | Clarithromycin10d | |  |  |  |  |  |  |  |  |
| 0.8 (0.05, 13.17) | 1.29 (0.07, 50.14) | 1.31 (0.37, 5.65) | 0.24 (0.04, 1.19) | 0.73 (0.29, 1.99) | 0.42 (0.15, 1.2) | 0.74 (0.33, 1.66) | 1.32 (0.03, 46.07) | 0.27 (0.01, 2.54) | 0.82 (0.17, 4.11) | 0.34 (0.05, 1.73) | 0.36 (0.03, 3.19) | **2.82 (1.14, 7.39)** | 1.01 (0.43, 2.43) | 4.48 (0.97, 28.14) | 0.46 (0.04, 3.9) | 1.42 (0.49, 4.26) | 1.09 (0.36, 3.37) | 0.88 (0.19, 4.1) | 0.59 (0.11, 2.91) | 1.91 (0.83, 4.58) | 1.16 (0.32, 4.45) | Clarithromycin5d | |  |  |  |  |  |  |  |
| 0.45 (0.2, 1) | 0.76 (0.01, 57.05) | 0.75 (0.03, 20.56) | 0.13 (0.01, 3.25) | 0.41 (0.02, 8.12) | 0.24 (0.01, 4.65) | 0.41 (0.02, 7.47) | 0.75 (0.01, 74.7) | 0.15 (0, 5.19) | 0.45 (0.04, 5.99) | 0.18 (0.01, 4.9) | 0.2 (0.01, 7.44) | 1.59 (0.09, 31.14) | 0.56 (0.03, 10.88) | 2.51 (0.1, 77.3) | 0.26 (0.01, 9.22) | 0.79 (0.04, 17.24) | 0.62 (0.03, 13.43) | 0.5 (0.03, 8.85) | 0.33 (0.01, 8.25) | 1.06 (0.06, 20.92) | 0.65 (0.03, 14.65) | 0.56 (0.03, 10.87) | Clindamycin10d | |  |  |  |  |  |  |
| 0.8 (0.05, 13.5) | 1.32 (0.06, 53.29) | 1.34 (0.24, 7.97) | 0.24 (0.03, 1.37) | 0.74 (0.19, 2.71) | 0.42 (0.11, 1.6) | 0.73 (0.23, 2.3) | 1.31 (0.03, 50.35) | 0.27 (0.01, 3.1) | 0.82 (0.16, 4.29) | 0.34 (0.05, 2.05) | 0.36 (0.03, 3.85) | 2.84 (0.78, 10.29) | 1.01 (0.29, 3.42) | 4.52 (0.75, 33.34) | 0.45 (0.04, 4.61) | 1.43 (0.35, 5.64) | 1.09 (0.26, 4.57) | 0.87 (0.2, 3.73) | 0.59 (0.09, 3.6) | 1.94 (0.55, 6.31) | 1.15 (0.31, 4.51) | 1.01 (0.28, 3.39) | 1.78 (0.09, 33.34) | Dirithromycin10d | |  |  |  |  |  |
| 0.77 (0.05, 12.59) | 1.31 (0.06, 51.05) | 1.3 (0.23, 7.74) | 0.23 (0.03, 1.32) | 0.72 (0.19, 2.7) | 0.41 (0.1, 1.6) | 0.71 (0.24, 2.18) | 1.28 (0.02, 50.41) | 0.26 (0.01, 2.86) | 0.79 (0.19, 3.58) | 0.33 (0.05, 1.97) | 0.35 (0.03, 3.51) | 2.78 (0.76, 10.32) | 0.98 (0.28, 3.41) | 4.41 (0.75, 31.7) | 0.45 (0.04, 4.38) | 1.4 (0.33, 5.63) | 1.06 (0.26, 4.48) | 0.86 (0.28, 2.47) | 0.56 (0.09, 3.65) | 1.87 (0.55, 6.39) | 1.13 (0.38, 3.48) | 0.98 (0.27, 3.35) | 1.72 (0.1, 31.68) | 0.98 (0.33, 2.82) | Erythromycin10d | |  |  |  |  |
| 1.04 (0.03, 34.33) | 1.73 (0.04, 115.91) | 1.73 (0.13, 24.06) | 0.3 (0.02, 4.25) | 0.96 (0.09, 9.86) | 0.55 (0.05, 5.8) | 0.94 (0.1, 9.25) | 1.71 (0.02, 111.23) | 0.34 (0.01, 7.73) | 1.05 (0.08, 15.51) | 0.43 (0.03, 6.25) | 0.47 (0.02, 10.38) | 3.68 (0.37, 38.49) | 1.32 (0.13, 12.92) | 5.92 (0.43, 97.67) | 0.58 (0.03, 11.71) | 1.86 (0.18, 20.36) | 1.42 (0.13, 15.59) | 1.13 (0.08, 15.95) | 0.76 (0.05, 11.47) | 2.48 (0.26, 24.86) | 1.52 (0.12, 18.88) | 1.31 (0.13, 12.76) | 2.32 (0.06, 84.29) | 1.32 (0.1, 15.14) | 1.32 (0.11, 15.98) | Erythromycin5d | |  |  |  |
| 0.55 (0.03, 9.27) | 0.89 (0.04, 36.31) | 0.91 (0.19, 4.91) | **0.16 (0.02, 0.91)** | 0.51 (0.16, 1.62) | **0.29 (0.08, 0.97)** | 0.51 (0.18, 1.41) | 0.91 (0.02, 33.15) | 0.19 (0, 1.86) | 0.56 (0.1, 3.36) | 0.23 (0.03, 1.38) | 0.25 (0.02, 2.59) | 1.96 (0.62, 6.05) | 0.7 (0.23, 2.02) | 3.15 (0.57, 21.18) | 0.31 (0.03, 2.98) | 0.98 (0.27, 3.47) | 0.75 (0.21, 2.76) | 0.6 (0.11, 3.24) | 0.4 (0.07, 2.32) | 1.33 (0.44, 3.83) | 0.81 (0.18, 3.54) | 0.7 (0.22, 2.01) | 1.23 (0.06, 23.4) | 0.69 (0.17, 2.89) | 0.7 (0.17, 3.03) | 0.53 (0.05, 5.59) | Loracarbef10d | |  |  |
| 0 (0, 248017053.07) | 0 (0, 418228654.14) | 0 (0, 289616554.25) | 0 (0, 59378338.94) | 0 (0, 169931536.35) | 0 (0, 95638973.73) | 0 (0, 166149611.96) | 0 (0, 236688565.95) | 0 (0, 45712168.38) | 0 (0, 201531683.67) | 0 (0, 86124107.46) | 0 (0, 48975612.25) | 0 (0, 623434860) | 0 (0, 226352889.95) | 0 (0, 1048357117.53) | 0 (0, 102794952.58) | 0 (0, 317142787.04) | 0 (0, 259085834.9) | 0 (0, 162686437.21) | 0 (0, 126263127.79) | 0 (0, 430251961.33) | 0 (0, 295177395.81) | 0 (0, 226840359.53) | 0 (0, 566389876.93) | 0 (0, 207028535.93) | 0 (0, 218865215.37) | 0 (0, 151241788.79) | 0 (0, 321126715.54) | Miocamycin10d | |  |
| 1.03 (0.06, 15.39) | 1.68 (0.09, 62.05) | 1.68 (0.47, 7.12) | 0.31 (0.05, 1.32) | 0.95 (0.46, 1.98) | 0.54 (0.23, 1.21) | 0.94 (0.56, 1.58) | 1.69 (0.04, 56.15) | 0.35 (0.01, 2.89) | 1.06 (0.24, 4.63) | 0.44 (0.08, 1.97) | 0.47 (0.04, 3.76) | **3.63 (1.84, 7.43)** | 1.29 (0.72, 2.34) | **5.72 (1.45, 32.04)** | 0.59 (0.06, 4.4) | 1.83 (0.75, 4.42) | 1.4 (0.56, 3.55) | 1.14 (0.27, 4.52) | 0.75 (0.15, 3.34) | **2.46 (1.4, 4.4)** | 1.49 (0.47, 4.76) | 1.28 (0.68, 2.39) | 2.3 (0.13, 39.22) | 1.28 (0.44, 3.94) | 1.31 (0.44, 3.97) | 0.99 (0.11, 8.86) | 1.85 (0.77, 4.67) | 2310.94 (0, 1.81308585916516e+30) | PenicillinV10d | |
| 0.67 (0.03, 11.57) | 1.11 (0.05, 47.13) | 1.11 (0.2, 6.54) | 0.2 (0.03, 1.15) | 0.61 (0.17, 2.28) | 0.35 (0.09, 1.38) | 0.61 (0.19, 1.9) | 1.09 (0.02, 41.94) | 0.23 (0.01, 2.46) | 0.68 (0.12, 3.76) | 0.28 (0.04, 1.7) | 0.3 (0.02, 3.06) | 2.36 (0.66, 8.57) | 0.84 (0.25, 2.83) | 3.71 (0.66, 26.93) | 0.38 (0.03, 3.75) | 1.19 (0.29, 4.73) | 0.91 (0.23, 3.68) | 0.73 (0.15, 3.47) | 0.49 (0.07, 3.07) | 1.6 (0.48, 5.47) | 0.97 (0.38, 2.44) | 0.84 (0.24, 2.8) | 1.49 (0.07, 29.64) | 0.83 (0.21, 3.35) | 0.85 (0.25, 2.94) | 0.64 (0.05, 7.97) | 1.2 (0.3, 4.96) | 1560.9 (0, 1.10354571774287e+30) | 0.65 (0.22, 1.89) | Telithromycin5d |

Values are presented as odds ratios (95% confidence intervals). Penicillin V 10d is used as reference. Statistically significant results are highlighted in bold. Abbreviations: AMC, amoxicillin-clavulanate; BPV, Benzathine Penicillin V.

**Supplementary Table 12. League table of pairwise comparisons for late clinical response in the pediatric subgroup**

| Amoxicillin |  |  |  |  |  |  |  |  |  |  |  |  |
| --- | --- | --- | --- | --- | --- | --- | --- | --- | --- | --- | --- | --- |
| 2.04 (0.47, 9.88) | AmoxicillinClavulanate | |  |  |  |  |  |  |  |  |  |  |
| 3.05 (0.78, 12.52) | 1.5 (0.38, 5.79) | Azithromycin |  |  |  |  |  |  |  |  |  |  |
| 1.42 (0.47, 5.15) | 0.7 (0.2, 2.58) | 0.47 (0.13, 1.75) | Cefaclor |  |  |  |  |  |  |  |  |  |
| 2.05 (0.29, 13.55) | 1.01 (0.14, 6.22) | 0.67 (0.12, 3.34) | 1.43 (0.19, 8.79) | Cefdinir |  |  |  |  |  |  |  |  |
| 2.11 (0.2, 22.67) | 1.02 (0.1, 10.63) | 0.69 (0.08, 6) | 1.46 (0.14, 15.03) | 1.02 (0.09, 12.76) | Cefixime |  |  |  |  |  |  |  |
| 1.37 (0.04, 24.21) | 0.68 (0.02, 10.81) | 0.45 (0.01, 6.66) | 0.97 (0.02, 15.85) | 0.68 (0.02, 13.45) | 0.66 (0.01, 17.5) | Cefprozil |  |  |  |  |  |  |
| 0.77 (0.12, 4.8) | 0.38 (0.05, 2.17) | 0.25 (0.05, 1.16) | 0.54 (0.08, 3.07) | 0.38 (0.05, 2.83) | 0.37 (0.03, 3.98) | 0.56 (0.03, 23.38) | CefuroximeAxetil |  |  |  |  |  |
| 0.72 (0.1, 4.73) | 0.36 (0.05, 2.14) | 0.24 (0.04, 1.17) | 0.51 (0.07, 3.07) | 0.35 (0.04, 2.8) | 0.34 (0.03, 3.9) | 0.52 (0.03, 22.6) | 0.93 (0.12, 6.91) | Cephalexin |  |  |  |  |
| 3.08 (0.74, 14.53) | 1.5 (0.43, 5.5) | 1.01 (0.32, 3.39) | 2.15 (0.51, 8.82) | 1.49 (0.3, 9.42) | 1.48 (0.16, 13.74) | 2.2 (0.18, 72.37) | 3.93 (0.85, 23.31) | 4.23 (0.85, 27.39) | Clarithromycin |  |  |  |
| 3.81 (0.47, 30.1) | 1.85 (0.22, 14.22) | 1.25 (0.19, 7.76) | 2.65 (0.31, 19.59) | 1.85 (0.21, 17.47) | 1.83 (0.13, 24.02) | 2.78 (0.12, 132.36) | 4.93 (0.59, 43.28) | 5.3 (0.58, 49.67) | 1.24 (0.17, 7.8) | Loracarbef |  |  |
| 2.07 (0.57, 6.98) | 1.02 (0.28, 3.2) | 0.68 (0.29, 1.41) | 1.44 (0.38, 4.52) | 1.01 (0.23, 4.38) | 0.99 (0.13, 6.92) | 1.46 (0.11, 48.52) | 2.68 (0.67, 10.6) | 2.86 (0.67, 12.85) | 0.68 (0.24, 1.51) | 0.55 (0.1, 2.78) | PenicillinV |  |
| 0.34 (0.02, 3.27) | 0.17 (0.01, 1.49) | **0.11 (0.01, 0.84)** | 0.23 (0.02, 2.14) | 0.16 (0.01, 1.83) | 0.16 (0.01, 2.47) | 0.24 (0.01, 12.41) | 0.44 (0.03, 4.53) | 0.47 (0.03, 5.31) | **0.11 (0.01, 0.84)** | 0.09 (0.01, 1.14) | 0.17 (0.02, 1.09) | Spiramycin |

Values are presented as odds ratios (95% confidence intervals). Penicillin V is used as reference. Statistically significant results are highlighted in bold.

**Supplementary Table 13. League table of pairwise comparisons for late clinical response in the adult subgroup**

| Amoxicillin |  |  |  |  |  |  |
| --- | --- | --- | --- | --- | --- | --- |
| 0.77 (0.03, 21.57) | Azithromycin |  |  |  |  |  |
| 0.57 (0.03, 11.8) | 0.74 (0.03, 16.85) | Cefotiam |  |  |  |  |
| 2.12 (0.05, 153.44) | 2.71 (0.06, 213.09) | 3.66 (0.1, 254.86) | CefpodoximeProxetil | |  |  |
| 0.79 (0.06, 10.92) | 1.02 (0.14, 7.61) | 1.37 (0.12, 17.64) | 0.39 (0.01, 10.18) | Clarithromycin |  |  |
| 0.5 (0.02, 10.63) | 0.64 (0.02, 14.79) | 0.87 (0.04, 17.48) | 0.24 (0, 9.1) | 0.63 (0.05, 7.08) | Loracarbef |  |
| 0.6 (0.07, 5.38) | 0.78 (0.07, 7.89) | 1.04 (0.13, 8.45) | 0.29 (0.01, 5.49) | 0.76 (0.19, 2.62) | 1.2 (0.14, 10.23) | PenicillinV |

Values are presented as odds ratios (95% confidence intervals). Penicillin V is used as reference.

**Supplementary Table 14. League table of pairwise comparisons for late clinical response stratified by treatment duration**

| Amoxicillin10d | |  |  |  |  |  |  |  |  |  |  |  |  |  |  |  |  |  |  |  |
| --- | --- | --- | --- | --- | --- | --- | --- | --- | --- | --- | --- | --- | --- | --- | --- | --- | --- | --- | --- | --- |
| 5.28 (0.53, 55.08) | Amoxicillin6d | |  |  |  |  |  |  |  |  |  |  |  |  |  |  |  |  |  |  |
| 10.31 (0.6, 208.96) | 1.94 (0.11, 41.73) | AmoxicillinClavulanate10d | | |  |  |  |  |  |  |  |  |  |  |  |  |  |  |  |  |
| 2.83 (0.35, 25.14) | 0.54 (0.06, 5.09) | 0.27 (0.02, 4.56) | AmoxicillinClavulanate5d | | |  |  |  |  |  |  |  |  |  |  |  |  |  |  |  |
| 5.31 (0.94, 34.1) | 1.01 (0.15, 7.09) | 0.52 (0.05, 4.95) | 1.88 (0.36, 10.02) | Azithromycin3d | |  |  |  |  |  |  |  |  |  |  |  |  |  |  |  |
| 3.62 (0.34, 43.56) | 0.69 (0.06, 8.94) | 0.35 (0.02, 7.24) | 1.29 (0.13, 13.4) | 0.69 (0.09, 5.12) | Azithromycin5d | |  |  |  |  |  |  |  |  |  |  |  |  |  |  |
| 5.29 (0.39, 79.03) | 0.99 (0.07, 16.06) | 0.51 (0.15, 1.66) | 1.87 (0.14, 24.35) | 0.99 (0.14, 6.97) | 1.44 (0.09, 24.11) | Cefaclor10d | |  |  |  |  |  |  |  |  |  |  |  |  |  |
| 1.07 (0.25, 4.57) | 0.2 (0.01, 3.14) | 0.1 (0, 2.46) | 0.38 (0.03, 4.78) | 0.2 (0.02, 1.92) | 0.29 (0.02, 4.7) | 0.2 (0.01, 3.91) | Cefaclor5d | |  |  |  |  |  |  |  |  |  |  |  |  |
| 1.71 (0.23, 11.47) | 0.32 (0.04, 2.35) | 0.17 (0.01, 2.22) | 0.61 (0.09, 3.39) | 0.32 (0.07, 1.19) | 0.47 (0.05, 3.58) | 0.32 (0.03, 3.31) | 1.61 (0.13, 17.03) | Cefdinir10d | |  |  |  |  |  |  |  |  |  |  |  |
| 3.17 (0.25, 42.76) | 0.59 (0.04, 8.65) | 0.3 (0.01, 7.2) | 1.12 (0.09, 13.31) | 0.59 (0.07, 5.49) | 0.87 (0.06, 13.2) | 0.59 (0.03, 11.3) | 2.97 (0.16, 58.4) | 1.85 (0.2, 20.75) | Cefixime10d | |  |  |  |  |  |  |  |  |  |  |
| 3 (0.33, 27.68) | 0.56 (0.06, 5.63) | 0.29 (0.01, 4.87) | 1.06 (0.12, 8.32) | 0.57 (0.09, 3.09) | 0.83 (0.07, 8.7) | 0.57 (0.04, 7.4) | 2.83 (0.2, 40) | 1.76 (0.27, 12.39) | 0.95 (0.07, 11.58) | Cefotiam5d | |  |  |  |  |  |  |  |  |  |
| 10.9 (0.52, 467.5) | 2.06 (0.09, 92.03) | 1.07 (0.03, 66.89) | 3.84 (0.19, 155.78) | 2.02 (0.13, 69.92) | 3 (0.12, 136.97) | 2.09 (0.07, 111.34) | 10.38 (0.35, 556.18) | 6.42 (0.38, 237.08) | 3.53 (0.12, 178.21) | 3.64 (0.18, 154.27) | CefpodoximeProxetil5d | | |  |  |  |  |  |  |  |
| 1.77 (0.04, 41.18) | 0.34 (0.01, 8.05) | 0.17 (0, 6.28) | 0.62 (0.02, 12.84) | 0.34 (0.01, 5.26) | 0.49 (0.01, 9.53) | 0.33 (0.01, 10.05) | 1.65 (0.03, 53.82) | 1.05 (0.03, 20.01) | 0.55 (0.01, 15.93) | 0.59 (0.02, 13.58) | 0.15 (0, 7.29) | Cefprozil5d | |  |  |  |  |  |  |  |
| 1.17 (0.14, 9.75) | 0.22 (0.02, 2.01) | 0.11 (0.01, 1.76) | 0.41 (0.05, 2.94) | 0.22 (0.04, 1.05) | 0.32 (0.03, 3.02) | 0.22 (0.02, 2.64) | 1.1 (0.08, 14.17) | 0.68 (0.12, 4.38) | 0.37 (0.03, 4.13) | 0.39 (0.05, 3.13) | 0.11 (0, 2.05) | 0.65 (0.03, 23.47) | CefuroximeAxetil10d | |  |  |  |  |  |  |
| 1.09 (0.12, 9.61) | 0.21 (0.02, 1.96) | 0.11 (0.01, 1.73) | 0.39 (0.05, 2.92) | 0.21 (0.03, 1.07) | 0.3 (0.03, 2.99) | 0.21 (0.01, 2.59) | 1.03 (0.07, 13.64) | 0.64 (0.1, 4.4) | 0.35 (0.03, 4.16) | 0.36 (0.04, 3.08) | 0.1 (0, 2.03) | 0.62 (0.03, 22.68) | 0.94 (0.12, 7.22) | Cephalexin10d | |  |  |  |  |  |
| 3.72 (0.55, 28.25) | 0.71 (0.09, 5.77) | 0.36 (0.02, 5.18) | 1.33 (0.21, 8.42) | 0.7 (0.17, 2.92) | 1.03 (0.24, 4.29) | 0.71 (0.06, 7.87) | 3.5 (0.32, 42.2) | 2.18 (0.48, 12.34) | 1.19 (0.11, 12.19) | 1.25 (0.19, 9.11) | 0.35 (0.01, 6.16) | 2.06 (0.16, 58.89) | 3.2 (0.56, 20.64) | 3.41 (0.55, 24.07) | Clarithromycin10d | |  |  |  |  |
| 4.22 (0.78, 26.52) | 0.8 (0.12, 5.52) | 0.42 (0.03, 5.12) | 1.5 (0.37, 6.42) | 0.8 (0.26, 2.49) | 1.17 (0.16, 8.51) | 0.81 (0.08, 7.61) | 3.98 (0.43, 41.49) | 2.47 (0.72, 11.05) | 1.36 (0.15, 11.81) | 1.42 (0.27, 8.52) | 0.4 (0.01, 6.05) | 2.37 (0.16, 72.37) | 3.62 (0.8, 19.07) | 3.86 (0.79, 22.62) | 1.14 (0.29, 4.53) | Clarithromycin5d | |  |  |  |
| 11.23 (0.46, 317.56) | 2.11 (0.08, 63.74) | 1.09 (0.25, 4.76) | 3.95 (0.17, 100.28) | 2.1 (0.14, 33.15) | 3.06 (0.11, 96.05) | 2.12 (0.32, 14.9) | 10.52 (0.32, 414.88) | 6.52 (0.34, 164.2) | 3.55 (0.11, 125.42) | 3.75 (0.16, 106.03) | 1 (0.01, 53.55) | 6.54 (0.13, 503.73) | 9.58 (0.43, 252.91) | 10.25 (0.44, 285.25) | 2.98 (0.14, 68.5) | 2.61 (0.14, 51.82) | Clindamycin10d | |  |  |
| 4.04 (0.65, 26.6) | 0.76 (0.11, 5.53) | 0.4 (0.03, 5.21) | 1.43 (0.25, 7.87) | 0.76 (0.2, 2.68) | 1.12 (0.13, 8.49) | 0.77 (0.07, 7.67) | 3.81 (0.37, 40.66) | 2.37 (0.57, 11.38) | 1.29 (0.13, 11.85) | 1.35 (0.22, 8.6) | 0.38 (0.01, 6.07) | 2.26 (0.13, 72.16) | 3.48 (0.64, 19.42) | 3.71 (0.64, 22.78) | 1.08 (0.23, 4.78) | 0.95 (0.26, 3.14) | 0.36 (0.02, 7.01) | Loracarbef10d | |  |
| 3.12 (0.65, 15.57) | 0.59 (0.1, 3.24) | 0.31 (0.02, 3.29) | 1.11 (0.25, 4.48) | 0.59 (0.24, 1.31) | 0.87 (0.13, 5.11) | 0.59 (0.07, 4.78) | 2.95 (0.34, 25.04) | 1.83 (0.63, 5.98) | 0.99 (0.12, 7.2) | 1.04 (0.22, 4.91) | 0.29 (0.01, 3.88) | 1.72 (0.12, 48.76) | 2.69 (0.66, 10.86) | 2.85 (0.65, 13.04) | 0.84 (0.25, 2.53) | 0.74 (0.32, 1.47) | 0.28 (0.01, 4.54) | 0.77 (0.28, 2.04) | PenicillinV10d | |
| 2.62 (0.36, 20.08) | 0.5 (0.06, 4.07) | 0.25 (0.02, 3.71) | 0.93 (0.14, 5.99) | 0.49 (0.11, 2.12) | 0.72 (0.11, 4.47) | 0.5 (0.04, 5.58) | 2.45 (0.21, 29.92) | 1.53 (0.31, 8.84) | 0.83 (0.07, 8.58) | 0.88 (0.12, 6.49) | 0.24 (0.01, 4.34) | 1.45 (0.09, 44.74) | 2.25 (0.36, 14.76) | 2.39 (0.36, 17.46) | 0.7 (0.21, 2.24) | 0.62 (0.14, 2.5) | 0.23 (0.01, 4.87) | 0.64 (0.13, 3.15) | 0.84 (0.25, 2.91) | Telithromycin5d |

Values are presented as odds ratios (95% confidence intervals). Penicillin V 10d is used as reference.

**Supplementary Table 15. Sensitivity analysis of pairwise comparisons for early bacterial eradication in post-2005 studies**

| AMC |  |  |  |  |  |  |
| --- | --- | --- | --- | --- | --- | --- |
| 0.31 (0.07, 1.31) | Amoxicillin |  |  |  |  |  |
| 0.14 (0.02, 1.17) | 0.46 (0.1, 2.18) | Azithromycin |  |  |  |  |
| 0.26 (0.03, 2.03) | 0.86 (0.2, 3.62) | 1.84 (0.38, 9.15) | Cefaclor |  |  |  |
| 0.22 (0.02, 1.98) | 0.72 (0.14, 3.63) | 1.54 (0.16, 14.66) | 0.84 (0.09, 7.52) | CefcapenePivoxil |  |  |
| 0.34 (0.07, 1.44) | 1.11 (0.14, 8.77) | 2.39 (0.18, 31.64) | 1.29 (0.1, 16.07) | 1.55 (0.11, 21.23) | Clindamycin |  |
| 0.28 (0.05, 1.53) | 0.92 (0.4, 2.2) | 1.98 (0.35, 11.85) | 1.08 (0.21, 5.81) | 1.29 (0.21, 8.21) | 0.83 (0.09, 8.15) | PenicillinV |

Values are presented as odds ratios (95% confidence intervals). Penicillin V is used as reference. Abbreviations: AMC, amoxicillin-clavulanate.

**Supplementary Table 16. Sensitivity analysis of pairwise comparisons for late bacterial eradication in post-2005 studies**

| Amoxicillin |  |  |  |  |
| --- | --- | --- | --- | --- |
| 0.4 (0.04, 2.82) | Azithromycin |  |  |  |
| 1.17 (0.51, 2.63) | 2.93 (0.35, 33.78) | BenzathinePenicillinG | |  |
| 1.24 (0.25, 6.65) | 3.1 (0.5, 28.99) | 1.07 (0.17, 6.84) | Cefaclor |  |
| 0.91 (0.45, 1.81) | 2.27 (0.29, 24.33) | 0.77 (0.27, 2.24) | 0.73 (0.12, 4.12) | PenicillinV |

Values are presented as odds ratios (95% confidence intervals). Penicillin V is used as reference.

**Supplementary Table 17. Sensitivity analysis of pairwise comparisons for early clinical response in post-2005 studies**

| AMC |  |  |  |  |  |  |
| --- | --- | --- | --- | --- | --- | --- |
| 5.16 (0.37, 158.06) | Amoxicillin |  |  |  |  |  |
| 1.77 (0.06, 86.11) | 0.34 (0.04, 2.21) | Azithromycin |  |  |  |  |
| 4.29 (0.18, 190.08) | 0.81 (0.13, 4.74) | 2.38 (0.34, 19.51) | Cefaclor |  |  |  |
| 3.67 (0.06, 312.41) | 0.66 (0.03, 13.28) | 1.96 (0.2, 23.43) | 0.81 (0.04, 17.6) | CefcapenePivoxil |  |  |
| 0.45 (0.1, 2.08) | 0.09 (0, 1.78) | 0.25 (0, 10.11) | 0.1 (0, 3.58) | 0.12 (0, 9.66) | Clindamycin |  |
| 2.81 (0.14, 114.55) | 0.54 (0.12, 2.49) | 1.58 (0.14, 21.02) | 0.66 (0.06, 7.06) | 0.81 (0.03, 24.66) | 6.31 (0.22, 335.42) | PenicillinV |

Values are presented as odds ratios (95% confidence intervals). Penicillin V is used as reference. Abbreviations: AMC, amoxicillin-clavulanate.

**Supplementary Table 18. League table of pairwise comparisons for early bacteriological recurrence**

| **Amoxicillin** |  |  |  |  |  |  |  |  |  |  |
| --- | --- | --- | --- | --- | --- | --- | --- | --- | --- | --- |
| 1.28 (0.1, 16.39) | **Azithromycin** |  |  |  |  |  |  |  |  |  |
| 0.85 (0.06, 10.88) | 0.66 (0.05, 8.58) | **Benzathine Penicillin V** |  |  |  |  |  |  |  |  |
| 0.95 (0.02, 62.84) | 0.76 (0.01, 50.96) | 1.13 (0.02, 76.75) | **Cefetamet Pivoxil** |  |  |  |  |  |  |  |
| 5.3 (0.36, 89.15) | 4.15 (0.27, 70.76) | 6.22 (0.41, 105.47) | 5.58 (0.07, 407.47) | **Cefixime** |  |  |  |  |  |  |
| 30287.31 (0.32, 25906475248672.6) | 24073.86 (0.24, 20128475897778.9) | 37027.98 (0.37, 28923953659982.6) | 34952.7 (0.16, 23636781740362.5) | 5838.77 (0.05, 4770550083374.78) | **Clarithromycin** |  |  |  |  |  |
| 1.5 (0.11, 19.5) | 1.17 (0.09, 15.71) | 1.76 (0.13, 23.66) | 1.56 (0.02, 98.26) | 0.28 (0.02, 4.37) | 0 (0, 3.46) | **Dirithromycin** |  |  |  |  |
| 1.14 (0.05, 27.71) | 0.89 (0.04, 22.16) | 1.34 (0.05, 32.78) | 1.2 (0.01, 112.92) | 0.22 (0.01, 5.87) | 0 (0, 2.03) | 0.76 (0.11, 5) | **Erythromycin** |  |  |  |
| 1.52 (0.17, 14.36) | 1.2 (0.13, 11.43) | 1.78 (0.2, 17.43) | 1.59 (0.03, 85.95) | 0.29 (0.02, 3.24) | 0 (0, 4.53) | 1.01 (0.11, 10.06) | 1.34 (0.07, 26.51) | **Loracarbef** |  |  |
| 1.01 (0.16, 6.11) | 0.79 (0.13, 4.87) | 1.18 (0.19, 7.43) | 1.06 (0.02, 44.82) | 0.19 (0.02, 1.44) | 0 (0, 2.67) | 0.67 (0.1, 4.31) | 0.89 (0.06, 12.67) | 0.66 (0.17, 2.39) | **PenicillinV** |  |
| 51025.13 (0.42, 46617522234025.8) | 40536.25 (0.32, 38984047626811.4) | 61312.66 (0.48, 50553109285848.7) | 59133.38 (0.23, 42812110890705.1) | 9908.73 (0.07, 8602748016889.33) | 1.62 (0.22, 12.05) | 32828.1 (0.39, 29691747983459.1) | 41311.16 (0.66, 40810791822361.8) | 33327.84 (0.29, 29521936036733.9) | 50259.32 (0.49, 42628237712115.4) | **Telithromycin** |

Values are presented as odds ratios (95% confidence intervals). Penicillin V is used as reference. Extremely wide or undefined confidence intervals may occur when one treatment arm had 0% or 100% event rates.

**Supplementary Table 19**. League table of pairwise comparisons for late bacteriological recurrence

| **AMC** |  |  |  |  |  |  |  |  |  |  |  |  |  |  |  |
| --- | --- | --- | --- | --- | --- | --- | --- | --- | --- | --- | --- | --- | --- | --- | --- |
| 0.32 (0.05, 1.81) | **Amoxicillin** |  |  |  |  |  |  |  |  |  |  |  |  |  |  |
| **0.17 (0.03, 0.88)** | 0.54 (0.24, 1.31) | **Azithromycin** |  |  |  |  |  |  |  |  |  |  |  |  |  |
| 0.66 (0.11, 3.13) | 2.07 (0.59, 7.49) | **3.8 (1.39, 10.96)** | **Cefaclor** |  |  |  |  |  |  |  |  |  |  |  |  |
| 1.06 (0.07, 17.82) | 3.38 (0.38, 36.63) | 6.19 (0.7, 64.62) | 1.62 (0.15, 20.3) | **Cefixime** |  |  |  |  |  |  |  |  |  |  |  |
| 1.97 (0.12, 81.85) | 6.05 (0.58, 197.14) | **11.11 (1.11, 363.98)** | 2.97 (0.24, 102.23) | 1.87 (0.07, 94.93) | **Cefotiam** |  |  |  |  |  |  |  |  |  |  |
| 0.42 (0.06, 2.39) | 1.32 (0.53, 3.33) | **2.46 (1.03, 5.57)** | 0.64 (0.18, 2.2) | 0.4 (0.04, 3.51) | 0.22 (0.01, 2.23) | **Cefpodoxime Proxetil** |  |  |  |  |  |  |  |  |  |
| 0.72 (0.1, 4.56) | 2.28 (0.7, 7.71) | **4.25 (1.43, 12.9)** | 1.12 (0.29, 4.14) | 0.68 (0.06, 6.98) | 0.38 (0.01, 4.53) | 1.72 (0.54, 5.74) | **Cefprozil** |  |  |  |  |  |  |  |  |
| 0.29 (0.01, 5.17) | 0.94 (0.08, 10.72) | 1.74 (0.15, 18.93) | 0.45 (0.03, 6.14) | 0.28 (0.01, 6.66) | 0.15 (0, 4.22) | 0.72 (0.06, 7.97) | 0.41 (0.03, 5.1) | **Cefuroxime Axetil** |  |  |  |  |  |  |  |
| 0.31 (0.05, 1.66) | 0.97 (0.43, 2.28) | 1.81 (0.84, 3.72) | 0.47 (0.14, 1.49) | 0.29 (0.03, 2.49) | 0.16 (0.01, 1.6) | 0.74 (0.32, 1.68) | 0.43 (0.15, 1.17) | 1.03 (0.09, 12.07) | **Clarithromycin** |  |  |  |  |  |  |
| 0.44 (0.06, 2.41) | 1.36 (0.47, 3.98) | 2.54 (0.94, 6.57) | 0.66 (0.18, 2.33) | 0.41 (0.04, 3.87) | 0.22 (0.01, 2.47) | 1.03 (0.35, 2.96) | 0.59 (0.17, 2.1) | 1.45 (0.12, 18.11) | 1.41 (0.52, 3.71) | **Dirithromycin** |  |  |  |  |  |
| 0.46 (0.08, 2.12) | 1.44 (0.56, 3.89) | **2.68 (1.22, 5.87)** | 0.7 (0.24, 1.99) | 0.43 (0.04, 3.95) | 0.24 (0.01, 2.44) | 1.09 (0.43, 2.87) | 0.63 (0.21, 1.87) | 1.55 (0.14, 18.27) | 1.49 (0.63, 3.52) | 1.06 (0.44, 2.62) | **Erythromycin** |  |  |  |  |
| 0.39 (0.05, 2.29) | 1.22 (0.47, 3.27) | 2.28 (0.9, 5.5) | 0.59 (0.16, 2.09) | 0.37 (0.03, 3.32) | 0.2 (0.01, 2.1) | 0.93 (0.36, 2.41) | 0.54 (0.16, 1.78) | 1.29 (0.11, 15.9) | 1.26 (0.52, 3.03) | 0.9 (0.3, 2.72) | 0.85 (0.31, 2.29) | **Loracarbef** |  |  |  |
| 0.36 (0.06, 1.78) | 1.12 (0.59, 2.18) | **2.08 (1.18, 3.53)** | 0.54 (0.18, 1.55) | 0.34 (0.03, 2.71) | 0.19 (0.01, 1.71) | 0.85 (0.45, 1.6) | 0.49 (0.18, 1.29) | 1.19 (0.11, 13.04) | 1.16 (0.68, 1.92) | 0.82 (0.35, 1.92) | 0.78 (0.38, 1.56) | 0.91 (0.45, 1.87) | **Penicillin V** |  |  |
| 0.15 (0.01, 1.23) | 0.47 (0.1, 2.03) | 0.87 (0.19, 3.53) | 0.22 (0.04, 1.27) | 0.14 (0.01, 1.68) | 0.08 (0, 1.15) | 0.36 (0.08, 1.52) | 0.2 (0.04, 1.07) | 0.5 (0.03, 7.68) | 0.49 (0.11, 1.94) | 0.35 (0.07, 1.64) | 0.33 (0.07, 1.42) | 0.39 (0.08, 1.7) | 0.42 (0.1, 1.54) | **Spiramycin** |  |
| 0.2 (0.02, 1.65) | 0.63 (0.14, 2.87) | 1.18 (0.25, 4.96) | 0.31 (0.05, 1.65) | 0.19 (0.01, 2.28) | 0.1 (0, 1.42) | 0.48 (0.1, 2.15) | 0.28 (0.05, 1.37) | 0.66 (0.05, 10.94) | 0.65 (0.17, 2.26) | 0.46 (0.09, 2.33) | 0.44 (0.09, 1.97) | 0.52 (0.11, 2.42) | 0.56 (0.14, 2.2) | 1.34 (0.2, 9.49) | **Telithromycin** |

Values are presented as odds ratios (95% confidence intervals). Penicillin V is used as reference. Statistically significant results are highlighted in bold. Abbreviations: AMC, amoxicillin-clavulanate.

**Supplementary Table 20**. League table of pairwise comparisons for overall adverse events

| **AMC** |  |  |  |  |  |  |  |  |  |  |  |  |  |  |  |  |
| --- | --- | --- | --- | --- | --- | --- | --- | --- | --- | --- | --- | --- | --- | --- | --- | --- |
| 3.06 (0.98, 9.75) | **Amoxicillin** |  |  |  |  |  |  |  |  |  |  |  |  |  |  |  |
| 0.62 (0.22, 1.77) | **0.2 (0.07, 0.61)** | **Azithromycin** |  |  |  |  |  |  |  |  |  |  |  |  |  |  |
| 2.68 (0.5, 14.19) | 0.87 (0.16, 4.74) | 4.31 (0.87, 20.62) | **BPV** |  |  |  |  |  |  |  |  |  |  |  |  |  |
| 2.48 (0.78, 8) | 0.81 (0.29, 2.14) | **4 (1.16, 13.7)** | 0.93 (0.15, 5.69) | **Cefaclor** |  |  |  |  |  |  |  |  |  |  |  |  |
| 1.63 (0.59, 4.78) | 0.54 (0.18, 1.59) | **2.63 (1.1, 6.42)** | 0.61 (0.13, 2.97) | 0.66 (0.19, 2.32) | **Cefdinir** |  |  |  |  |  |  |  |  |  |  |  |
| 2.83 (0.48, 17.33) | 0.92 (0.15, 5.85) | 4.55 (0.84, 25.65) | 1.06 (0.13, 9.24) | 1.14 (0.17, 8.06) | 1.73 (0.32, 9.46) | **Cefixime** |  |  |  |  |  |  |  |  |  |  |
| 4.25 (1.03, 18.05) | 1.39 (0.32, 5.99) | **6.85 (1.81, 25.88)** | 1.59 (0.26, 10.25) | 1.72 (0.35, 8.48) | 2.6 (0.71, 9.5) | 1.5 (0.21, 10.41) | **Cefotiam** |  |  |  |  |  |  |  |  |  |
| 1.58 (0.38, 6.68) | 0.52 (0.12, 2.22) | 2.56 (0.67, 9.43) | 0.59 (0.1, 3.82) | 0.64 (0.13, 3.12) | 0.97 (0.26, 3.48) | 0.56 (0.08, 3.84) | 0.37 (0.07, 1.88) | **Cefpodoxime Proxetil** |  |  |  |  |  |  |  |  |
| 1.63 (0.33, 8.08) | 0.53 (0.1, 2.88) | 2.62 (0.54, 12.62) | 0.61 (0.07, 5.04) | 0.66 (0.12, 3.61) | 1 (0.19, 5.05) | 0.57 (0.06, 5.08) | 0.38 (0.06, 2.59) | 1.03 (0.15, 6.93) | **Cefprozil** |  |  |  |  |  |  |  |
| 1.4 (0.37, 5.49) | 0.46 (0.11, 1.82) | 2.25 (0.65, 7.71) | 0.52 (0.09, 3.19) | 0.56 (0.12, 2.6) | 0.85 (0.26, 2.84) | 0.49 (0.07, 3.26) | 0.33 (0.07, 1.57) | 0.88 (0.19, 4.25) | 0.85 (0.14, 5.57) | **Cefuroxime Axetil** |  |  |  |  |  |  |
| 1.5 (0.63, 3.5) | 0.49 (0.18, 1.29) | **2.41 (1.12, 5.13)** | 0.56 (0.13, 2.56) | 0.61 (0.19, 1.84) | 0.92 (0.43, 1.87) | 0.53 (0.1, 2.67) | 0.35 (0.1, 1.19) | 0.95 (0.28, 3.21) | 0.92 (0.2, 4.2) | 1.07 (0.34, 3.3) | **Clarithromycin** |  |  |  |  |  |
| 0.91 (0.34, 2.44) | **0.3 (0.09, 0.94)** | 1.46 (0.54, 3.89) | 0.34 (0.06, 1.87) | 0.37 (0.11, 1.17) | 0.56 (0.19, 1.59) | 0.32 (0.05, 1.91) | **0.21 (0.05, 0.91)** | 0.58 (0.13, 2.44) | 0.56 (0.16, 1.91) | 0.65 (0.16, 2.55) | 0.61 (0.25, 1.47) | **Erythromycin** |  |  |  |  |
| 2.13 (0.74, 6.38) | 0.7 (0.23, 2.13) | **3.44 (1.38, 8.65)** | 0.8 (0.17, 3.99) | 0.86 (0.24, 3.1) | 1.31 (0.53, 3.15) | 0.76 (0.13, 4.13) | 0.5 (0.13, 1.89) | 1.35 (0.37, 5.09) | 1.31 (0.25, 6.93) | 1.53 (0.44, 5.22) | 1.42 (0.67, 3.17) | 2.34 (0.79, 7.26) | **Loracarbef** |  |  |  |
| 2.3 (0.98, 5.49) | 0.75 (0.3, 1.84) | **3.69 (1.93, 7.13)** | 0.85 (0.21, 3.72) | 0.93 (0.31, 2.78) | 1.4 (0.77, 2.53) | 0.81 (0.16, 3.92) | 0.54 (0.17, 1.71) | 1.45 (0.47, 4.63) | 1.41 (0.31, 6.56) | 1.64 (0.58, 4.68) | **1.53 (1.01, 2.4)** | **2.52 (1.04, 6.26)** | 1.07 (0.56, 2.05) | **PenicillinV** |  |  |
| 2.8 (0.67, 12.15) | 0.92 (0.21, 4.04) | **4.51 (1.19, 17.23)** | 1.05 (0.17, 6.83) | 1.13 (0.23, 5.71) | 1.71 (0.46, 6.33) | 0.99 (0.14, 7.06) | 0.66 (0.13, 3.45) | 1.77 (0.35, 9.25) | 1.72 (0.26, 11.96) | 2.01 (0.42, 9.65) | 1.87 (0.55, 6.62) | 3.08 (0.72, 13.62) | 1.31 (0.35, 5.04) | 1.22 (0.38, 3.96) | **Spiramycin** |  |
| 1.26 (0.42, 3.79) | 0.41 (0.13, 1.31) | 2.02 (0.75, 5.34) | 0.47 (0.09, 2.4) | 0.51 (0.14, 1.87) | 0.77 (0.29, 1.97) | 0.44 (0.08, 2.5) | 0.3 (0.07, 1.16) | 0.79 (0.2, 3.13) | 0.77 (0.15, 4.11) | 0.9 (0.24, 3.23) | 0.84 (0.4, 1.79) | 1.38 (0.45, 4.28) | 0.59 (0.21, 1.58) | 0.55 (0.25, 1.15) | 0.45 (0.11, 1.78) | **Telithromycin** |

Values are presented as odds ratios (95% confidence intervals). Penicillin V is used as reference. Statistically significant results are highlighted in bold. Abbreviations: AMC, amoxicillin-clavulanate; BPV, Benzathine Penicillin V.

**Supplementary Table 21**. League table of pairwise comparisons for diarrhea (adverse event)

| **AMC** |  |  |  |  |  |  |  |  |  |  |  |  |  |  |  |  |  |
| --- | --- | --- | --- | --- | --- | --- | --- | --- | --- | --- | --- | --- | --- | --- | --- | --- | --- |
| 3.61 (0.74, 17.36) | **Amoxicillin** |  |  |  |  |  |  |  |  |  |  |  |  |  |  |  |  |
| **8.42 (1.44, 60.02)** | 2.33 (0.46, 14.99) | **Azithromycin** |  |  |  |  |  |  |  |  |  |  |  |  |  |  |  |
| **30.24 (1.18, 992.39)** | 8.35 (0.35, 256.6) | 3.57 (0.18, 79.07) | **BPV** |  |  |  |  |  |  |  |  |  |  |  |  |  |  |
| **6.71 (1.66, 34.24)** | 1.86 (0.43, 9.94) | 0.8 (0.17, 3.7) | 0.22 (0.01, 5.53) | **Cefaclor** |  |  |  |  |  |  |  |  |  |  |  |  |  |
| 4.08 (0.21, 75.91) | 1.13 (0.09, 13.76) | 0.48 (0.02, 9.24) | 0.13 (0, 7.46) | 0.6 (0.03, 10.61) | **CefcapenePivoxil** |  |  |  |  |  |  |  |  |  |  |  |  |
| **9.6 (1.22, 121.76)** | 2.65 (0.38, 30.68) | 1.14 (0.22, 7.98) | 0.32 (0.02, 6.02) | 1.43 (0.19, 13.78) | 2.39 (0.11, 83.72) | **Cefdinir** |  |  |  |  |  |  |  |  |  |  |  |
| 27.95 (0.35, 2776.97) | 7.7 (0.1, 757.19) | 3.28 (0.05, 252.98) | 0.91 (0.01, 115.97) | 4.09 (0.05, 367.69) | 6.92 (0.05, 1251.85) | 2.81 (0.05, 176.84) | **Cefixime** |  |  |  |  |  |  |  |  |  |  |
| **121.04 (6.46, 4190.87)** | **33.45 (1.91, 1081.71)** | 14.26 (0.99, 328.73) | 4.05 (0.12, 177.91) | 17.85 (0.99, 502.04) | 30.06 (0.69, 2228.22) | 12.31 (0.96, 210.26) | 4.49 (0.04, 504.22) | **Cefotiam** |  |  |  |  |  |  |  |  |  |
| 1.24 (0.06, 20.01) | 0.34 (0.02, 6.32) | 0.15 (0.01, 2.24) | 0.04 (0, 1.77) | 0.18 (0.01, 2.85) | 0.31 (0.01, 13.96) | 0.13 (0, 2.17) | 0.04 (0, 5.13) | **0.01 (0, 0.34)** | **Cefprozil** |  |  |  |  |  |  |  |  |
| 7.44 (0.46, 184.97) | 2.05 (0.14, 47.9) | 0.88 (0.07, 13.8) | 0.25 (0.01, 7.91) | 1.1 (0.07, 22.08) | 1.85 (0.05, 107.3) | 0.77 (0.07, 8.38) | 0.28 (0, 25.22) | 0.06 (0, 1.53) | 5.99 (0.21, 337.79) | **Cefuroxime Axetil** |  |  |  |  |  |  |  |
| **10.49 (1.04, 150.14)** | 2.88 (0.32, 38.32) | 1.24 (0.18, 10.28) | 0.35 (0.02, 7.37) | 1.55 (0.16, 17.38) | 2.58 (0.09, 99.3) | 1.09 (0.18, 5.82) | 0.38 (0.01, 25.34) | 0.09 (0, 1.29) | 8.36 (0.42, 296.87) | 1.41 (0.1, 17.73) | **Clarithromycin** |  |  |  |  |  |  |
| 6.84 (0.81, 64.32) | 1.89 (0.22, 18.5) | 0.82 (0.11, 5.45) | 0.23 (0.01, 4.94) | 1.02 (0.11, 8.08) | 1.69 (0.06, 49.91) | 0.72 (0.08, 4.15) | 0.25 (0, 16.83) | **0.06 (0, 0.87)** | 5.48 (0.39, 104.39) | 0.93 (0.05, 11.82) | 0.66 (0.06, 5.05) | **Dirithromycin** |  |  |  |  |  |
| 2.76 (0.4, 17.19) | 0.77 (0.09, 5.68) | 0.33 (0.04, 1.95) | 0.09 (0, 2.16) | 0.41 (0.05, 2.46) | 0.69 (0.03, 16.26) | 0.29 (0.02, 2.03) | 0.1 (0, 7.38) | **0.02 (0, 0.39)** | 2.23 (0.25, 21.02) | 0.38 (0.01, 5.31) | 0.27 (0.02, 2.35) | 0.41 (0.06, 2.03) | **Erythromycin** |  |  |  |  |
| **32.04 (2.85, 559)** | 8.86 (0.88, 143.26) | 3.77 (0.48, 39.2) | 1.06 (0.05, 25.81) | 4.71 (0.45, 64.37) | 7.98 (0.27, 343.58) | 3.3 (0.5, 22.59) | 1.18 (0.02, 84.29) | 0.27 (0.01, 4.59) | **25.52 (1.2, 1095.24)** | 4.29 (0.29, 64.66) | 3.04 (0.38, 27.8) | 4.62 (0.55, 59.87) | **11.43 (1.17, 212.65)** | **Loracarbef** |  |  |  |
| 3.83 (0.11, 124.29) | 1.06 (0.03, 35.31) | 0.46 (0.01, 11.86) | 0.13 (0, 6.99) | 0.56 (0.02, 16.47) | 0.93 (0.01, 72.65) | 0.39 (0.01, 9.1) | 0.14 (0, 19.23) | 0.03 (0, 1.39) | 3.08 (0.07, 164.81) | 0.51 (0.01, 19.79) | 0.36 (0.01, 10.07) | 0.56 (0.03, 8) | 1.38 (0.06, 36.76) | 0.12 (0, 3.48) | **Miocamycin** |  |  |
| **27.12 (4.27, 271.16)** | **7.51 (1.35, 67.83)** | 3.21 (0.85, 16.48) | 0.9 (0.07, 14.11) | 4.01 (0.7, 30.06) | 6.73 (0.34, 199.89) | 2.81 (1.03, 7.83) | 1.01 (0.02, 52.51) | 0.23 (0.02, 2.38) | **21.69 (1.52, 611.92)** | 3.64 (0.42, 32.11) | 2.57 (0.67, 11.47) | 3.89 (0.89, 27.34) | **9.71 (1.72, 101.93)** | 0.85 (0.17, 4.23) | 7.13 (0.35, 225.67) | **PenicillinV** |  |
| 4.67 (0.43, 77.45) | 1.29 (0.13, 19.63) | 0.55 (0.07, 5.4) | 0.16 (0.01, 3.58) | 0.69 (0.07, 9) | 1.15 (0.04, 48.23) | 0.49 (0.07, 3.08) | 0.17 (0, 12) | **0.04 (0, 0.65)** | 3.72 (0.18, 149.98) | 0.63 (0.04, 9.04) | 0.45 (0.1, 2.14) | 0.67 (0.08, 8.31) | 1.66 (0.18, 29.53) | 0.15 (0.01, 1.33) | 1.23 (0.04, 54.41) | **0.17 (0.03, 0.81)** | **Telithromycin** |

Values are presented as odds ratios (95% confidence intervals). Penicillin V is used as reference. Statistically significant results are highlighted in bold. Abbreviations: AMC, amoxicillin-clavulanate; BPV, Benzathine Penicillin V.

**Supplementary Table 22. League table of pairwise comparisons for** vomiting (adverse event)

| **AMC** |  |  |  |  |  |  |  |  |  |  |  |  |  |  |
| --- | --- | --- | --- | --- | --- | --- | --- | --- | --- | --- | --- | --- | --- | --- |
| 0.57 (0, 66.53) | **Amoxicillin** |  |  |  |  |  |  |  |  |  |  |  |  |  |
| 0.86 (0, 92.8) | 1.52 (0.03, 97.09) | **Azithromycin** |  |  |  |  |  |  |  |  |  |  |  |  |
| 0.99 (0.01, 33.06) | 1.75 (0.05, 71.51) | 1.16 (0.03, 37.51) | **Cefaclor** |  |  |  |  |  |  |  |  |  |  |  |
| 1.35 (0, 389.29) | 2.41 (0.02, 256.25) | 1.58 (0.01, 132.21) | 1.37 (0.01, 192) | **Cefdinir** |  |  |  |  |  |  |  |  |  |  |
| 12773947362.72 (0.55, 5.49629415691838e+30) | **25399459817 (1.69, 8.83093500503887e+30)** | **15977827363.51 (1.1, 5.96978875043854e+30)** | 14351397797.41 (0.89, 5.64732580654959e+30) | 10197490746.17 (0.73, 3.59285952416531e+30) | **Cefixime** |  |  |  |  |  |  |  |  |  |
| 1.04 (0, 551.93) | 1.86 (0, 943.33) | 1.22 (0, 352.74) | 1.06 (0, 362.82) | 0.77 (0, 631.4) | 0 (0, 2.25) | **Cefprozil** |  |  |  |  |  |  |  |  |
| 0.15 (0, 150.47) | 0.28 (0, 119.27) | 0.18 (0, 64.96) | 0.16 (0, 85.63) | 0.12 (0, 56.23) | **0 (0, 0.26)** | 0.15 (0, 358) | **CefuroximeAxetil** |  |  |  |  |  |  |  |
| 0.34 (0, 52.16) | 0.61 (0.01, 39.41) | 0.4 (0.01, 17.26) | 0.35 (0, 24.48) | 0.25 (0, 22.32) | **0 (0, 0.32)** | 0.33 (0, 123.65) | 2.17 (0.01, 1115.83) | **Clarithromycin** |  |  |  |  |  |  |
| 0.94 (0, 182.75) | 1.66 (0.01, 187.85) | 1.11 (0.01, 74.17) | 0.95 (0.01, 93.42) | 0.7 (0, 114.42) | 0 (0, 1.08) | 0.91 (0, 378.48) | 6.09 (0.01, 5071.29) | 2.77 (0.03, 275.35) | **Dirithromycin** |  |  |  |  |  |
| 0.48 (0, 32.89) | 0.84 (0.01, 51.21) | 0.55 (0.02, 13.39) | 0.48 (0.01, 15.4) | 0.35 (0, 41.43) | **0 (0, 0.52)** | 0.45 (0, 50.03) | 3.01 (0.01, 1912.6) | 1.38 (0.03, 66.45) | 0.5 (0.01, 20.61) | **Erythromycin** |  |  |  |  |
| 0.12 (0, 82.18) | 0.22 (0, 66.25) | 0.14 (0, 35.34) | 0.12 (0, 46.46) | 0.09 (0, 31.9) | **0 (0, 0.19)** | 0.12 (0, 205.5) | 0.78 (0, 1162.95) | 0.36 (0, 110.16) | 0.13 (0, 55.81) | 0.26 (0, 89.35) | **Loracarbef** |  |  |  |
| 0 (0, 1.89) | 0 (0, 2.83) | 0 (0, 1.65) | 0 (0, 1.56) | 0 (0, 1.35) | **0 (0, 0)** | 0 (0, 2.46) | 0 (0, 19.36) | 0 (0, 4.62) | **0 (0, 0.85)** | 0 (0, 2.73) | 0 (0, 20.29) | **Miocamycin** |  |  |
| 0.42 (0, 40.82) | 0.74 (0.03, 18.37) | 0.49 (0.02, 8.81) | 0.42 (0.01, 15.27) | 0.31 (0.01, 9) | **0 (0, 0.3)** | 0.4 (0, 124.72) | 2.65 (0.01, 663.03) | 1.21 (0.06, 27.39) | 0.44 (0.01, 18.15) | 0.88 (0.03, 24.65) | 3.4 (0.03, 456.32) | 49406964197.44 (0.32, 3.22447882191701e+35) | **PenicillinV** |  |
| 0.06 (0, 15.52) | 0.11 (0, 11.23) | 0.07 (0, 5.61) | 0.06 (0, 7.8) | 0.04 (0, 5.64) | **0 (0, 0.06)** | 0.06 (0, 34.43) | 0.38 (0, 250.01) | 0.18 (0, 7.79) | 0.06 (0, 8.69) | 0.13 (0, 12.15) | 0.5 (0, 187.73) | 6245501118.89 (0.03, 4.88082618141213e+34) | 0.15 (0, 5) | **Telithromycin** |

Values are presented as odds ratios (95% confidence intervals). Penicillin V is used as reference. Statistically significant results are highlighted in bold. Extremely wide or undefined confidence intervals may occur when one treatment arm had 0% or 100% event rates. Abbreviations: AMC, amoxicillin-clavulanate.

**Supplementary Table 23. League table of pairwis**e comparisons for nausea (adverse event)

| **AMC** |  |  |  |  |  |  |  |  |  |  |  |  |  |  |
| --- | --- | --- | --- | --- | --- | --- | --- | --- | --- | --- | --- | --- | --- | --- |
| 0.3 (0, 18.8) | **Amoxicillin** |  |  |  |  |  |  |  |  |  |  |  |  |  |
| 0.75 (0.01, 26.93) | 2.38 (0.15, 47.36) | **Azithromycin** |  |  |  |  |  |  |  |  |  |  |  |  |
| 0.73 (0.01, 15.87) | 2.32 (0.15, 44.48) | 0.96 (0.07, 13.53) | **Cefaclor** |  |  |  |  |  |  |  |  |  |  |  |
| 6.26 (0.09, 442.08) | 19.79 (0.74, 1339.48) | 7.92 (0.8, 226.42) | 8.55 (0.5, 308.6) | **Cefdinir** |  |  |  |  |  |  |  |  |  |  |
| 9.77 (0.08, 854.61) | 31.52 (0.62, 2668.4) | 12.94 (0.6, 485.75) | 13.66 (0.4, 641.19) | 1.62 (0.06, 28.14) | **Cefotiam** |  |  |  |  |  |  |  |  |  |
| 0.34 (0, 10.25) | 1.08 (0.03, 38.98) | 0.44 (0.02, 10.44) | 0.48 (0.03, 4.95) | 0.06 (0, 1.19) | 0.03 (0, 1.36) | **Cefprozil** |  |  |  |  |  |  |  |  |
| 1.15 (0.01, 100.14) | 3.71 (0.08, 301.01) | 1.48 (0.07, 53.36) | 1.58 (0.05, 72.18) | 0.19 (0.01, 2.98) | 0.12 (0, 3.96) | 3.35 (0.09, 260.11) | **Cefuroxime Axetil** |  |  |  |  |  |  |  |
| 2.55 (0.04, 102.69) | 8.19 (0.27, 340.54) | 3.31 (0.28, 58.84) | 3.51 (0.2, 69.45) | 0.43 (0.02, 3.51) | 0.26 (0.01, 5.18) | 7.43 (0.37, 249.45) | 2.23 (0.09, 43.48) | **Clarithromycin** |  |  |  |  |  |  |
| 1.34 (0.02, 46.54) | 4.23 (0.15, 167.32) | 1.7 (0.14, 30.72) | 1.83 (0.12, 30.95) | 0.22 (0.01, 2.03) | 0.13 (0, 2.9) | 3.89 (0.23, 109.54) | 1.15 (0.04, 24.66) | 0.52 (0.05, 5.81) | **Dirithromycin** |  |  |  |  |  |
| 0.25 (0.01, 4.82) | 0.78 (0.03, 22.23) | 0.32 (0.02, 4.84) | 0.34 (0.03, 2.93) | **0.04 (0, 0.43)** | **0.02 (0, 0.58)** | 0.72 (0.08, 8.82) | 0.21 (0.01, 4.99) | **0.1 (0.01, 0.87)** | **0.19 (0.02, 1.29)** | **Erythromycin** |  |  |  |  |
| 12.03 (0.09, 1261.16) | 38.96 (0.72, 3853.34) | 15.59 (0.66, 744.91) | 16.52 (0.44, 944.22) | 1.93 (0.07, 43.37) | 1.21 (0.03, 53.21) | 36.4 (0.82, 3438.05) | 10.52 (0.28, 455.1) | 4.66 (0.22, 150.91) | 9.02 (0.38, 304.67) | 49.63 (1.86, 2186.58) | **Loracarbef** |  |  |  |
| 0.18 (0, 21.2) | 0.61 (0, 76.58) | 0.25 (0, 17.51) | 0.27 (0, 17.31) | 0.03 (0, 1.22) | 0.02 (0, 1.44) | 0.58 (0, 52.73) | 0.16 (0, 12.53) | 0.08 (0, 3.71) | 0.15 (0, 2.99) | 0.8 (0.01, 33.97) | 0.02 (0, 1.31) | **Miocamycin** |  |  |
| 4.39 (0.07, 168.28) | 13.86 (0.65, 495.34) | 5.58 (0.85, 68.26) | 6.01 (0.46, 102.17) | 0.72 (0.1, 2.97) | 0.44 (0.03, 5.34) | 12.73 (0.76, 409.53) | 3.74 (0.31, 45.92) | 1.68 (0.32, 11.33) | 3.22 (0.52, 25.38) | 17.7 (2.35, 203.14) | 0.37 (0.02, 4.98) | 22.3 (0.66, 1727.02) | **Penicillin V** |  |
| 0.72 (0.01, 35.28) | 2.29 (0.06, 114.23) | 0.93 (0.06, 19.29) | 0.99 (0.05, 24.6) | 0.12 (0.01, 1.09) | 0.07 (0, 1.57) | 2.1 (0.08, 89.79) | 0.63 (0.02, 13.11) | 0.28 (0.04, 1.78) | 0.55 (0.04, 7.27) | 2.9 (0.22, 48.36) | 0.06 (0, 1.4) | 3.72 (0.06, 382.94) | 0.17 (0.02, 1.03) | **Telithromycin** |

Values are presented as odds ratios (95% confidence intervals). Penicillin V is used as reference. Statistically significant results are highlighted in bold. Abbreviations: AMC, amoxicillin-clavulanate.

**Supplementary Table 24. League table of pairwise comparisons for abdominal pain (adverse event)**

| **Amoxicillin** |  |  |  |  |  |  |  |  |  |  |  |  |
| --- | --- | --- | --- | --- | --- | --- | --- | --- | --- | --- | --- | --- |
| 0.14 (0, 3.99) | **Azithromycin** |  |  |  |  |  |  |  |  |  |  |  |
| 0.79 (0.02, 13.97) | 5.62 (0.29, 226.51) | **Cefaclor** |  |  |  |  |  |  |  |  |  |  |
| 0.12 (0, 7.72) | 0.87 (0.03, 25.95) | 0.15 (0, 9.86) | **Cefdinir** |  |  |  |  |  |  |  |  |  |
| **0 (0, 0.02)** | **0 (0, 0.09)** | **0 (0, 0.02)** | **0 (0, 0.1)** | **Cefixime** |  |  |  |  |  |  |  |  |
| 0.27 (0, 36.84) | 1.97 (0.03, 155.91) | 0.35 (0, 47.99) | 2.26 (0.03, 221) | **20262983516226.1 (17.24, 3.02693096791948e+39)** | **Cefotiam** |  |  |  |  |  |  |  |
| 116350415.32 (0.47, 4.29331559716137e+28) | 946043408.94 (7.39, 3.34171472627771e+29) | 155114395.77 (0.86, 5.93589796014679e+28) | 1153116158.18 (6.93, 3.74896746094038e+29) | **3.53101789811898e+23 (727273.99, 3.36905004598154e+53)** | **502167587.33 (2.36, 1.89239681905027e+29)** | **Cefprozil** |  |  |  |  |  |  |
| 56295696.64 (0.1, 2.88208713457771e+25) | **498296257.62 (1.5, 1.90915887515163e+26)** | 79541853.04 (0.18, 3.44345499963771e+25) | **586149095.02 (1.57, 2.28453805679169e+26)** | **3.01290629722755e+23 (94106.89, 3.65810305894945e+51)** | 261467850.43 (0.55, 1.16313880290842e+26) | 0.27 (0, 79090874795326373898) | **Cefuroxime Axetil** |  |  |  |  |  |
| 0.94 (0, 168.68) | 6.59 (0.3, 640.98) | 1.18 (0.01, 218.52) | 7.41 (0.19, 1379.51) | **84289602647709.5 (72.46, 1.1647947780201e+40)** | 3.34 (0.04, 1223.68) | 0 (0, 1.25) | **0 (0, 8.3)** | **Clarithromycin** |  |  |  |  |
| 0.16 (0, 12.06) | 1.13 (0.05, 37.17) | 0.2 (0, 15.07) | 1.28 (0.03, 77.86) | **12056495715308.9 (12.24, 1.75894250950302e+39)** | 0.57 (0.01, 71.42) | **0 (0, 0.16)** | **0 (0, 0.93)** | 0.18 (0, 4.91) | **Dirithromycin** |  |  |  |
| 0.2 (0, 14.48) | 1.42 (0.1, 35.27) | 0.25 (0, 16.88) | 1.6 (0.04, 108.08) | **15206729467765.5 (15.16, 2.19348135656724e+39)** | 0.72 (0.01, 104.76) | **0 (0, 0.15)** | 0 (0, 1.2) | 0.22 (0.01, 3.03) | 1.24 (0.07, 24.85) | **Erythromycin** |  |  |
| 0.2 (0, 5.94) | 1.39 (0.16, 13.11) | 0.25 (0, 7) | 1.59 (0.12, 22.29) | **13481299529264.8 (18.36, 1.87903300508161e+39)** | 0.71 (0.02, 28.1) | **0 (0, 0.18)** | **0 (0, 0.79)** | 0.22 (0, 3.53) | 1.25 (0.06, 17.99) | 1 (0.04, 14.63) | **PenicillinV** |  |
| 1.48 (0, 372.09) | 10.74 (0.12, 1586.01) | 1.92 (0.01, 480.87) | 12.42 (0.11, 2219.53) | **117826458015709 (85.76, 1.96397798223607e+40)** | 5.5 (0.02, 1780.36) | 0 (0, 2.84) | 0 (0, 13.02) | 1.58 (0, 288.98) | 9.37 (0.06, 1719.64) | 7.56 (0.04, 1334.99) | 7.55 (0.15, 698.67) | **Telithromycin** |

Values are presented as odds ratios (95% confidence intervals). Penicillin V is used as reference. Statistically significant results are highlighted in bold. Extremely wide or undefined confidence intervals may occur when one treatment arm had 0% or 100% event rates.

**Supplementary Table 25**. League table of pairwise comparisons for rash (adverse event)

| **AMC** |  |  |  |  |  |  |  |  |  |  |  |  |  |  |  |
| --- | --- | --- | --- | --- | --- | --- | --- | --- | --- | --- | --- | --- | --- | --- | --- |
| 28.71 (0.57, 3247.71) | **Amoxicillin** |  |  |  |  |  |  |  |  |  |  |  |  |  |  |
| **163.09 (2.4, 125055.4)** | 5.19 (0.4, 600.79) | **Azithromycin** |  |  |  |  |  |  |  |  |  |  |  |  |  |
| 11.17 (0.04, 9519.98) | 0.37 (0, 54) | 0.07 (0, 4.12) | **BPV** |  |  |  |  |  |  |  |  |  |  |  |  |
| 5.4 (0.16, 373.03) | 0.19 (0.02, 1.55) | **0.04 (0, 0.57)** | 0.53 (0, 67.25) | **Cefaclor** |  |  |  |  |  |  |  |  |  |  |  |
| **594749809432536 (366.16, 1.0064159354313e+41)** | **19618739173578.6 (24.12, 3.01387019380552e+39)** | **2768135347964.03 (2.99, 6.5462454699746e+38)** | **54538834460069.8 (30.22, 1.26456877100826e+40)** | **99200970783779.5 (114.84, 1.66275499395764e+40)** | **Cefcapene Pivoxil** |  |  |  |  |  |  |  |  |  |  |
| 22.72 (0.25, 13120.66) | 0.73 (0.04, 57.84) | 0.14 (0, 3.72) | 2.04 (0.03, 304.75) | 3.86 (0.15, 536.16) | **0 (0, 0.05)** | **Cefdinir** |  |  |  |  |  |  |  |  |  |
| 0 (0, 1.57) | **0 (0, 0.02)** | **0 (0, 0)** | **0 (0, 0.08)** | **0 (0, 0.13)** | **0 (0, 0)** | **0 (0, 0.03)** | **Cefprozil** |  |  |  |  |  |  |  |  |
| 45.52 (0.49, 25762.55) | 1.47 (0.08, 112.84) | 0.27 (0, 7.87) | 4.13 (0.06, 623.09) | 7.75 (0.28, 1065.21) | **0 (0, 0.09)** | 1.99 (0.06, 60.46) | **589100040303827 (70.09, 3.49327783421854e+38)** | **Cefuroxime Axetil** |  |  |  |  |  |  |  |
| 2.41 (0.01, 1623.99) | 0.08 (0, 7.36) | **0.02 (0, 0.58)** | 0.23 (0, 39.27) | 0.44 (0.01, 66.51) | **0 (0, 0.01)** | 0.11 (0, 4.28) | **27167010688126.1 (4.29, 1.59612049211218e+37)** | 0.05 (0, 2.27) | **Clarithromycin** |  |  |  |  |  |  |
| 41.97 (0.19, 79447.9) | 1.38 (0.01, 688.17) | 0.25 (0, 61.79) | 3.93 (0.01, 3158.91) | 7.44 (0.06, 5056.64) | **0 (0, 0.14)** | 1.88 (0.01, 503.36) | **569958467658391 (53.59, 3.395721601768e+38)** | 0.92 (0, 264.37) | 17.7 (0.06, 10548.54) | **Dirithromycin** |  |  |  |  |  |
| **93.48 (1.32, 78059.48)** | 3.16 (0.1, 565.64) | 0.57 (0.01, 56.71) | 9.01 (0.07, 3046.65) | 16.65 (0.47, 4303.19) | **0 (0, 0.24)** | 4.15 (0.07, 433.64) | **1310427995292878 (177.35, 7.7802919130775e+38)** | 2.1 (0.03, 236.85) | 39.44 (0.46, 10102.04) | 2.28 (0.09, 73.18) | **Erythromycin** |  |  |  |  |
| 44.96 (0.41, 23805.03) | 1.47 (0.06, 100.17) | 0.27 (0, 7.62) | 4.06 (0.05, 568.67) | 7.65 (0.23, 943.91) | **0 (0, 0.08)** | 1.96 (0.05, 55.46) | **540798708239274 (88.31, 2.81806949236729e+38)** | 1 (0.02, 30.61) | 17.53 (0.35, 1782.51) | 1.08 (0, 234.17) | 0.47 (0, 29.28) | **Loracarbef** |  |  |  |
| 24.58 (0.46, 7514.88) | 0.82 (0.1, 21.37) | 0.16 (0.01, 1.53) | 2.2 (0.06, 166.25) | 4.23 (0.32, 237.31) | **0 (0, 0.04)** | 1.11 (0.09, 10.8) | **313954931795859 (41.91, 1.58745304420751e+38)** | 0.56 (0.04, 5.74) | 9.55 (0.55, 474.49) | 0.6 (0, 70.53) | 0.27 (0, 6.97) | 0.56 (0.04, 7.52) | **Penicillin V** |  |  |
| 34.49 (0.25, 27128.19) | 1.11 (0.03, 141.74) | 0.22 (0, 9.67) | 3.19 (0.03, 665.43) | 5.86 (0.13, 1269.05) | **0 (0, 0.07)** | 1.53 (0.03, 74.54) | **470033463912696 (44.54, 2.57933626424499e+38)** | 0.77 (0.01, 41.66) | 13.59 (0.2, 2070.01) | 0.82 (0, 252.03) | 0.36 (0, 35.84) | 0.78 (0.01, 50.88) | 1.39 (0.06, 35.47) | **Spiramycin** |  |
| 32.42 (0.25, 24401.81) | 1.02 (0.03, 122.83) | 0.2 (0, 7.97) | 2.94 (0.03, 584.82) | 5.41 (0.12, 1111.06) | **0 (0, 0.07)** | 1.41 (0.03, 65.47) | **423193040483736 (45.65, 2.43817639744695e+38)** | 0.7 (0.01, 34.23) | 12.8 (0.19, 1785.68) | 0.77 (0, 227.17) | 0.34 (0, 29.26) | 0.71 (0.01, 43.07) | 1.28 (0.06, 29.3) | 0.92 (0.01, 79.08) | **Telithromycin** |

Values are presented as odds ratios (95% confidence intervals). Penicillin V is used as reference. Statistically significant results are highlighted in bold. Extremely wide or undefined confidence intervals may occur when one treatment arm had 0% or 100% event rates. Abbreviations: AMC, amoxicillin-clavulanate; BPV, Benzathine Penicillin V.

**Supplementary Table 26. League table of pairwise comparisons for headache (adverse event)**

| **Azithromycin** |  |  |  |  |  |  |  |  |  |
| --- | --- | --- | --- | --- | --- | --- | --- | --- | --- |
| 0 (0, 1.47) | **Cefaclor** |  |  |  |  |  |  |  |  |
| **670969674.03 (2.47, 3.81045432404731e+27)** | **1709813370588594944 (2263.96, 2.2735392670293e+42)** | **Cefdinir** |  |  |  |  |  |  |  |
| 147572257.74 (0.32, 9.72822459006591e+26) | **392796891857362112 (411.35, 6.47851562847194e+41)** | 0.23 (0, 37.76) | **Cefotiam** |  |  |  |  |  |  |
| **1394964428.43 (4.59, 9.02525774500769e+27)** | **3611519334644472832 (4304.53, 5.0626964719796e+42)** | 1.92 (0.04, 161.87) | 8.58 (0.05, 3147.52) | **Clarithromycin** |  |  |  |  |  |
| **859367456.73 (3.88, 4.44760105115949e+27)** | **2222667258626709248 (3246.71, 2.99248899506927e+42)** | 1.3 (0.01, 123.65) | 5.65 (0.02, 2376.09) | 0.68 (0, 65.62) | **Dirithromycin** |  |  |  |  |
| **1234784093.71 (5.5, 6.75410891165393e+27)** | **3166481878893506560 (4272.78, 4.34590859646627e+42)** | 1.78 (0, 748.94) | 7.91 (0.01, 10743.01) | 0.93 (0, 368.26) | 1.38 (0.03, 72.31) | **Erythromycin** |  |  |  |
| 259323625.46 (0.99, 1.69751087378664e+27) | **689211424416199808 (887.82, 9.14882726173131e+41)** | 0.4 (0.01, 18.87) | 1.75 (0.01, 433.52) | 0.21 (0, 10.77) | 0.31 (0, 30.32) | 0.22 (0, 88.46) | **Loracarbef** |  |  |
| **1.7754818342518e+23 (122291.33, 9.16680053852562e+50)** | **5.01178072808189e+32 (1606342068.45, 5.65495852451632e+63)** | **2699495103022.18 (3.94, 6.64678301667665e+38)** | **12687840347370.9 (11.52, 2.96734386605223e+39)** | **1268158082812.88 (1.85, 3.12722240189877e+38)** | **1977674873305.28 (5.29, 4.71429131252319e+38)** | **1456585965620.1 (2.4, 3.45408920847651e+38)** | **6630992489570.89 (9.68, 1.64578109723286e+39)** | **Miocamycin** |  |
| **650052643 (3.19, 3.85358983337054e+27)** | **1697482228190929408 (2365.21, 2.13693325335615e+42)** | 0.96 (0.06, 14.79) | 4.15 (0.06, 513.58) | 0.5 (0.02, 9.14) | 0.75 (0.02, 29.56) | 0.54 (0, 110.39) | 2.39 (0.15, 39.81) | **0 (0, 0.2)** | **PenicillinV** |

Values are presented as odds ratios (95% confidence intervals). Penicillin V is used as reference. Statistically significant results are highlighted in bold. Extremely wide or undefined confidence intervals may occur when one treatment arm had 0% or 100% event rates.

**Supplementary Table 27. League table of pairwise comparisons for early bacterial eradication**

| **AMC** |  |  |  |  |  |  |  |  |  |  |  |  |  |  |  |  |  |  |  |  |  |
| --- | --- | --- | --- | --- | --- | --- | --- | --- | --- | --- | --- | --- | --- | --- | --- | --- | --- | --- | --- | --- | --- |
| 1.02 (0.43, 2.39) | **Amoxicillin** |  |  |  |  |  |  |  |  |  |  |  |  |  |  |  |  |  |  |  |  |
| 1.82 (0.73, 4.44) | 1.79 (0.87, 3.61) | **Azithromycin** |  |  |  |  |  |  |  |  |  |  |  |  |  |  |  |  |  |  |  |
| 0.74 (0.1, 5.82) | 0.73 (0.1, 5.31) | 0.41 (0.06, 2.93) | **BPG** |  |  |  |  |  |  |  |  |  |  |  |  |  |  |  |  |  |  |
| 1.14 (0.21, 6.28) | 1.12 (0.23, 5.62) | 0.63 (0.14, 3.08) | 1.54 (0.14, 16.74) | **BPV** |  |  |  |  |  |  |  |  |  |  |  |  |  |  |  |  |  |
| 0.97 (0.4, 2.3) | 0.96 (0.43, 2.11) | 0.53 (0.24, 1.2) | 1.31 (0.17, 9.78) | 0.85 (0.16, 4.44) | **Cefaclor** |  |  |  |  |  |  |  |  |  |  |  |  |  |  |  |  |
| 0.73 (0.1, 5.07) | 0.72 (0.12, 4.06) | 0.4 (0.06, 2.67) | 0.99 (0.07, 13.46) | 0.64 (0.06, 6.64) | 0.75 (0.11, 5.1) | **Cefcapene Pivoxil** |  |  |  |  |  |  |  |  |  |  |  |  |  |  |  |
| **0.31 (0.11, 0.9)** | **0.31 (0.13, 0.74)** | **0.17 (0.08, 0.4)** | 0.42 (0.06, 3.05) | 0.28 (0.05, 1.36) | **0.32 (0.12, 0.9)** | 0.43 (0.06, 3.13) | **Cefdinir** |  |  |  |  |  |  |  |  |  |  |  |  |  |  |
| 0.58 (0.07, 4.35) | 0.57 (0.08, 3.93) | 0.32 (0.04, 2.16) | 0.78 (0.05, 10.73) | 0.51 (0.05, 5.35) | 0.6 (0.08, 4.45) | 0.79 (0.06, 11.04) | 1.86 (0.25, 13.2) | **Cefetamet Pivoxil** |  |  |  |  |  |  |  |  |  |  |  |  |  |
| 0.63 (0.15, 2.53) | 0.62 (0.17, 2.2) | 0.35 (0.1, 1.2) | 0.85 (0.09, 7.5) | 0.56 (0.08, 3.48) | 0.65 (0.16, 2.58) | 0.86 (0.1, 7.74) | 2.02 (0.53, 7.42) | 1.09 (0.12, 9.78) | **Cefixime** |  |  |  |  |  |  |  |  |  |  |  |  |
| 0.37 (0.11, 1.25) | 0.37 (0.13, 1.07) | **0.21 (0.08, 0.58)** | 0.5 (0.06, 3.96) | 0.33 (0.06, 1.8) | 0.39 (0.12, 1.26) | 0.51 (0.07, 4.09) | 1.2 (0.4, 3.61) | 0.65 (0.08, 5.25) | 0.59 (0.14, 2.57) | **Cefpodoxime Proxetil** |  |  |  |  |  |  |  |  |  |  |  |
| 0.2 (0.03, 1.29) | 0.2 (0.03, 1.24) | **0.11 (0.02, 0.68)** | 0.27 (0.02, 3.53) | 0.17 (0.02, 1.76) | 0.21 (0.03, 1.33) | 0.27 (0.02, 3.52) | 0.64 (0.08, 4.27) | 0.34 (0.02, 4.64) | 0.31 (0.03, 2.69) | 0.53 (0.06, 3.85) | **Cefprozil** |  |  |  |  |  |  |  |  |  |  |
| 0.64 (0.21, 1.98) | 0.63 (0.25, 1.67) | **0.35 (0.14, 0.9)** | 0.87 (0.15, 4.77) | 0.57 (0.11, 2.94) | 0.66 (0.23, 2) | 0.88 (0.12, 6.71) | 2.06 (0.76, 5.66) | 1.11 (0.15, 8.59) | 1.02 (0.26, 4.09) | 1.72 (0.54, 5.44) | 3.24 (0.47, 25.3) | **Cefuroxime Axetil** |  |  |  |  |  |  |  |  |  |
| 0.72 (0.27, 1.92) | 0.71 (0.32, 1.6) | **0.4 (0.2, 0.8)** | 0.97 (0.13, 6.82) | 0.63 (0.13, 3.04) | 0.74 (0.29, 1.9) | 0.98 (0.14, 7.03) | 2.29 (0.95, 5.6) | 1.24 (0.18, 9.14) | 1.14 (0.32, 4.24) | 1.91 (0.67, 5.52) | 3.61 (0.58, 25.7) | 1.12 (0.43, 2.9) | **Clarithromycin** |  |  |  |  |  |  |  |  |
| 0.34 (0.07, 1.62) | 0.33 (0.05, 1.99) | 0.19 (0.03, 1.15) | 0.46 (0.03, 5.99) | 0.3 (0.03, 2.96) | 0.35 (0.05, 2.11) | 0.46 (0.04, 5.84) | 1.09 (0.16, 7.26) | 0.58 (0.04, 7.88) | 0.53 (0.06, 4.48) | 0.91 (0.12, 6.48) | 1.72 (0.14, 21.5) | 0.53 (0.07, 3.62) | 0.47 (0.07, 3.01) | **Clindamycin** |  |  |  |  |  |  |  |
| 1.57 (0.42, 5.77) | 1.55 (0.46, 5.19) | 0.86 (0.27, 2.83) | 2.12 (0.24, 18.11) | 1.38 (0.22, 8.57) | 1.62 (0.45, 5.84) | 2.16 (0.26, 18.42) | **5.02 (1.4, 18.03)** | 2.71 (0.31, 24.26) | 2.48 (0.52, 12.36) | **4.19 (1.02, 16.87)** | **7.89 (1.13, 60.64)** | 2.44 (0.64, 9.09) | 2.19 (0.64, 7.26) | 4.65 (0.6, 37.21) | **Dirithromycin** |  |  |  |  |  |  |
| 1.57 (0.58, 4.21) | 1.55 (0.61, 3.88) | 0.87 (0.37, 2.08) | 2.13 (0.27, 15.98) | 1.39 (0.25, 7.22) | 1.62 (0.62, 4.25) | 2.16 (0.3, 16.01) | **5.04 (1.78, 14.02)** | 2.72 (0.36, 21.23) | 2.5 (0.62, 10.15) | **4.2 (1.28, 13.54)** | **7.91 (1.59, 44.96)** | 2.45 (0.81, 7.21) | 2.2 (0.88, 5.38) | 4.64 (0.72, 31.39) | 1 (0.34, 2.97) | **Erythromycin** |  |  |  |  |  |
| 0.64 (0.19, 2.14) | 0.63 (0.22, 1.82) | **0.35 (0.13, 0.99)** | 0.87 (0.11, 6.77) | 0.57 (0.1, 3.09) | 0.66 (0.21, 2.16) | 0.88 (0.12, 6.96) | 2.06 (0.69, 6.17) | 1.11 (0.14, 8.95) | 1.02 (0.25, 4.39) | 1.72 (0.5, 5.86) | 3.24 (0.45, 26.35) | 1 (0.31, 3.16) | 0.9 (0.31, 2.56) | 1.9 (0.26, 14.37) | 0.41 (0.1, 1.67) | 0.41 (0.13, 1.33) | **Loracarbef** |  |  |  |  |
| 4.29 (0.18, 195.28) | 4.21 (0.18, 186.06) | 2.34 (0.1, 104.5) | 5.86 (0.15, 370.59) | 3.8 (0.12, 206.83) | 4.41 (0.19, 202.35) | 5.94 (0.16, 377.61) | 13.68 (0.58, 623.31) | 7.57 (0.2, 472.04) | 6.8 (0.25, 352.34) | 11.41 (0.46, 532.18) | 21.92 (0.66, 1321.61) | 6.63 (0.27, 309.22) | 5.95 (0.26, 265.15) | 12.86 (0.36, 768.79) | 2.69 (0.15, 102.68) | 2.71 (0.12, 118.26) | 6.65 (0.27, 316.01) | **Miocamycin** |  |  |  |
| 0.97 (0.42, 2.2) | 0.95 (0.53, 1.71) | **0.53 (0.32, 0.9)** | 1.3 (0.2, 8.41) | 0.85 (0.19, 3.63) | 1 (0.46, 2.2) | 1.32 (0.21, 8.63) | **3.09 (1.6, 6.01)** | 1.66 (0.26, 11.24) | 1.53 (0.5, 4.92) | **2.58 (1.07, 6.17)** | 4.85 (0.82, 32.77) | 1.5 (0.7, 3.17) | 1.35 (0.74, 2.42) | 2.84 (0.49, 17.82) | 0.62 (0.21, 1.86) | 0.61 (0.28, 1.37) | 1.5 (0.63, 3.6) | 0.23 (0.01, 4.96) | **Penicillin V** |  |  |
| 2.26 (0.43, 11.89) | 2.23 (0.48, 10.6) | 1.24 (0.27, 5.83) | 3.06 (0.29, 31.98) | 1.99 (0.25, 15.39) | 2.33 (0.46, 12.22) | 3.11 (0.3, 33.16) | **7.23 (1.48, 35.54)** | 3.9 (0.38, 42.27) | 3.58 (0.58, 22.89) | **6.04 (1.11, 32.33)** | **11.43 (1.15, 122.61)** | 3.52 (0.69, 17.78) | 3.16 (0.66, 14.91) | 6.69 (0.69, 67.71) | 1.44 (0.24, 8.83) | 1.43 (0.28, 7.53) | 3.51 (0.65, 18.9) | 0.52 (0.01, 16.03) | 2.34 (0.56, 9.96) | **Spiramycin** |  |
| 0.83 (0.22, 3.15) | 0.82 (0.25, 2.7) | 0.46 (0.15, 1.46) | 1.12 (0.13, 9.46) | 0.73 (0.12, 4.44) | 0.86 (0.24, 3.17) | 1.14 (0.14, 9.82) | 2.66 (0.77, 9.23) | 1.44 (0.17, 12.65) | 1.32 (0.29, 6.35) | 2.22 (0.57, 8.74) | 4.21 (0.53, 36.38) | 1.3 (0.35, 4.7) | 1.16 (0.4, 3.32) | 2.45 (0.32, 20.19) | 0.53 (0.12, 2.42) | 0.53 (0.15, 1.92) | 1.29 (0.33, 5.07) | 0.19 (0, 5.11) | 0.86 (0.3, 2.47) | 0.37 (0.06, 2.19) | **Telithromycin** |

Values are presented as odds ratios (95% confidence intervals). Penicillin V is used as reference. Statistically significant results are highlighted in bold. Abbreviations: AMC, amoxicillin/clavulanate; BPG, Benzathine Penicillin G; BPV, Benzathine Penicillin V.

**Supplementary Table 28. League table of pairwise comparisons for late bacterial eradication**

| **Amoxicillin** |  |  |  |  |  |  |  |  |  |  |  |  |  |  |  |  |  |  |  |
| --- | --- | --- | --- | --- | --- | --- | --- | --- | --- | --- | --- | --- | --- | --- | --- | --- | --- | --- | --- |
| 0.66 (0.3, 1.4) | **AMC** |  |  |  |  |  |  |  |  |  |  |  |  |  |  |  |  |  |  |
| **2.72 (1.48, 4.75)** | **4.13 (1.86, 8.83)** | **Azithromycin** |  |  |  |  |  |  |  |  |  |  |  |  |  |  |  |  |  |
| 1.15 (0.53, 2.51) | 1.75 (0.6, 5.18) | 0.42 (0.17, 1.14) | **BPG** |  |  |  |  |  |  |  |  |  |  |  |  |  |  |  |  |
| 0.96 (0.5, 1.79) | 1.45 (0.7, 3) | **0.35 (0.18, 0.7)** | 0.83 (0.3, 2.22) | **Cefaclor** |  |  |  |  |  |  |  |  |  |  |  |  |  |  |  |
| 0.81 (0.33, 1.91) | 1.23 (0.43, 3.4) | **0.3 (0.12, 0.74)** | 0.7 (0.21, 2.21) | 0.85 (0.32, 2.29) | **Cefdinir** |  |  |  |  |  |  |  |  |  |  |  |  |  |  |
| 0.75 (0.14, 4.28) | 1.15 (0.19, 7.03) | 0.28 (0.05, 1.6) | 0.66 (0.1, 4.33) | 0.78 (0.13, 4.83) | 0.93 (0.15, 5.94) | **Cefixime** |  |  |  |  |  |  |  |  |  |  |  |  |  |
| 1.17 (0.36, 3.78) | 1.77 (0.49, 6.46) | 0.43 (0.13, 1.44) | 1.01 (0.25, 4.11) | 1.22 (0.35, 4.39) | 1.43 (0.38, 5.58) | 1.54 (0.21, 11.36) | **Cefotiam** |  |  |  |  |  |  |  |  |  |  |  |  |
| 0.72 (0.35, 1.53) | 1.09 (0.45, 2.81) | **0.26 (0.13, 0.59)** | 0.63 (0.22, 1.84) | 0.75 (0.33, 1.88) | 0.89 (0.35, 2.44) | 0.96 (0.16, 5.63) | 0.62 (0.18, 2.21) | **Cefpodoxime Proxetil** |  |  |  |  |  |  |  |  |  |  |  |
| 0.33 (0.09, 1.08) | 0.5 (0.12, 1.79) | **0.12 (0.03, 0.4)** | 0.29 (0.06, 1.16) | 0.35 (0.09, 1.16) | 0.41 (0.09, 1.61) | 0.43 (0.05, 3.24) | 0.28 (0.05, 1.36) | 0.46 (0.11, 1.6) | **Cefprozil** |  |  |  |  |  |  |  |  |  |  |
| 0.61 (0.27, 1.25) | 0.92 (0.35, 2.29) | **0.22 (0.1, 0.48)** | 0.53 (0.17, 1.5) | 0.64 (0.25, 1.53) | 0.75 (0.27, 1.99) | 0.81 (0.13, 4.64) | 0.52 (0.14, 1.79) | 0.85 (0.33, 1.91) | 1.84 (0.49, 7.49) | **Cefuroxime Axetil** |  |  |  |  |  |  |  |  |  |
| 0.63 (0.22, 1.8) | 0.96 (0.29, 3.14) | **0.23 (0.08, 0.69)** | 0.55 (0.15, 1.99) | 0.66 (0.21, 2.13) | 0.78 (0.23, 2.68) | 0.85 (0.12, 5.59) | 0.54 (0.13, 2.31) | 0.88 (0.27, 2.65) | 1.92 (0.44, 9.33) | 1.04 (0.34, 3.43) | **Cephalexin** |  |  |  |  |  |  |  |  |
| 1.51 (0.84, 2.65) | **2.29 (1.11, 4.68)** | **0.56 (0.33, 0.97)** | 1.31 (0.49, 3.37) | 1.58 (0.79, 3.19) | 1.86 (0.79, 4.48) | 2.01 (0.35, 10.98) | 1.3 (0.4, 4.13) | 2.1 (0.97, 4.22) | **4.54 (1.46, 16.52)** | **2.48 (1.19, 5.48)** | 2.39 (0.82, 6.74) | **Clarithromycin** |  |  |  |  |  |  |  |
| 1.57 (0.1, 51.07) | 2.37 (0.17, 71.51) | 0.58 (0.04, 18.88) | 1.37 (0.08, 47.52) | 1.64 (0.11, 52.51) | 1.98 (0.12, 65.69) | 2.11 (0.08, 95.32) | 1.37 (0.07, 48.9) | 2.17 (0.13, 71.73) | 4.93 (0.26, 182.49) | 2.63 (0.16, 87.15) | 2.5 (0.14, 90.08) | 1.04 (0.07, 33.23) | **Clindamycin** |  |  |  |  |  |  |
| 0.95 (0.36, 2.55) | 1.44 (0.5, 4.25) | **0.35 (0.13, 0.97)** | 0.83 (0.24, 2.87) | 0.99 (0.36, 2.87) | 1.17 (0.37, 3.95) | 1.26 (0.19, 8.31) | 0.81 (0.2, 3.36) | 1.31 (0.44, 3.85) | 2.88 (0.69, 13.57) | 1.56 (0.54, 4.95) | 1.49 (0.41, 5.75) | 0.63 (0.24, 1.73) | 0.61 (0.02, 10.44) | **Dirithromycin** |  |  |  |  |  |
| 0.78 (0.25, 2.48) | 1.18 (0.37, 3.84) | **0.29 (0.09, 0.93)** | 0.68 (0.17, 2.66) | 0.82 (0.26, 2.59) | 0.96 (0.25, 3.86) | 1.04 (0.14, 7.46) | 0.67 (0.14, 3.16) | 1.08 (0.3, 3.76) | 2.38 (0.5, 12.19) | 1.28 (0.37, 4.8) | 1.22 (0.29, 5.38) | 0.51 (0.16, 1.67) | 0.49 (0.01, 8.88) | 0.82 (0.3, 2.2) | **Erythromycin** |  |  |  |  |
| 0.95 (0.43, 2.08) | 1.44 (0.56, 3.79) | **0.35 (0.16, 0.8)** | 0.82 (0.27, 2.47) | 0.99 (0.4, 2.52) | 1.17 (0.43, 3.27) | 1.26 (0.21, 7.56) | 0.82 (0.23, 2.95) | 1.31 (0.53, 3.16) | 2.88 (0.77, 12.13) | 1.56 (0.65, 4.12) | 1.5 (0.47, 4.89) | 0.63 (0.29, 1.4) | 0.6 (0.02, 9.98) | 0.99 (0.32, 3.03) | 1.22 (0.33, 4.42) | **Loracarbef** |  |  |  |
| 1.04 (0.68, 1.58) | 1.57 (0.8, 3.18) | **0.38 (0.25, 0.62)** | 0.9 (0.37, 2.16) | 1.08 (0.59, 2.08) | 1.28 (0.61, 2.8) | 1.38 (0.26, 7.22) | 0.89 (0.3, 2.67) | 1.44 (0.78, 2.57) | **3.12 (1.03, 11.25)** | 1.7 (0.94, 3.41) | 1.63 (0.63, 4.34) | 0.69 (0.46, 1.06) | 0.66 (0.02, 10.05) | 1.1 (0.44, 2.66) | 1.34 (0.44, 4.05) | 1.09 (0.56, 2.14) | **Penicillin V** |  |  |
| 0.74 (0.25, 2.19) | 1.11 (0.33, 3.82) | **0.27 (0.09, 0.84)** | 0.64 (0.17, 2.41) | 0.76 (0.24, 2.59) | 0.9 (0.26, 3.3) | 0.97 (0.14, 6.77) | 0.63 (0.14, 2.79) | 1.02 (0.31, 3.23) | 2.22 (0.49, 11.3) | 1.2 (0.38, 4.19) | 1.16 (0.29, 4.74) | 0.48 (0.16, 1.48) | 0.46 (0.01, 8.58) | 0.77 (0.2, 2.95) | 0.95 (0.21, 4.2) | 0.77 (0.23, 2.61) | 0.71 (0.26, 1.94) | **Spiramycin** |  |
| 1.28 (0.53, 2.96) | 1.93 (0.71, 5.16) | 0.47 (0.2, 1.11) | 1.1 (0.34, 3.44) | 1.33 (0.51, 3.49) | 1.57 (0.54, 4.6) | 1.68 (0.26, 10.33) | 1.1 (0.29, 4.09) | 1.77 (0.65, 4.48) | **3.84 (1.02, 16.24)** | 2.09 (0.8, 5.8) | 2.01 (0.58, 6.82) | 0.84 (0.4, 1.78) | 0.81 (0.02, 13.3) | 1.34 (0.41, 4.23) | 1.63 (0.43, 6.13) | 1.34 (0.48, 3.6) | 1.23 (0.57, 2.57) | 1.74 (0.49, 6.03) | **Telithromycin** |

Values are presented as odds ratios (95% confidence intervals). Penicillin V is used as reference. Statistically significant results are highlighted in bold. Abbreviations: AMC, amoxicillin/clavulanate; BPG, Benzathine Penicillin G.

**Supplementary Table 29. League table of pairwise comparisons for early clinical response**

| **Amoxicillin** |  |  |  |  |  |  |  |  |  |  |  |  |  |  |  |  |  |  |  |  |
| --- | --- | --- | --- | --- | --- | --- | --- | --- | --- | --- | --- | --- | --- | --- | --- | --- | --- | --- | --- | --- |
| 0.41 (0.13, 1.16) | **AMC** |  |  |  |  |  |  |  |  |  |  |  |  |  |  |  |  |  |  |  |
| 0.64 (0.34, 1.23) | 1.58 (0.55, 5.02) | **Azithromycin** |  |  |  |  |  |  |  |  |  |  |  |  |  |  |  |  |  |  |
| 0.32 (0.03, 3.81) | 0.8 (0.06, 11.06) | 0.49 (0.04, 5.92) | **BPG** |  |  |  |  |  |  |  |  |  |  |  |  |  |  |  |  |  |
| 1.88 (0.2, 54.03) | 4.65 (0.41, 148.94) | 2.92 (0.32, 82.15) | 6.33 (0.21, 319.03) | **BPV** |  |  |  |  |  |  |  |  |  |  |  |  |  |  |  |  |
| 0.7 (0.32, 1.5) | 1.71 (0.54, 5.66) | 1.08 (0.47, 2.42) | 2.18 (0.17, 26.86) | 0.37 (0.01, 3.75) | **Cefaclor** |  |  |  |  |  |  |  |  |  |  |  |  |  |  |  |
| 1.21 (0.16, 11.45) | 3.03 (0.32, 33.5) | 1.88 (0.26, 16.25) | 3.85 (0.17, 97.34) | 0.62 (0.02, 13.88) | 1.74 (0.2, 17.75) | **Cefcapene Pivoxil** |  |  |  |  |  |  |  |  |  |  |  |  |  |  |
| **0.33 (0.16, 0.65)** | 0.81 (0.27, 2.6) | **0.52 (0.26, 0.99)** | 1.05 (0.08, 11.68) | 0.18 (0.01, 1.6) | 0.48 (0.19, 1.2) | 0.28 (0.03, 2.16) | **Cefdinir** |  |  |  |  |  |  |  |  |  |  |  |  |  |
| **0.26 (0.07, 0.84)** | 0.63 (0.13, 2.92) | 0.4 (0.11, 1.31) | 0.79 (0.05, 11.55) | 0.13 (0, 1.56) | 0.37 (0.09, 1.43) | 0.21 (0.02, 2.13) | 0.77 (0.22, 2.56) | **Cefixime** |  |  |  |  |  |  |  |  |  |  |  |  |
| **0.42 (0.18, 0.97)** | 1.03 (0.31, 3.6) | 0.65 (0.29, 1.47) | 1.31 (0.1, 15.62) | 0.22 (0.01, 2.13) | 0.6 (0.22, 1.7) | 0.34 (0.04, 2.88) | 1.26 (0.57, 2.87) | 1.62 (0.46, 6.37) | **Cefpodoxime Proxetil** |  |  |  |  |  |  |  |  |  |  |  |
| 0.64 (0.19, 2.3) | 1.6 (0.36, 7.41) | 1 (0.29, 3.57) | 2.04 (0.13, 30.67) | 0.34 (0.01, 4.12) | 0.92 (0.28, 3.25) | 0.53 (0.04, 5.46) | 1.92 (0.55, 7.34) | 2.52 (0.5, 13.62) | 1.54 (0.4, 6.19) | **Cefprozil** |  |  |  |  |  |  |  |  |  |  |
| **0.31 (0.15, 0.68)** | 0.77 (0.25, 2.62) | 0.49 (0.24, 1.03) | 1 (0.09, 10.22) | 0.17 (0.01, 1.58) | 0.45 (0.18, 1.22) | 0.26 (0.03, 2.12) | 0.94 (0.48, 2.04) | 1.23 (0.37, 4.5) | 0.75 (0.32, 1.82) | 0.49 (0.13, 1.82) | **Cefuroxime Axetil** |  |  |  |  |  |  |  |  |  |
| 0.49 (0.24, 1.02) | 1.21 (0.42, 3.75) | 0.77 (0.38, 1.55) | 1.58 (0.13, 17.75) | 0.26 (0.01, 2.39) | 0.71 (0.29, 1.83) | 0.41 (0.04, 3.35) | 1.49 (0.75, 3.05) | 1.92 (0.57, 7.14) | 1.19 (0.51, 2.75) | 0.77 (0.22, 2.66) | 1.58 (0.72, 3.31) | **Clarithromycin** |  |  |  |  |  |  |  |  |
| **0.18 (0.05, 0.7)** | 0.45 (0.19, 1.05) | 0.28 (0.07, 1.09) | 0.56 (0.04, 8.74) | 0.1 (0, 1.26) | 0.26 (0.06, 1.1) | 0.15 (0.01, 1.64) | 0.55 (0.13, 2.2) | 0.71 (0.12, 4.13) | 0.44 (0.1, 1.87) | 0.28 (0.05, 1.54) | 0.58 (0.13, 2.33) | 0.37 (0.09, 1.42) | **Clindamycin** |  |  |  |  |  |  |  |
| 0.54 (0.17, 1.69) | 1.34 (0.33, 5.75) | 0.84 (0.28, 2.53) | 1.71 (0.12, 22.86) | 0.28 (0.01, 3.19) | 0.78 (0.22, 2.71) | 0.45 (0.04, 4.21) | 1.63 (0.53, 5.05) | 2.11 (0.47, 9.96) | 1.3 (0.37, 4.35) | 0.84 (0.21, 3.34) | 1.73 (0.52, 5.33) | 1.09 (0.36, 3.27) | 2.95 (0.58, 16.12) | **Dirithromycin** |  |  |  |  |  |  |
| 0.55 (0.21, 1.46) | 1.37 (0.37, 5.09) | 0.86 (0.33, 2.19) | 1.73 (0.12, 22.3) | 0.29 (0.01, 2.95) | 0.79 (0.27, 2.34) | 0.46 (0.04, 3.84) | 1.65 (0.62, 4.47) | 2.15 (0.53, 9.16) | 1.31 (0.44, 3.91) | 0.85 (0.29, 2.43) | 1.75 (0.6, 4.86) | 1.11 (0.44, 2.75) | 3.01 (0.65, 14.49) | 1.02 (0.37, 2.81) | **Erythromycin** |  |  |  |  |  |
| 0.36 (0.12, 1.04) | 0.89 (0.23, 3.67) | 0.56 (0.19, 1.59) | 1.14 (0.08, 14.75) | 0.19 (0.01, 2.01) | 0.52 (0.15, 1.75) | 0.3 (0.03, 2.76) | 1.09 (0.38, 3.08) | 1.4 (0.33, 6.38) | 0.86 (0.27, 2.75) | 0.56 (0.12, 2.49) | 1.15 (0.38, 3.34) | 0.73 (0.24, 2.14) | 1.97 (0.4, 10.39) | 0.67 (0.16, 2.69) | 0.66 (0.18, 2.36) | **Loracarbef** |  |  |  |  |
| NA | NA | NA | NA | NA | NA | NA | NA | NA | NA | NA | NA | NA | NA | NA | NA | NA | **Miocamycin** |  |  |  |
| 0.67 (0.4, 1.1) | 1.64 (0.61, 4.81) | 1.04 (0.63, 1.67) | 2.11 (0.18, 22.89) | 0.36 (0.01, 3.07) | 0.96 (0.44, 2.13) | 0.55 (0.06, 4.16) | **2.01 (1.28, 3.25)** | 2.59 (0.88, 8.56) | 1.6 (0.82, 3.08) | 1.05 (0.3, 3.36) | **2.14 (1.19, 3.6)** | 1.35 (0.79, 2.28) | 3.65 (1, 14.33) | 1.23 (0.45, 3.5) | 1.22 (0.51, 2.9) | 1.84 (0.73, 4.86) | NA | **Penicillin V** |  |  |
| 1.56 (0.24, 14.87) | 3.94 (0.49, 42.21) | 2.44 (0.37, 23.12) | 5.04 (0.25, 130.06) | 0.82 (0.02, 17.03) | 2.25 (0.31, 23.84) | 1.29 (0.07, 25.9) | 4.74 (0.71, 43.98) | 6.11 (0.72, 75.38) | 3.77 (0.54, 36) | 2.48 (0.26, 27.97) | 5.01 (0.72, 46.73) | 3.16 (0.48, 29.24) | 8.73 (0.92, 108.97) | 2.95 (0.36, 32.32) | 2.9 (0.38, 28.94) | 4.36 (0.56, 46.92) | NA | 2.36 (0.38, 20.95) | **Spiramycin** |  |
| 0.46 (0.17, 1.27) | 1.14 (0.32, 4.45) | 0.72 (0.26, 1.93) | 1.47 (0.11, 18.29) | 0.25 (0.01, 2.58) | 0.67 (0.21, 2.14) | 0.38 (0.04, 3.41) | 1.4 (0.52, 3.8) | 1.81 (0.44, 7.98) | 1.11 (0.37, 3.32) | 0.73 (0.17, 3.02) | 1.48 (0.51, 4.07) | 0.94 (0.4, 2.18) | 2.53 (0.55, 12.65) | 0.86 (0.23, 3.24) | 0.84 (0.26, 2.78) | 1.28 (0.35, 4.72) | NA | 0.69 (0.29, 1.67) | 0.29 (0.03, 2.2) | **Telithromycin** |

Values are presented as odds ratios (95% confidence intervals). Penicillin V is used as reference. Statistically significant results are highlighted in bold. NA indicates comparisons with 0% or 100% event rates, resulting in undefined or extremely wide confidence intervals. Abbreviations: AMC, amoxicillin/clavulanate; BPG, Benzathine Penicillin G; BPV, Benzathine Penicillin V.
